# A Five-Gene Signature Predicts Prognosis in Patients with Kidney Renal Clear Cell Carcinoma

**DOI:** 10.1155/2015/842784

**Published:** 2015-10-11

**Authors:** Yueping Zhan, Wenna Guo, Ying Zhang, Qiang Wang, Xin-jian Xu, Liucun Zhu

**Affiliations:** ^1^School of Life Sciences, Shanghai University, Shanghai 200444, China; ^2^Yangzhou Breeding Biological Agriculture Technology Co. Ltd., Yangzhou 225200, China; ^3^State Key Laboratory of Pharmaceutical Biotechnology, School of Life Sciences, Nanjing University, Nanjing 210093, China; ^4^Department of Mathematics, Shanghai University, Shanghai 200444, China

## Abstract

Kidney renal clear cell carcinoma (KIRC) is one of the most common cancers with high mortality all over the world. Many studies have proposed that genes could be used to predict prognosis in KIRC. In this study, RNA expression data from next-generation sequencing and clinical information of 523 patients downloaded from The Cancer Genome Atlas (TCGA) dataset were analyzed in order to identify the relationship between gene expression level and the prognosis of KIRC patients. A set of five genes that significantly associated with overall survival time was identified and a model containing these five genes was constructed by Cox regression analysis. By Kaplan-Meier and Receiver Operating Characteristic (ROC) analysis, we confirmed that the model had good sensitivity and specificity. In summary, expression of the five-gene model is associated with the prognosis outcomes of KIRC patients, and it may have an important clinical significance.

## 1. Introduction

In recent years, the incidence and mortality of kidney cancer have been rising throughout the world [1]. In 2013, nearly 58,000 new cases occurred, and 130,001 patients died of kidney cancer in the United States [2]. Among them, kidney renal clear cell carcinoma (KIRC) is the most common histological subtype and accounts for 70%–80% of renal cancer cases [3]. KIRC tissue is resistant to traditional chemotherapeutic drugs [4], and patient outcomes varied a lot [5]. Although various researches have been done on KIRC, the clinical prognosis of KIRC patients still remains very poor; the survival time of 90% of patients with metastatic KIRC is less than 5 years [6]. Therefore, there is an urgent need to find potential molecular-based prognostic biomarkers in KIRC, and it is also one of the most important steps for prognostic prediction of patients.

Messenger RNA is one of the most common molecular markers. Many studies have suggested that genes were involved in the biological processes of many cancers and related to prognostic survival time of patients. For instance,* SIPL1* (Shank-Interacting Protein-Like 1) has reported to have overexpression during breast cancer tumorigenesis, and inhibiting the expression of* SIPL1* may contribute to inhibition of breast cancer [7].* PLA2G16* has been proved as an important prognostic factor in primary osteosarcoma patients [8].* Dicerl* has been found to be expressed at low level in nasopharyngeal carcinoma tissues no matter whether at the gene or at the protein levels, and it could also be a novel prognostic biomarker [9]. As for KIRC, several studies have been performed to detect gene expression signatures which may provide diagnostic and prognostic information [10–12]. Ge et al. have identified miRNA signature including 22 miRNAs as an independent novel predictor of patient outcomes [13]. Yu et al. have found that the expression of* CIDE* (cell death-inducing DFF45-like effector) is a novel predictor of prognosis [14]. However, detailed analyses of the associations between gene expression level and survival time of patients in KIRC remain limited.

The goal of this paper is identifying genes that are related to overall survival time of KIRC patients by analyzing high-throughput RNA sequencing data downloaded from TCGA [15]. In brief, the main goals are as follows: (1) identify genes that could predict the survival time of KIRC patient, and construct a model; (2) evaluate the prognostic value, sensitivity, and specificity of the model; and (3) investigate the independence and universality of the gene marker in different KIRC stages.

## 2. Materials and Methods

### 2.1. KIRC Gene Expression Data from TCGA

Up to January 2015, TCGA database (https://tcga-data.nci.nih.gov/tcga/) contained 533 KIRC patient samples [15]. The gene expression profiling was performed by using the Illumina HiSeq platforms (Illumina Inc., San Diego, CA, USA). After excluding patients without survival status information, UNC RNASeqV2 level 3 expression data for 523 patients including 20,531 human genes and corresponding clinical data were downloaded. Then the 523 KIRC samples were randomly divided into training set (*n* = 262) and testing set (*n* = 261). Specimen IDs in the two sets were shown in Supplemental Table S1 (in Supplementary Material available online at http://dx.doi.org/10.1155/2015/842784). Training set was used to identify gene expression signature, and the testing set was used for validation.

### 2.2. Statistical Analysis

Firstly, log⁡2 transformed was used for normalizing the RNA-seq expression values [16]. Subsequently, as previous reports [17, 18], genes that were significantly (*p* < 0.001) related to patient survival were identified by Cox regression analysis and random survival forests-variable hunting (RSFVH) algorithm [19]. Considering that a model with a smaller number of genes is generally accompanied with a practically better value, we performed Cox proportional-hazard regression analysis with two genes, three genes, and five genes, respectively, expecting to dig out a better model for predicting survival. Then, based on Cox regression analysis, a risk score formula was built to calculate the risk score for each patient. As reported by Margolin et al. [20] and Meng et al. [18], the survival differences between the low-risk and high-risk groups were evaluated, and the sensitivity and specificity of the model in the survival prediction were also compared.

## 3. Results

### 3.1. Patient Characteristics

All 523 patients used in this study were clinically and pathologically diagnosed with KIRC. Clinical stages of the tumor were classified into stages I to IV based on the Fuhrman nuclear grading system [21]. Here, there are 260 patients from stage 1, 57 patients from stage 2, 125 patients from stage 3, and 81 patients from stage 4, respectively. Additionally, the average age and average prognostic survival time of these 523 patients were 61 years and 902 days, respectively. All the statistical information was summarized in Table 1.

### 3.2. Detection of Genes Associated with Overall Survival Time of KIRC Patients in Training Set

**Figure 1:**
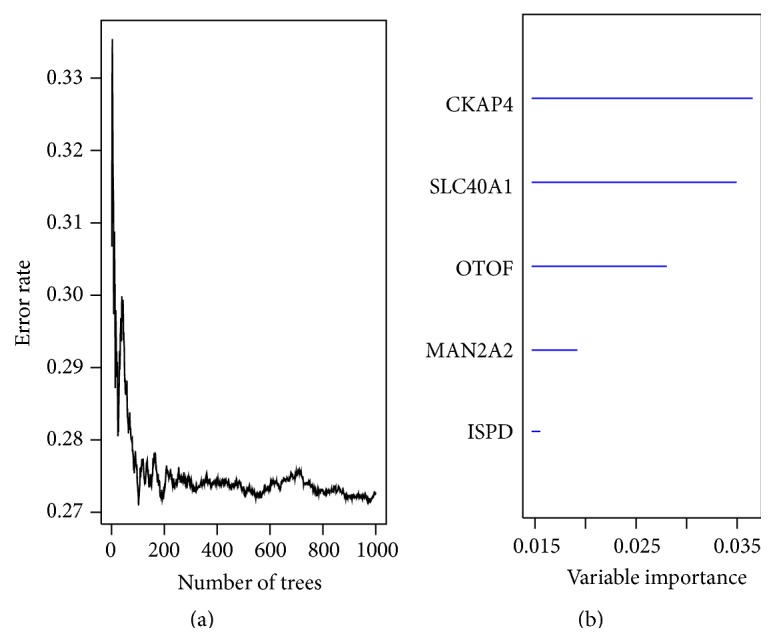
Random survival forests-variable hunting analysis reveals the error rate for the data as a function of trees (a) and the importance values for predictors (b). Importance values show the impact of genes on the model.

**Figure 2:**
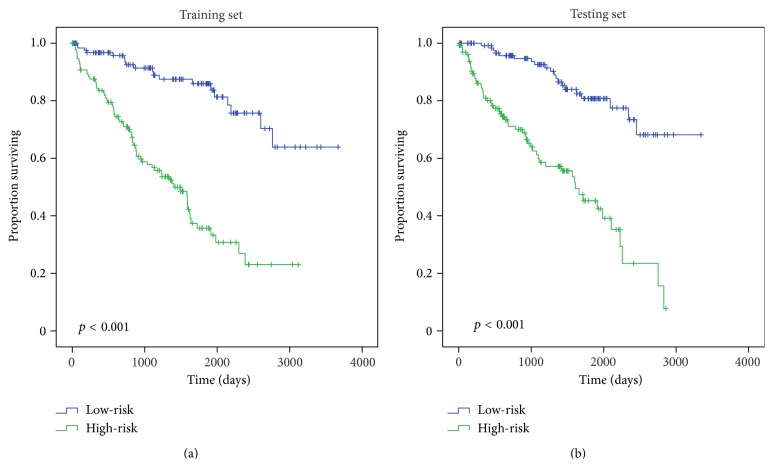
Kaplan-Meier curves with two-sided log-rank test show relationship between the risk score resulting from five genes and patients survival. Using the median risk score as a cut-off, patients were divided into the high-risk score and low-risk score. (a) Kaplan-Meier curves for training set patients (*n* = 262); (b) Kaplan-Meier curves for testing set patients (*n* = 261). The two-sided log-rank tests were used to determine the survival differences between the high-risk score and low-risk score.

To identify the gene which would be potentially associated with overall survival time of patients in KIRC, univariable Cox regression analysis (see Materials and Methods) for gene expression data was conducted in training set. With the significance level of 0.001, a total of 3,849 genes were identified (Table S2). Subsequently, 100 genes with the largest importance value in random survival forests analysis with default parameters [22, 23] were selected. Then, 1–5 genes were chosen from 100 genes as covariates by enumeration algorithm and 79,375,495 models were established in multivariate Cox regression analysis. After comparing with each other, the best model (indexed by AUROC) including 5 genes (*CKAP4, ISPD, MAN2A2, OTOF, and SLC40A1*) was determined, and the risk score formula for this model was (0.422 × expression value of* CKAP4*) + (−0.443 × expression value of* ISPD*) + (0.551 × expression value of* MAN2A2*) + (0.330 × expression value of* OTOF*) + (−0.369 × expression value of* SLC40A1*). The information of these five genes was shown in Table 2. And the functions of these genes were also summarized in Table 3. In addition, the error rate (27.27%) and variable importance values of these five genes were obtained with RSFVH (Figure 1). It can be seen from Figure 1 that the five genes have relatively large importance value;* CKAP4* has more importance than other predictors. Taking the median risk score as the cut-off, the 262 KIRC patients were separated into low-risk group (*n* = 131) and high-risk group (*n* = 131). Survival analysis was performed by using the Kaplan-Meier method with a log-rank statistical test. As shown in Figure 2(a), Kaplan-Meier curves indicated that patients in high-risk group have significantly (*p* < 0.0001) worse prognosis comparing with the low-risk group (Figure 2(a)).

### 3.3. Verification of Survival-Associated Genes in Testing Set

To determine the prognostic potential of the five-gene signature, Kaplan-Meier survival analysis was performed in testing set. Just as it is in training set, based on the risk score of individual patient, patients in testing set were divided into low-risk and high-risk groups and Kaplan-Meier analysis was used to compare the patient survival differences. Statistically significant differences (*p* < 0.0001) between high-risk group and low-risk group were observed; in other words, higher risk score was related to shorter survival time (Figure 2(b)), which is in agreement with that in training set, revealing that five-gene signature may play an important role in predicting the survival of KIRC patients.

**Figure 3:**
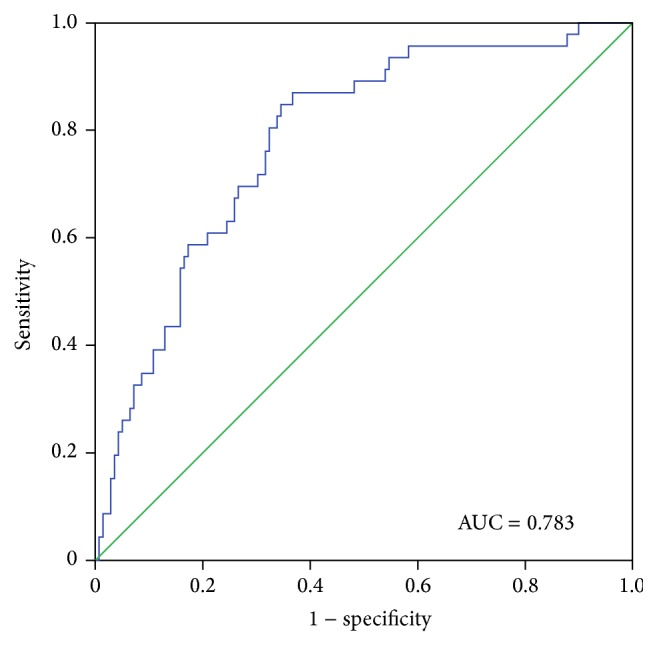
Receiver Operating Characteristic (ROC) analysis of the five-gene signature. The AUROC was 0.783 (*p* < 0.001), showing that the five-gene model has high sensitivity (true positive rate) and specificity (true negative rate) in predicting the survival time of KIRC patients.

To further confirm the clinical performance of the five-gene model as a biomarker for predicting prognosis, the Receiver Operating Characteristic (ROC) analysis was performed for estimating the effect of the gene signature on patient survival. And the corresponding AUROC were calculated by hiring three years as the cut-off point. The AUROC was 0.783 (Figure 3), showing that the five-gene model has high sensitivity and specificity and could be used as a biomarker to predict the prognostic survival of patients.

### 3.4. The Independence and Universality of the Five-Gene Model

Studies have shown that age and clinical stage were also related to patient survival [5, 13, 21]. To examine whether the five-gene signature could distinguish the high-risk patients from low-risk patients when age of patients and stage were taken into account, multivariate Cox proportional hazard analyses were performed in both training and testing set. The results confirmed that risk score of five genes is independent of age and stage, as shown in Table 4. Besides, whether the five-gene signature was functional in different KIIRC stages was also investigated by using Kaplan-Meier and ROC analysis. Results showed that, in stage 3 and stage 4, the survival time of patients was dramatically different between high-risk group and low-risk group (*p* < 0.001, Figure S1). Moreover, the AUROC in stage 2, stage 3, and stage 4 were 0.761, 0.718, and 0.715, respectively (Figure S2), further revealing that the five-gene signature has predictive value in different clinical stages.

## 4. Discussion

KIRC is one of the most common primary renal malignancies with high morbidity and mortality [24]. However, the understanding of KIRC is not complete, and there are no clinical tools for predicting patient outcome apart from the traditional clinical parameters. Accurate data from the clinical examination of KIRC specimens could help doctors to decide appropriate treatment for patients [25]. Therefore, the identification and validation of novel biomarkers account for an important part of practical KIRC study [26]. In this study, we identified a five-gene signature that was significantly related to patient survival in KIRC based on genome-wide RNA profiling of 523 KIRC patients from TCGA database. In addition, we confirmed that the five-gene signature could be regarded as an independent predictor of prognostic survival after considering the various variables including age and stage, and it is also universal in different stages.

Many previous studies on genes in KIRC have mainly considered some known cancer-associated genes. For instance, Wei et al. have found that high expression of pituitary tumor-transforming gene-1 (*PTTG1*) in KIRC patients was associated with poor prognosis by using qRT-PCR and immunohistochemistry [27]. Peters et al. have proved that low gene expression levels of* GATA1* and* GATA2* were related to tumor aggressiveness and short survival time in KIRC [28]. With respect to the five genes we identified in this study, all of them have also been reported to be associated with cancer. It turned out that* CKAP4* could be used to distinguish primary salivary oncocytic lesions from metastatic RCC effectively in dubious cases with 100% accuracy [29] and related to lymphatic metastasis [30, 31]. Mutations in* OTOF*, which functionally triggers membrane fusion and exocytosis, may provide a link between calcium signaling and cancer [22, 32, 33].* SLC40A1* is a cell membrane protein that has been identified to mediate cellular iron efflux [23, 34] and contribute to the invasive phenotype [35]. Mutations in* ISPD* may cause Walker-Warburg syndrome [36, 37].* MAN2A2* was downregulated in hepatocellular carcinoma [38]. However, up to now, such predictive markers were not analyzed in KIRC patients and the molecular study concerning these genes has not been reported in KIRC. Nevertheless, our research showed that the expressions of these genes were related to survival time of patients. ROC curve showed that the AUROC is approximately 0.8, considering that the larger AUROC usually implies a better model for prediction [6, 39], which further demonstrated that the five-gene signature in our study is a novel prognostic marker with high accuracy and has important clinical significance. Furthermore, the five-gene signature was an independent predictor, which was pervasive in different stages. In different stages, ROC analysis shows high sensitivity and specificity (AUROC >0.7) except stage 1, which is possibly because stage 1 is slow-growing tumor, cancer cells are not invasive and metastatic, and the number of patients that died of KIRC was smaller than that in other stages [40]. We found here that the average age of patients who died in stage 1 was more than 67, which is higher than in other stages, revealing that the age at diagnosis may have some influence on KIRC prognosis, and part of deaths was attributed to increased risk of disease mortality with increasing age. Therefore, these results suggested that the five-gene signature is significantly important in clinic. The functional mechanisms of these genes remain unclear. Moreover, the five-gene signature has not yet been tested in a clinical trial. The experimental studies on these genes and further well-designed studies should be conducted to verify our findings, thereby providing a better understanding of their roles in predicting KIRC prognosis.

## 5. Conclusions

In summary, a five-gene signature strongly associated with patients' survival was identified by performing Cox regression analysis and Kaplan-Meier analysis in training set. Subsequently, Kaplan-Meier and ROC analysis in testing set further indicated that the five-gene signature could be used as a novel biomarker to predict the treatment outcome of KIRC patient. Additionally, multivariate Cox regression analysis revealed that the five-gene signature was an independent predictor. These results suggested that the five-gene signature could help to predict the survival with significant clinical implications.

## Supplementary Material

Table S1 The sample list and information of patients in training set and testing set. Table S2 Univariable Cox regression analysis with a significance level of 0.001 reveal significant relation between gene expression and survival time. Figure S1 Kaplan-Meier curves analysis of the different clinical stages in the testing set. The two-sided log-rank test were used to determine the survival differences. Figure S2 Receiver operating characteristic (ROC) analysis of sensitivity and specificity by five-gene model in predicting survival time of patient with different clinical stages in the testing set.

## Figures and Tables

**Table 1:** Summary of patient demographics and clinical characteristics.

Characteristic	Patients			
	Training set	Testing set	Total	
Age				
Median	61	60	61	
Range	26–90	29–90	26–90	
Sex				
Male	164	174	338	64.63%
Female	98	87	185	35.37%
Vital status				
Living	173	184	357	68.26%
Dead	89	77	166	31.74%
Clinical stage				
Stage I	134	126	260	49.71%
Stage II	22	35	57	10.9%
Stage III	72	53	125	23.9%
Stage IV	34	47	81	15.49%

**Table 2:** Five genes significantly associated with the survival time of patients in the training set (*n* = 262).

Gene name	Parametric *p* value	Hazard ratio	Coefficient	Variable importance	Relative importance
CKAP4	1.80*E* − 09	1.525	0.422	0.0365	1
SLC40A1	9.30*E* − 08	0.691	−0.369	0.036	0.9862
OTOF	4.60*E* − 10	1.391	0.33	0.28	0.7674
MAN2A2	0.00085	1.734	0.551	0.0192	0.5249
ISPD	1.70*E* − 05	0.642	−0.443	0.0147	0.4012

**Table 3:** Five-gene functions' analysis.

Gene name	Chromosomal position	Start site	End site	Function	Study
CKAP4	chr12	106237881	106247935	Sequence specific DNA binding transcriptional activator or repressor	McHugh et al. [29] Li et al. [30] Zhang et al. [31]

ISPD	chr7	15916851	16530558	Mutations in ISPD cause Walker-Warburg syndrome	Willer et al. [36] Roscioli et al. [37]

MAN2A2	chr15	90902218	90922585	Catalyzes the committed step in the biosynthesis of complex N-glycans	Kroes et al. [38]

OTOF	chr2	26457203	26558698	Triggers membrane fusion and exocytosis	Padmanarayana et al. [33] Yildirim-Baylan et al. [22]

SLC40A1	chr2	189560590	189580811	Mediates cellular iron efflux	Moreno-Carralero et al. [34]

**Table 4:** Univariable and multivariable Cox regression analyses in training and testing set.

Variables	Univariable model			Multivariable model		
	HR	95% CI of HR	*p* value	HR	95% CI of HR	*p* value
Training set (*N* = 262)						
Five-gene model	2.717	2.180–3.387	<0.001	2.752	2.193–3.454	<0.001
Age	1.031	1.014–1.050	0.001	1.032	1.009–1.048	0.003
Testing set (*N* = 261)						
Five-gene model	1.936	1.620–2.315	<0.001	1.875	1.560–2.253	<0.001
Age	1.022	1.004–1.041	0.019	1.011	0.993–1.031	0.234

Training set (*N* = 262)						
Five-gene model	2.717	2.180–3.387	<0.001	2.193	1.726–2.786	<0.001
Stage	2.097	1.734–2.537	<0.001	1.717	1.394–2.111	<0.001
Testing set (*N* = 261)						
Five-gene model	1.936	1.620–2.315	<0.001	1.700	1.390–2.078	<0.001
Stage	1.905	1.564–2.322	<0.001	1.679	1.367–2.062	<0.001

## Abbreviations

KIRC: Kidney renal clear cell carcinoma
TCGA: The Cancer Genome Atlas
RSFVH: Random survival forests-variable hunting
ROC: Receiver Operating Characteristic.
