# Supplementary material for: A Five-Gene Signature Predicts Prognosis in Patients with Kidney Renal Clear Cell Carcinoma
Source: Comput Math Methods Med. 2015 Oct 11;2015:842784. doi: 10.1155/2015/842784 (PMC4619904; doi:10.1155/2015/842784)
Supplement: Supplementary file 1 — Table S1 The sample list and information of patients in training set and testing set. Table S2 Univariable Cox regression analysis with a significance level of 0.001 reveal significant relation between gene expression and survival time. Figure S1 Kaplan-Meier curves analysis of the different clinical stages in the testing set. The two-sided log-rank test were used to determine the survival differences. Figure S2 Receiver operating characteristic (ROC) analysis of sensitivity and specificity by five-gene model in predicting survival time of patient with different clinical stages in the testing set. [file 842784.f1.zip › TableS1.docx]

**Table S1** The sample list and information of patients in training set and testing set

| Training set | | | | |  | Testing set | | | | |
| --- | --- | --- | --- | --- | --- | --- | --- | --- | --- | --- |
| TCGA_ID | status | time | Stage | Age |  | TCGA_ID | status | time | Stage | Age |
| TCGA-BP-4986 | 0 | 785 | StageI | 75 |  | TCGA-A3-3306 | 0 | 1120 | StageI | 67 |
| TCGA-B0-4824 | 1 | 1657 | StageI | 49 |  | TCGA-B0-4848 | 1 | 883 | StageIII | 54 |
| TCGA-A3-A6NN | 0 | 3 | StageI | 78 |  | TCGA-B0-5077 | 1 | 1317 | StageI | 77 |
| TCGA-A3-3319 | 0 | 1130 | StageI | 70 |  | TCGA-B0-5080 | 1 | 342 | StageIV | 63 |
| TCGA-BP-4965 | 0 | 1871 | StageI | 46 |  | TCGA-B0-5083 | 1 | 1045 | StageI | 63 |
| TCGA-CZ-5986 | 0 | 373 | StageI | 61 |  | TCGA-B0-5084 | 1 | 222 | StageIV | 33 |
| TCGA-BP-4759 | 0 | 2372 | StageI | 50 |  | TCGA-B0-5088 | 1 | 563 | StageI | 53 |
| TCGA-CW-5583 | 0 | 2489 | StageI | 51 |  | TCGA-B0-5092 | 1 | 459 | StageIV | 53 |
| TCGA-BP-4784 | 0 | 1854 | StageI | 67 |  | TCGA-B0-5095 | 1 | 245 | StageIII | 81 |
| TCGA-A3-3349 | 0 | 1385 | StageI | 34 |  | TCGA-B0-5097 | 0 | 665 | StageIII | 59 |
| TCGA-BP-4790 | 1 | 1111 | StageI | 76 |  | TCGA-A3-3324 | 0 | 1186 | StageI | 51 |
| TCGA-AS-3777 | 0 | 1238 | StageI | 63 |  | TCGA-B0-5104 | 1 | 2752 | StageI | 90 |
| TCGA-BP-4161 | 0 | 2746 | StageI | 74 |  | TCGA-B0-5106 | 1 | 1598 | StageI | 64 |
| TCGA-BP-5180 | 0 | 2263 | StageI | 53 |  | TCGA-B0-5107 | 1 | 927 | StageIV | 65 |
| TCGA-AK-3465 | 0 | 369 | StageI | 71 |  | TCGA-B0-5110 | 0 | 1092 | StageI | 71 |
| TCGA-BP-5189 | 1 | 822 | StageI | 60 |  | TCGA-B0-5121 | 0 | 554 | StageI | 56 |
| TCGA-B0-4838 | 1 | 834 | StageI | 69 |  | TCGA-B0-5399 | 0 | 652 | StageI | 46 |
| TCGA-B0-5695 | 0 | 1420 | StageI | 61 |  | TCGA-B0-5402 | 0 | 449 | StageIV | 64 |
| TCGA-CJ-5672 | 1 | 2190 | StageI | 84 |  | TCGA-A3-3328 | 0 | 1385 | StageI | 79 |
| TCGA-B0-5698 | 0 | 2583 | StageI | 77 |  | TCGA-B0-5692 | 0 | 1487 | StageIII | 66 |
| TCGA-B0-5705 | 0 | 3668 | StageI | 65 |  | TCGA-B0-5694 | 1 | 480 | StageIII | 71 |
| TCGA-BP-4969 | 0 | 1794 | StageI | 63 |  | TCGA-B0-5696 | 0 | 1727 | StageIII | 69 |
| TCGA-A3-3322 | 0 | 1478 | StageI | 51 |  | TCGA-B0-5697 | 0 | 1835 | StageI | 50 |
| TCGA-CJ-4892 | 0 | 1521 | StageI | 65 |  | TCGA-B0-5699 | 0 | 2741 | StageI | 53 |
| TCGA-B0-5690 | 0 | 2408 | StageI | 53 |  | TCGA-A3-3329 | 0 | 706 | StageI | 75 |
| TCGA-B0-5117 | 0 | 535 | StageI | 40 |  | TCGA-B0-5706 | 0 | 2414 | StageII | 45 |
| TCGA-B8-5552 | 0 | 1046 | StageI | 41 |  | TCGA-B0-5710 | 0 | 1459 | StageI | 57 |
| TCGA-B0-5693 | 0 | 3076 | StageI | 47 |  | TCGA-B0-5713 | 0 | 1865 | StageIII | 75 |
| TCGA-B2-4099 | 0 | 374 | StageI | 83 |  | TCGA-B0-5812 | 0 | 2963 | StageI | 53 |
| TCGA-BP-4987 | 0 | 1124 | StageI | 41 |  | TCGA-B2-3924 | 0 | 1092 | StageI | 73 |
| TCGA-3Z-A93Z | 0 | 385 | StageI | 69 |  | TCGA-B2-4098 | 1 | 51 | StageI | 72 |
| TCGA-B8-4619 | 0 | 523 | StageI | 58 |  | TCGA-B2-4101 | 0 | 648 | StageII | 52 |
| TCGA-BP-4162 | 0 | 3074 | StageI | 65 |  | TCGA-B2-4102 | 0 | 952 | StageI | 61 |
| TCGA-B8-5549 | 0 | 194 | StageI | 53 |  | TCGA-B2-5636 | 0 | 919 | StageI | 79 |
| TCGA-A3-3387 | 0 | 617 | StageI | 49 |  | TCGA-B2-5639 | 1 | 1003 | StageIV | 46 |
| TCGA-BP-5008 | 0 | 1071 | StageI | 46 |  | TCGA-A3-3335 | 0 | 665 | StageII | 41 |
| TCGA-BP-5176 | 1 | 1590 | StageI | 78 |  | TCGA-B4-5378 | 0 | 175 | StageI | 62 |
| TCGA-B0-4837 | 1 | 1378 | StageI | 63 |  | TCGA-B4-5835 | 0 | 16 | StageI | 64 |
| TCGA-CZ-4862 | 0 | 1843 | StageI | 46 |  | TCGA-B4-5836 | 0 | 141 | StageI | 61 |
| TCGA-B0-5707 | 0 | 2828 | StageI | 39 |  | TCGA-B4-5838 | 0 | 166 | StageIII | 52 |
| TCGA-B0-5102 | 1 | 2764 | StageI | 74 |  | TCGA-B4-5843 | 0 | 11 | StageI | 45 |
| TCGA-B2-5635 | 0 | 315 | StageI | 74 |  | TCGA-B4-5844 | 0 | 7 | StageII | 61 |
| TCGA-GK-A6C7 | 0 | 61 | StageI | 76 |  | TCGA-A3-3343 | 0 | 945 | StageII | 79 |
| TCGA-BP-5174 | 0 | 2257 | StageI | 45 |  | TCGA-B8-4146 | 0 | 511 | StageI | 41 |
| TCGA-B8-4148 | 0 | 826 | StageI | 63 |  | TCGA-B8-4154 | 0 | 750 | StageI | 73 |
| TCGA-BP-5177 | 0 | 293 | StageI | 46 |  | TCGA-A3-3347 | 1 | 1610 | StageIII | 76 |
| TCGA-BP-5001 | 0 | 1177 | StageI | 43 |  | TCGA-B8-5159 | 0 | 722 | StageI | 61 |
| TCGA-B0-4839 | 1 | 1639 | StageI | 80 |  | TCGA-B8-5162 | 0 | 36 | StageII | 62 |
| TCGA-DV-5573 | 0 | 1130 | StageI | 41 |  | TCGA-B8-5163 | 0 | 822 | StageIII | 63 |
| TCGA-CJ-4908 | 0 | 1531 | StageI | 38 |  | TCGA-B8-5165 | 0 | 737 | StageI | 43 |
| TCGA-BP-5006 | 0 | 840 | StageI | 61 |  | TCGA-B8-5545 | 0 | 522 | StageI | 42 |
| TCGA-B8-A7U6 | 0 | 110 | StageI | 54 |  | TCGA-B8-5550 | 0 | 434 | StageIII | 71 |
| TCGA-BP-4988 | 1 | 828 | StageI | 72 |  | TCGA-A3-3307 | 0 | 1436 | StageIII | 66 |
| TCGA-A3-3320 | 0 | 1508 | StageI | 52 |  | TCGA-BP-4160 | 0 | 2881 | StageIII | 67 |
| TCGA-BP-5009 | 1 | 1102 | StageI | 52 |  | TCGA-BP-4163 | 0 | 2839 | StageIII | 60 |
| TCGA-BP-5195 | 0 | 749 | StageI | 75 |  | TCGA-BP-4164 | 1 | 992 | StageIII | 51 |
| TCGA-A3-3331 | 0 | 1485 | StageI | 86 |  | TCGA-A3-3351 | 0 | 910 | StageII | 42 |
| TCGA-DV-5576 | 1 | 727 | StageI | 55 |  | TCGA-BP-4166 | 0 | 13 | StageIII | 69 |
| TCGA-B2-5641 | 0 | 656 | StageI | 79 |  | TCGA-BP-4167 | 0 | 2718 | StageIII | 59 |
| TCGA-BP-5194 | 0 | 408 | StageI | 39 |  | TCGA-BP-4170 | 1 | 2343 | StageI | 72 |
| TCGA-A3-3380 | 0 | 567 | StageI | 54 |  | TCGA-BP-4325 | 0 | 2964 | StageI | 64 |
| TCGA-CZ-4853 | 0 | 774 | StageI | 82 |  | TCGA-BP-4337 | 1 | 2 | StageIII | 76 |
| TCGA-BP-5184 | 0 | 1133 | StageI | 54 |  | TCGA-BP-4338 | 0 | 2859 | StageI | 43 |
| TCGA-B2-5633 | 0 | 358 | StageI | 56 |  | TCGA-A3-3357 | 0 | 2688 | StageII | 62 |
| TCGA-DV-5568 | 0 | 370 | StageI | 26 |  | TCGA-BP-4342 | 1 | 2256 | StageII | 79 |
| TCGA-BP-4177 | 0 | 1670 | StageI | 65 |  | TCGA-BP-4344 | 0 | 1666 | StageI | 75 |
| TCGA-BP-4992 | 0 | 501 | StageI | 66 |  | TCGA-BP-4349 | 0 | 372 | StageI | 68 |
| TCGA-B0-5702 | 0 | 1605 | StageI | 71 |  | TCGA-BP-4351 | 0 | 970 | StageIII | 51 |
| TCGA-CW-5581 | 0 | 2799 | StageI | 44 |  | TCGA-BP-4352 | 1 | 344 | StageIV | 74 |
| TCGA-AS-3778 | 0 | 43 | StageI | 35 |  | TCGA-BP-4353 | 1 | 375 | StageI | 61 |
| TCGA-BP-4975 | 0 | 1433 | StageI | 40 |  | TCGA-A3-3358 | 0 | 1307 | StageI | 57 |
| TCGA-CJ-4872 | 0 | 326 | StageI | 51 |  | TCGA-BP-4756 | 0 | 374 | StageI | 62 |
| TCGA-BP-4789 | 0 | 1489 | StageI | 48 |  | TCGA-BP-4758 | 0 | 2208 | StageI | 40 |
| TCGA-BP-4999 | 0 | 1266 | StageI | 56 |  | TCGA-BP-4760 | 0 | 2361 | StageI | 69 |
| TCGA-CJ-4899 | 0 | 1528 | StageI | 42 |  | TCGA-BP-4761 | 0 | 182 | StageIII | 57 |
| TCGA-CJ-4905 | 0 | 1496 | StageI | 62 |  | TCGA-BP-4762 | 1 | 1343 | StageI | 42 |
| TCGA-AK-3427 | 0 | 2566 | StageI | 65 |  | TCGA-BP-4763 | 1 | 1270 | StageI | 79 |
| TCGA-CW-6088 | 0 | 3222 | StageI | 60 |  | TCGA-BP-4765 | 0 | 2184 | StageI | 43 |
| TCGA-BP-4801 | 0 | 1124 | StageI | 57 |  | TCGA-A3-3359 | 0 | 2504 | StageI | 82 |
| TCGA-A3-3323 | 0 | 1106 | StageI | 53 |  | TCGA-BP-4766 | 0 | 1462 | StageI | 43 |
| TCGA-CW-5589 | 0 | 2378 | StageI | 52 |  | TCGA-BP-4768 | 0 | 400 | StageI | 72 |
| TCGA-DV-5567 | 0 | 2004 | StageI | 40 |  | TCGA-BP-4769 | 0 | 1876 | StageI | 63 |
| TCGA-A3-3325 | 0 | 751 | StageI | 52 |  | TCGA-BP-4771 | 1 | 162 | StageIV | 62 |
| TCGA-CZ-4854 | 1 | 1404 | StageI | 68 |  | TCGA-BP-4774 | 0 | 1885 | StageI | 57 |
| TCGA-DV-5565 | 0 | 1329 | StageI | 59 |  | TCGA-BP-4775 | 0 | 1843 | StageI | 55 |
| TCGA-B8-5553 | 0 | 435 | StageI | 67 |  | TCGA-BP-4777 | 0 | 1731 | StageI | 46 |
| TCGA-CJ-6027 | 0 | 1855 | StageI | 77 |  | TCGA-BP-4782 | 0 | 354 | StageI | 55 |
| TCGA-A3-A8CQ | 0 | 3 | StageI | 59 |  | TCGA-A3-3362 | 0 | 1559 | StageI | 60 |
| TCGA-DV-5566 | 0 | 1398 | StageI | 67 |  | TCGA-BP-4787 | 1 | 480 | StageIV | 59 |
| TCGA-CZ-4859 | 0 | 1787 | StageI | 59 |  | TCGA-BP-4795 | 0 | 620 | StageI | 74 |
| TCGA-CZ-4866 | 0 | 1868 | StageI | 79 |  | TCGA-BP-4797 | 0 | 1107 | StageIII | 34 |
| TCGA-BP-4158 | 0 | 3377 | StageI | 69 |  | TCGA-BP-4803 | 0 | 204 | StageIII | 79 |
| TCGA-BP-4165 | 0 | 3037 | StageI | 64 |  | TCGA-BP-4804 | 0 | 1459 | StageI | 59 |
| TCGA-CZ-5984 | 0 | 2067 | StageI | 51 |  | TCGA-BP-4807 | 0 | 211 | StageI | 42 |
| TCGA-CZ-5982 | 0 | 2439 | StageI | 59 |  | TCGA-BP-4960 | 0 | 2172 | StageII | 46 |
| TCGA-B0-5119 | 0 | 59 | StageI | 61 |  | TCGA-BP-4961 | 0 | 1935 | StageI | 47 |
| TCGA-B0-5703 | 0 | 1203 | StageI | 73 |  | TCGA-BP-4962 | 0 | 1785 | StageII | 58 |
| TCGA-MW-A4EC | 0 | 330 | StageI | 72 |  | TCGA-BP-4963 | 0 | 1834 | StageI | 63 |
| TCGA-A3-A8OX | 0 | 0 | StageI | 65 |  | TCGA-BP-4964 | 0 | 1862 | StageI | 54 |
| TCGA-B0-4833 | 1 | 2386 | StageI | 82 |  | TCGA-BP-4968 | 0 | 1746 | StageI | 40 |
| TCGA-CJ-4874 | 0 | 2283 | StageI | 73 |  | TCGA-BP-4971 | 0 | 1487 | StageIII | 40 |
| TCGA-CZ-4856 | 0 | 18 | StageI | 62 |  | TCGA-BP-4972 | 0 | 1502 | StageIII | 43 |
| TCGA-BP-4176 | 0 | 1955 | StageI | 64 |  | TCGA-BP-4973 | 0 | 1384 | StageIII | 47 |
| TCGA-B8-4621 | 0 | 788 | StageI | 63 |  | TCGA-BP-4974 | 1 | 210 | StageIV | 58 |
| TCGA-BP-5186 | 0 | 693 | StageI | 50 |  | TCGA-BP-4976 | 0 | 1632 | StageI | 77 |
| TCGA-BP-4326 | 1 | 1625 | StageI | 53 |  | TCGA-BP-4977 | 0 | 454 | StageI | 57 |
| TCGA-A3-A6NL | 0 | 689 | StageI | 49 |  | TCGA-A3-3367 | 0 | 2270 | StageI | 72 |
| TCGA-B8-5551 | 0 | 16 | StageI | 65 |  | TCGA-BP-4981 | 1 | 1097 | StageIII | 75 |
| TCGA-BP-4982 | 0 | 1014 | StageI | 42 |  | TCGA-BP-4983 | 0 | 1413 | StageIII | 67 |
| TCGA-BP-4159 | 1 | 2601 | StageI | 70 |  | TCGA-BP-4985 | 1 | 952 | StageIII | 72 |
| TCGA-BP-4340 | 1 | 562 | StageI | 70 |  | TCGA-BP-4991 | 0 | 1413 | StageI | 54 |
| TCGA-A3-3383 | 0 | 861 | StageI | 52 |  | TCGA-A3-3308 | 0 | 16 | StageIII | 77 |
| TCGA-A3-3313 | 1 | 735 | StageI | 59 |  | TCGA-A3-3370 | 0 | 2274 | StageI | 48 |
| TCGA-B8-5546 | 0 | 505 | StageI | 38 |  | TCGA-BP-4993 | 0 | 177 | StageI | 58 |
| TCGA-AK-3450 | 0 | 1779 | StageI | 85 |  | TCGA-BP-4994 | 0 | 1308 | StageI | 54 |
| TCGA-B0-4945 | 1 | 2145 | StageI | 75 |  | TCGA-BP-4995 | 0 | 1371 | StageI | 68 |
| TCGA-B4-5834 | 0 | 38 | StageI | 59 |  | TCGA-BP-4998 | 0 | 932 | StageI | 49 |
| TCGA-A3-3385 | 0 | 1993 | StageI | 46 |  | TCGA-BP-5000 | 0 | 563 | StageI | 40 |
| TCGA-CJ-5671 | 0 | 1943 | StageI | 51 |  | TCGA-BP-5004 | 0 | 1126 | StageI | 53 |
| TCGA-BP-4781 | 0 | 2080 | StageI | 78 |  | TCGA-A3-3372 | 0 | 735 | StageIII | 64 |
| TCGA-B0-5098 | 1 | 1584 | StageI | 53 |  | TCGA-BP-5168 | 1 | 1463 | StageI | 75 |
| TCGA-AK-3440 | 0 | 1745 | StageI | 58 |  | TCGA-BP-5169 | 0 | 193 | StageI | 70 |
| TCGA-CW-6093 | 0 | 3146 | StageI | 73 |  | TCGA-BP-5170 | 0 | 2412 | StageI | 55 |
| TCGA-BP-4959 | 0 | 2660 | StageI | 49 |  | TCGA-BP-5173 | 1 | 53 | StageI | 75 |
| TCGA-B0-5691 | 0 | 3431 | StageI | 66 |  | TCGA-BP-5175 | 0 | 932 | StageI | 60 |
| TCGA-B0-5700 | 0 | 1082 | StageI | 77 |  | TCGA-A3-3373 | 0 | 1621 | StageI | 54 |
| TCGA-A3-3365 | 0 | 873 | StageI | 46 |  | TCGA-BP-5178 | 1 | 1912 | StageIV | 71 |
| TCGA-CJ-5689 | 1 | 1620 | StageI | 90 |  | TCGA-BP-5181 | 0 | 1495 | StageI | 58 |
| TCGA-CJ-4889 | 0 | 1946 | StageI | 63 |  | TCGA-BP-5182 | 0 | 1165 | StageI | 56 |
| TCGA-CZ-4865 | 1 | 166 | StageI | 70 |  | TCGA-BP-5187 | 0 | 406 | StageI | 54 |
| TCGA-B0-5120 | 0 | 493 | StageI | 72 |  | TCGA-BP-5190 | 0 | 1011 | StageI | 61 |
| TCGA-CJ-6030 | 1 | 2299 | StageI | 65 |  | TCGA-BP-5192 | 0 | 714 | StageI | 59 |
| TCGA-BP-4776 | 0 | 411 | StageI | 52 |  | TCGA-BP-5196 | 0 | 1018 | StageI | 53 |
| TCGA-A3-3374 | 0 | 1314 | StageI | 51 |  | TCGA-BP-5198 | 0 | 603 | StageIII | 72 |
| TCGA-BP-4174 | 0 | 1879 | StageII | 49 |  | TCGA-CJ-4634 | 0 | 1820 | StageI | 60 |
| TCGA-CZ-5985 | 0 | 1997 | StageII | 58 |  | TCGA-A3-3378 | 0 | 630 | StageI | 60 |
| TCGA-CJ-5675 | 0 | 2430 | StageII | 70 |  | TCGA-CJ-4635 | 0 | 1416 | StageI | 48 |
| TCGA-CZ-5989 | 0 | 1905 | StageII | 60 |  | TCGA-CJ-4636 | 0 | 1924 | StageIII | 51 |
| TCGA-BP-4173 | 0 | 1893 | StageII | 47 |  | TCGA-CJ-4637 | 1 | 2227 | StageIV | 52 |
| TCGA-CZ-5469 | 1 | 946 | StageII | 41 |  | TCGA-CJ-4639 | 0 | 2308 | StageII | 49 |
| TCGA-B0-4852 | 1 | 1121 | StageII | 78 |  | TCGA-CJ-4640 | 0 | 1998 | StageIII | 49 |
| TCGA-BP-5200 | 0 | 1063 | StageII | 44 |  | TCGA-CJ-4641 | 1 | 1661 | StageIV | 55 |
| TCGA-BP-5199 | 0 | 1355 | StageII | 58 |  | TCGA-CJ-4643 | 0 | 1793 | StageII | 67 |
| TCGA-A3-3363 | 0 | 319 | StageII | 50 |  | TCGA-CJ-4644 | 1 | 336 | StageIV | 48 |
| TCGA-A3-3317 | 0 | 1491 | StageII | 67 |  | TCGA-CJ-4868 | 1 | 646 | StageIV | 42 |
| TCGA-BP-5007 | 0 | 1140 | StageII | 45 |  | TCGA-CJ-4871 | 0 | 2423 | StageIV | 63 |
| TCGA-BP-4169 | 1 | 701 | StageII | 76 |  | TCGA-CJ-4875 | 0 | 2353 | StageIV | 67 |
| TCGA-CJ-4912 | 0 | 1657 | StageII | 61 |  | TCGA-CJ-4876 | 0 | 1955 | StageII | 57 |
| TCGA-CZ-5451 | 0 | 1929 | StageII | 74 |  | TCGA-A3-3382 | 0 | 574 | StageI | 69 |
| TCGA-CZ-5452 | 0 | 1789 | StageII | 69 |  | TCGA-CJ-4885 | 0 | 2125 | StageIV | 64 |
| TCGA-CZ-5470 | 0 | 386 | StageII | 72 |  | TCGA-CJ-4886 | 0 | 1952 | StageI | 42 |
| TCGA-BP-4327 | 1 | 109 | StageII | 75 |  | TCGA-CJ-4887 | 1 | 932 | StageIV | 48 |
| TCGA-AK-3431 | 0 | 1853 | StageII | 62 |  | TCGA-CJ-4888 | 1 | 1567 | StageIV | 59 |
| TCGA-AK-3433 | 0 | 2192 | StageII | 48 |  | TCGA-CJ-4893 | 0 | 750 | StageI | 76 |
| TCGA-AK-3443 | 0 | 1423 | StageII | 45 |  | TCGA-CJ-4895 | 1 | 1200 | StageIV | 62 |
| TCGA-B2-3923 | 0 | 992 | StageII | 59 |  | TCGA-CJ-4900 | 1 | 1714 | StageIV | 69 |
| TCGA-BP-4345 | 0 | 1516 | StageIII | 62 |  | TCGA-CJ-4902 | 0 | 1520 | StageIII | 61 |
| TCGA-BP-5191 | 0 | 967 | StageIII | 79 |  | TCGA-CJ-4903 | 0 | 1559 | StageI | 50 |
| TCGA-CJ-4882 | 0 | 1883 | StageIII | 57 |  | TCGA-CJ-4904 | 0 | 1792 | StageIV | 60 |
| TCGA-B0-5711 | 0 | 2931 | StageIII | 50 |  | TCGA-CJ-4907 | 0 | 1499 | StageIII | 58 |
| TCGA-B0-4813 | 1 | 18 | StageIII | 68 |  | TCGA-CJ-4916 | 0 | 1373 | StageIII | 69 |
| TCGA-BP-4329 | 1 | 845 | StageIII | 75 |  | TCGA-CJ-4920 | 1 | 139 | StageI | 64 |
| TCGA-CJ-4884 | 0 | 1759 | StageIII | 72 |  | TCGA-CJ-4923 | 1 | 572 | StageIV | 63 |
| TCGA-BP-5010 | 1 | 878 | StageIII | 63 |  | TCGA-CJ-5676 | 0 | 2575 | StageIII | 47 |
| TCGA-B0-5081 | 1 | 362 | StageIII | 79 |  | TCGA-CJ-5677 | 1 | 782 | StageIV | 54 |
| TCGA-BP-4967 | 0 | 205 | StageIII | 76 |  | TCGA-CJ-5679 | 1 | 679 | StageIII | 73 |
| TCGA-B0-5701 | 0 | 1732 | StageIII | 65 |  | TCGA-CJ-5680 | 1 | 768 | StageIV | 65 |
| TCGA-CZ-5458 | 0 | 1732 | StageIII | 43 |  | TCGA-CJ-5682 | 0 | 1883 | StageIV | 60 |
| TCGA-AK-3428 | 0 | 2223 | StageIII | 62 |  | TCGA-CJ-5683 | 0 | 1889 | StageI | 78 |
| TCGA-B0-5075 | 1 | 637 | StageIII | 77 |  | TCGA-CJ-5686 | 0 | 2038 | StageI | 59 |
| TCGA-B8-5164 | 0 | 26 | StageIII | 65 |  | TCGA-A3-3311 | 1 | 1191 | StageI | 57 |
| TCGA-BP-4334 | 1 | 645 | StageIII | 56 |  | TCGA-CJ-6028 | 1 | 1625 | StageIV | 58 |
| TCGA-B0-5709 | 0 | 3117 | StageIII | 62 |  | TCGA-CJ-6031 | 0 | 1906 | StageI | 54 |
| TCGA-B0-5113 | 0 | 359 | StageIII | 69 |  | TCGA-CJ-6032 | 0 | 2548 | StageII | 63 |
| TCGA-CJ-4894 | 1 | 841 | StageIII | 58 |  | TCGA-AK-3429 | 0 | 2017 | StageII | 54 |
| TCGA-CW-6097 | 1 | 571 | StageIII | 32 |  | TCGA-CW-5584 | 1 | 164 | StageIII | 74 |
| TCGA-B0-5400 | 0 | 1132 | StageIII | 59 |  | TCGA-CW-5585 | 0 | 2609 | StageIV | 51 |
| TCGA-CZ-5466 | 0 | 685 | StageIII | 67 |  | TCGA-CW-5587 | 0 | 2226 | StageIII | 62 |
| TCGA-CJ-5684 | 0 | 2231 | StageIII | 61 |  | TCGA-CW-5590 | 1 | 1075 | StageIV | 51 |
| TCGA-BP-4970 | 0 | 433 | StageIII | 44 |  | TCGA-CW-6090 | 0 | 2552 | StageI | 68 |
| TCGA-BP-4355 | 1 | 953 | StageIII | 59 |  | TCGA-CZ-4857 | 1 | 1432 | StageIV | 56 |
| TCGA-CJ-4897 | 0 | 1808 | StageIII | 79 |  | TCGA-CZ-4858 | 1 | 2105 | StageII | 39 |
| TCGA-BP-4341 | 1 | 1589 | StageIII | 67 |  | TCGA-CZ-4861 | 1 | 446 | StageII | 63 |
| TCGA-B0-4810 | 1 | 478 | StageIII | 47 |  | TCGA-CZ-4863 | 0 | 1928 | StageIII | 51 |
| TCGA-CJ-4873 | 0 | 2259 | StageIII | 85 |  | TCGA-CZ-4864 | 1 | 2830 | StageII | 86 |
| TCGA-B0-4706 | 1 | 65 | StageIII | 61 |  | TCGA-CZ-5453 | 0 | 25 | StageII | 67 |
| TCGA-BP-4330 | 0 | 1888 | StageIII | 60 |  | TCGA-CZ-5455 | 1 | 561 | StageIV | 63 |
| TCGA-B0-4821 | 1 | 1230 | StageIII | 68 |  | TCGA-CZ-5457 | 0 | 1683 | StageIII | 62 |
| TCGA-BP-4343 | 1 | 1912 | StageIII | 64 |  | TCGA-AK-3434 | 0 | 2087 | StageI | 72 |
| TCGA-A3-3352 | 1 | 561 | StageIII | 74 |  | TCGA-CZ-5460 | 0 | 1430 | StageIV | 55 |
| TCGA-BP-4346 | 1 | 1493 | StageIII | 57 |  | TCGA-CZ-5461 | 1 | 330 | StageIV | 52 |
| TCGA-B0-5115 | 0 | 797 | StageIII | 43 |  | TCGA-CZ-5462 | 1 | 311 | StageIV | 83 |
| TCGA-B0-4843 | 1 | 320 | StageIII | 57 |  | TCGA-CZ-5463 | 0 | 662 | StageII | 76 |
| TCGA-BP-4347 | 0 | 1367 | StageIII | 74 |  | TCGA-CZ-5464 | 0 | 1740 | StageIV | 69 |
| TCGA-B0-5096 | 1 | 68 | StageIII | 72 |  | TCGA-CZ-5465 | 0 | 1446 | StageIII | 76 |
| TCGA-B0-4827 | 1 | 885 | StageIII | 77 |  | TCGA-AK-3436 | 0 | 2044 | StageIV | 40 |
| TCGA-B8-4620 | 0 | 777 | StageIII | 70 |  | TCGA-CZ-5988 | 0 | 693 | StageI | 38 |
| TCGA-B4-5832 | 0 | 7 | StageIII | 65 |  | TCGA-DV-5569 | 0 | 355 | StageI | 29 |
| TCGA-B0-5099 | 1 | 485 | StageIII | 88 |  | TCGA-DV-5574 | 0 | 2016 | StageI | 37 |
| TCGA-B0-5109 | 1 | 587 | StageIII | 69 |  | TCGA-DV-5575 | 0 | 1729 | StageI | 52 |
| TCGA-CZ-5467 | 1 | 73 | StageIII | 86 |  | TCGA-B0-4691 | 1 | 139 | StageIV | 55 |
| TCGA-CJ-4878 | 0 | 2186 | StageIII | 71 |  | TCGA-AK-3454 | 0 | 874 | StageI | 84 |
| TCGA-B0-4849 | 1 | 69 | StageIII | 51 |  | TCGA-AK-3453 | 0 | 1397 | StageII | 58 |
| TCGA-B0-4693 | 1 | 77 | StageIII | 72 |  | TCGA-A3-3326 | 0 | 1137 | StageI | 47 |
| TCGA-B8-5158 | 0 | 636 | StageIII | 56 |  | TCGA-A3-3376 | 1 | 1696 | StageI | 51 |
| TCGA-BP-5183 | 0 | 1291 | StageIII | 57 |  | TCGA-AK-3444 | 0 | 1471 | StageI | 80 |
| TCGA-B0-5085 | 1 | 770 | StageIII | 76 |  | TCGA-CW-5588 | 0 | 2017 | StageI | 78 |
| TCGA-BP-4798 | 1 | 334 | StageIII | 74 |  | TCGA-CZ-5456 | 0 | 1712 | StageII | 57 |
| TCGA-B8-4151 | 0 | 838 | StageIII | 51 |  | TCGA-EU-5904 | 0 | 551 | StageI | 47 |
| TCGA-AK-3426 | 1 | 885 | StageIII | 37 |  | TCGA-EU-5905 | 0 | 119 | StageI | 67 |
| TCGA-B0-4713 | 1 | 202 | StageIII | 76 |  | TCGA-EU-5906 | 0 | 206 | StageI | 55 |
| TCGA-B0-5108 | 0 | 911 | StageIII | 54 |  | TCGA-A3-3346 | 1 | 137 | StageI | 68 |
| TCGA-CJ-4869 | 0 | 2554 | StageIII | 49 |  | TCGA-AK-3447 | 0 | 1217 | StageII | 83 |
| TCGA-B0-4696 | 1 | 866 | StageIII | 58 |  | TCGA-AK-3425 | 0 | 3343 | StageI | 68 |
| TCGA-B0-5100 | 1 | 1913 | StageIII | 72 |  | TCGA-B0-4822 | 1 | 1111 | StageII | 78 |
| TCGA-BP-5202 | 0 | 29 | StageIII | 75 |  | TCGA-BP-5185 | 0 | 1132 | StageI | 56 |
| TCGA-B8-4153 | 0 | 762 | StageIII | 74 |  | TCGA-B0-4710 | 0 | 96 | StageIII | 75 |
| TCGA-B0-4842 | 1 | 1724 | StageIII | 73 |  | TCGA-CZ-5459 | 0 | 1683 | StageIII | 63 |
| TCGA-B0-4815 | 1 | 1588 | StageIII | 65 |  | TCGA-EU-5907 | 0 | 127 | StageIII | 81 |
| TCGA-AK-3445 | 0 | 1280 | StageIII | 69 |  | TCGA-B2-A4SR | 0 | 507 | StageII | 61 |
| TCGA-CJ-4881 | 0 | 2014 | StageIII | 41 |  | TCGA-B8-A54F | 0 | 519 | StageI | 49 |
| TCGA-B0-4694 | 1 | 106 | StageIII | 72 |  | TCGA-B8-A54G | 0 | 53 | StageI | 50 |
| TCGA-CJ-4891 | 1 | 819 | StageIII | 57 |  | TCGA-MM-A564 | 0 | 607 | StageII | 68 |
| TCGA-CJ-4901 | 0 | 1450 | StageIII | 47 |  | TCGA-B8-A54D | 0 | 830 | StageIII | 69 |
| TCGA-BP-4332 | 0 | 1133 | StageIII | 36 |  | TCGA-B8-A54E | 0 | 909 | StageI | 62 |
| TCGA-B0-5116 | 0 | 657 | StageIII | 52 |  | TCGA-CJ-4642 | 0 | 1628 | StageII | 47 |
| TCGA-BP-4799 | 1 | 1133 | StageIII | 70 |  | TCGA-AK-3451 | 0 | 1481 | StageII | 48 |
| TCGA-BP-4989 | 0 | 118 | StageIII | 58 |  | TCGA-CJ-4870 | 0 | 1498 | StageIII | 58 |
| TCGA-CZ-5454 | 1 | 722 | StageIV | 63 |  | TCGA-BP-4331 | 1 | 2454 | StageI | 52 |
| TCGA-CJ-4638 | 1 | 431 | StageIV | 46 |  | TCGA-A3-A6NJ | 0 | 468 | StageI | 57 |
| TCGA-B0-5712 | 0 | 2722 | StageIV | 68 |  | TCGA-B8-A54H | 0 | 256 | StageII | 69 |
| TCGA-B0-4700 | 1 | 1980 | StageIV | 60 |  | TCGA-AK-3455 | 1 | 683 | StageIII | 71 |
| TCGA-BP-4770 | 1 | 329 | StageIV | 73 |  | TCGA-B8-A54I | 0 | 150 | StageI | 48 |
| TCGA-B0-4846 | 1 | 1200 | StageIV | 52 |  | TCGA-B8-A54J | 0 | 528 | StageII | 60 |
| TCGA-CZ-5468 | 1 | 59 | StageIV | 84 |  | TCGA-6D-AA2E | 0 | 267 | StageI | 68 |
| TCGA-B0-4847 | 1 | 793 | StageIV | 60 |  | TCGA-AK-3456 | 0 | 1143 | StageII | 48 |
| TCGA-CW-5591 | 0 | 2271 | StageIV | 56 |  | TCGA-G6-A8L7 | 0 | 1727 | StageI | 81 |
| TCGA-B8-4622 | 0 | 700 | StageIV | 57 |  | TCGA-MM-A84U | 0 | 464 | StageI | 58 |
| TCGA-B0-4697 | 1 | 578 | StageIV | 46 |  | TCGA-B8-A8YJ | 0 | 24 | StageI | 60 |
| TCGA-CJ-5681 | 1 | 552 | StageIV | 44 |  | TCGA-MM-A563 | 0 | 591 | StageI | 41 |
| TCGA-G6-A5PC | 1 | 242 | StageIV | 54 |  | TCGA-AK-3458 | 0 | 1168 | StageI | 48 |
| TCGA-CJ-4918 | 1 | 93 | StageIV | 64 |  | TCGA-AK-3460 | 0 | 951 | StageI | 58 |
| TCGA-CJ-5678 | 1 | 574 | StageIV | 62 |  | TCGA-AK-3461 | 0 | 853 | StageI | 72 |
| TCGA-CW-6087 | 1 | 41 | StageIV | 61 |  | TCGA-B0-4688 | 1 | 101 | StageIV | 46 |
| TCGA-CJ-6033 | 1 | 224 | StageIV | 54 |  | TCGA-B0-4690 | 1 | 43 | StageIV | 65 |
| TCGA-B4-5377 | 0 | 291 | StageIV | 68 |  | TCGA-A3-3316 | 0 | 1493 | StageII | 57 |
| TCGA-CZ-5987 | 1 | 445 | StageIV | 60 |  | TCGA-B0-4701 | 1 | 238 | StageIV | 66 |
| TCGA-B0-4841 | 1 | 204 | StageIV | 63 |  | TCGA-B0-4703 | 1 | 182 | StageIV | 51 |
| TCGA-B0-4836 | 1 | 1238 | StageIV | 61 |  | TCGA-B0-4707 | 1 | 600 | StageIII | 63 |
| TCGA-B0-4699 | 1 | 110 | StageIV | 74 |  | TCGA-B0-4718 | 0 | 616 | StageIII | 57 |
| TCGA-CW-5580 | 1 | 1964 | StageIV | 73 |  | TCGA-B0-4811 | 1 | 1417 | StageIII | 48 |
| TCGA-B0-5094 | 1 | 333 | StageIV | 62 |  | TCGA-B0-4814 | 1 | 168 | StageIV | 58 |
| TCGA-B0-4712 | 1 | 1337 | StageIV | 76 |  | TCGA-B0-4816 | 1 | 1371 | StageII | 49 |
| TCGA-B0-4714 | 1 | 99 | StageIV | 81 |  | TCGA-B0-4817 | 1 | 1019 | StageIII | 81 |
| TCGA-CZ-4860 | 1 | 206 | StageIV | 60 |  | TCGA-B0-4818 | 1 | 510 | StageII | 68 |
| TCGA-CJ-4890 | 0 | 2085 | StageIV | 72 |  | TCGA-B0-4819 | 1 | 183 | StageIV | 60 |
| TCGA-BP-4354 | 1 | 1034 | StageIV | 40 |  | TCGA-B0-4823 | 1 | 454 | StageI | 88 |
| TCGA-B8-4143 | 1 | 709 | StageIV | 66 |  | TCGA-B0-4828 | 1 | 307 | StageIV | 79 |
| TCGA-B0-4698 | 1 | 42 | StageIV | 75 |  | TCGA-B0-4834 | 1 | 2090 | StageI | 49 |
| TCGA-BP-5201 | 0 | 951 | StageIV | 63 |  | TCGA-B0-4844 | 1 | 313 | StageIV | 60 |
| TCGA-BP-4335 | 1 | 461 | StageIV | 65 |  | TCGA-B0-4845 | 1 | 1986 | StageIV | 70 |
| TCGA-G6-A8L6 | 0 | 305 | StageIV | 55 |  |  |  |  |  |  |
