# Supplementary material for: A Five-Gene Signature Predicts Prognosis in Patients with Kidney Renal Clear Cell Carcinoma
Source: Comput Math Methods Med. 2015 Oct 11;2015:842784. doi: 10.1155/2015/842784 (PMC4619904; doi:10.1155/2015/842784)
Supplement: Supplementary file 1 — Table S1 The sample list and information of patients in training set and testing set. Table S2 Univariable Cox regression analysis with a significance level of 0.001 reveal significant relation between gene expression and survival time. Figure S1 Kaplan-Meier curves analysis of the different clinical stages in the testing set. The two-sided log-rank test were used to determine the survival differences. Figure S2 Receiver operating characteristic (ROC) analysis of sensitivity and specificity by five-gene model in predicting survival time of patient with different clinical stages in the testing set. [file 842784.f1.zip › TableS2.docx]

**Table S2** Univariable Cox regression analysis with a significance level of 0.001 reveal significant relation between gene expression level and survival time.

| Gene name | Coefficient | Parametric P value |
| --- | --- | --- |
| A1CF | -0.124 | 0.00079 |
| A2M | -0.358 | 0.00045 |
| AAAS | 1.11 | 2.60E-06 |
| AANAT | 0.547 | 9.30E-05 |
| ABAT | -0.335 | 0.00047 |
| ABCA7 | 0.34 | 0.00019 |
| ABCA8 | -0.182 | 0.00055 |
| ABCB1 | -0.172 | 0.00032 |
| ABCB7 | -0.746 | 0.00025 |
| ABCB9 | 0.433 | 7.00E-05 |
| ABCC10 | 0.953 | 1.20E-07 |
| ABCC4 | -0.48 | 4.70E-07 |
| ABCD3 | -0.634 | 0.00067 |
| ABCG1 | -0.415 | 0.00026 |
| ABCG2 | -0.445 | 8.90E-09 |
| ABHD12 | 0.614 | 0.00049 |
| ABHD2 | -0.528 | 5.10E-05 |
| ABHD6 | -0.552 | 1.60E-06 |
| ABP1 | -0.105 | 0.00068 |
| ACAA2 | -0.58 | 1.80E-07 |
| ACADL | -0.278 | 2.20E-11 |
| ACADM | -0.714 | 8.00E-10 |
| ACADSB | -0.72 | 1.20E-09 |
| ACAT1 | -0.518 | 2.50E-06 |
| ACBD5 | -0.663 | 0.00048 |
| ACBD7 | 0.362 | 0.00019 |
| ACCN2 | 0.181 | 0.00023 |
| ACCN3 | 0.337 | 1.70E-07 |
| ACCN4 | 0.365 | 0.00027 |
| ACE2 | -0.154 | 7.90E-08 |
| ACER2 | -0.309 | 1.90E-06 |
| ACIN1 | 0.748 | 0.00032 |
| ACO2 | -0.619 | 2.00E-05 |
| ACOT4 | -0.328 | 0.00043 |
| ACOT8 | 0.476 | 0.00055 |
| ACOT9 | 1.16 | 1.60E-05 |
| ACP2 | 0.775 | 8.70E-05 |
| ACRC | 0.421 | 1.00E-05 |
| ACRV1 | 0.508 | 0.00012 |
| ACSM3 | -0.286 | 0.001 |
| ACSS3 | -0.238 | 3.20E-05 |
| ACTB | 0.91 | 1.40E-05 |
| ACTN2 | 0.175 | 0.00038 |
| ADAM10 | -0.29 | 0.00022 |
| ADAM11 | 0.413 | 6.90E-07 |
| ADAM12 | 0.216 | 7.60E-05 |
| ADAM19 | 0.349 | 0.00043 |
| ADAM20 | 0.43 | 0.00078 |
| ADAM22 | -0.261 | 1.60E-05 |
| ADAM32 | 0.253 | 0.00018 |
| ADAM33 | 0.179 | 0.00013 |
| ADAM8 | 0.453 | 1.00E-07 |
| ADAMTS13 | 0.313 | 0.00035 |
| ADAMTS14 | 0.372 | 1.90E-11 |
| ADAMTS6 | 0.324 | 7.40E-05 |
| ADAMTSL3 | -0.232 | 0.00027 |
| ADAMTSL5 | 0.256 | 0.00084 |
| ADAT2 | 0.463 | 9.60E-05 |
| ADA | 0.325 | 5.00E-05 |
| ADCK1 | 0.453 | 0.00035 |
| ADCK5 | 0.504 | 8.20E-05 |
| ADCY1 | -0.275 | 0.00048 |
| ADCY5 | -0.239 | 7.70E-09 |
| ADCY9 | -0.732 | 5.80E-07 |
| ADD1 | -1.2 | 1.60E-05 |
| ADD3 | -0.649 | 1.60E-05 |
| ADH5 | -1.04 | 2.50E-06 |
| ADORA2B | 0.36 | 0.00017 |
| ADPGK | 1.24 | 6.40E-06 |
| ADPRHL1 | 0.34 | 0.00056 |
| ADRB3 | 0.428 | 6.70E-05 |
| ADRM1 | 0.776 | 9.30E-06 |
| AFG3L1 | 0.497 | 2.10E-06 |
| AFG3L2 | -0.759 | 3.80E-06 |
| AFTPH | -0.818 | 6.00E-06 |
| AGAP3 | 0.896 | 7.20E-05 |
| AGAP4 | 0.451 | 1.40E-06 |
| AGAP6 | 0.412 | 3.50E-07 |
| AGAP7 | 0.427 | 9.80E-09 |
| AGAP8 | 0.344 | 4.40E-06 |
| AGBL2 | 0.365 | 1.90E-05 |
| AGBL4 | -0.372 | 0.00041 |
| AGER | 0.319 | 2.50E-05 |
| AGGF1 | -0.727 | 0.00063 |
| AGK | 0.904 | 0.00015 |
| AGPAT9 | -0.348 | 1.10E-05 |
| AGTR1 | -0.173 | 0.00051 |
| AGXT2 | -0.109 | 0.00025 |
| AHI1 | 0.573 | 0.00053 |
| AHSA2 | 0.346 | 2.30E-05 |
| AIDA | 0.709 | 0.00098 |
| AIF1L | -0.235 | 2.20E-05 |
| AIFM3 | 0.285 | 0.00022 |
| AIM1L | 0.258 | 3.20E-05 |
| AIM2 | 0.268 | 8.30E-06 |
| AIRE | 0.422 | 0.00055 |
| AJAP1 | -0.26 | 4.00E-06 |
| AK7 | -0.302 | 1.50E-05 |
| AKAP1 | -0.772 | 8.00E-04 |
| AKAP6 | -0.427 | 2.20E-08 |
| AKAP8L | 1 | 1.50E-07 |
| AKNAD1 | 0.502 | 9.40E-05 |
| AKR1C1 | -0.278 | 0.00025 |
| AKT1S1 | 0.692 | 0.00098 |
| AKT3 | -0.503 | 3.90E-05 |
| AKTIP | -1.29 | 1.20E-06 |
| ALDH18A1 | 0.679 | 0.00069 |
| ALDH1L1 | -0.193 | 5.10E-07 |
| ALDH1L2 | 0.283 | 0.00081 |
| ALDH3A2 | -0.659 | 4.20E-07 |
| ALDH6A1 | -0.432 | 5.20E-09 |
| ALDH7A1 | -0.742 | 0.00035 |
| ALG3 | 0.726 | 1.00E-06 |
| ALG5 | -1.26 | 4.70E-05 |
| ALKBH3 | 0.833 | 0.00073 |
| ALKBH6 | 0.578 | 9.00E-05 |
| ALPK1 | 0.868 | 5.50E-05 |
| AMAC1L2 | 0.441 | 6.10E-05 |
| AMD1 | -0.765 | 4.20E-06 |
| AMH | 0.321 | 5.80E-06 |
| AMIGO1 | -0.338 | 1.30E-05 |
| AMIGO3 | 0.613 | 1.80E-06 |
| AMOTL1 | -0.512 | 5.70E-05 |
| AMOT | -0.611 | 1.60E-09 |
| AMPD2 | 0.823 | 0.00052 |
| AMY1A | 0.318 | 6.10E-05 |
| AMY2A | 0.449 | 2.60E-05 |
| AMY2B | 0.248 | 0.00045 |
| AMZ2P1 | 0.695 | 4.00E-05 |
| ANAPC4 | 1.25 | 1.40E-06 |
| ANAPC5 | 0.987 | 5.50E-06 |
| ANAPC7 | 1.67 | 2.20E-09 |
| ANGPTL3 | -0.132 | 0.001 |
| ANGPTL6 | 0.328 | 7.10E-06 |
| ANK3 | -0.381 | 1.20E-10 |
| ANKDD1A | 0.592 | 8.70E-06 |
| ANKLE1 | 0.442 | 1.00E-08 |
| ANKRD10 | 0.702 | 3.10E-05 |
| ANKRD13B | 0.368 | 0.00024 |
| ANKRD13D | 0.851 | 3.90E-10 |
| ANKRD23 | 0.475 | 1.40E-05 |
| ANKRD33 | 0.358 | 5.70E-07 |
| ANKRD36BP1 | -0.262 | 0.00013 |
| ANKRD36 | 0.414 | 1.40E-05 |
| ANKRD46 | -0.573 | 0.00069 |
| ANKRD56 | -0.456 | 5.70E-15 |
| ANKRD57 | -0.699 | 2.20E-07 |
| ANKRD5 | -0.437 | 0.00087 |
| ANKS4B | -0.135 | 0.00037 |
| ANLN | 0.354 | 4.20E-05 |
| ANO3 | -0.243 | 6.70E-06 |
| ANO4 | -0.2 | 2.70E-05 |
| ANXA2 | 0.597 | 0.00012 |
| ANXA8L2 | 0.169 | 7.10E-05 |
| ANXA8 | 0.205 | 1.10E-06 |
| AP1AR | -0.669 | 2.20E-05 |
| AP1G2 | 0.452 | 9.40E-05 |
| AP1M1 | 0.934 | 0.00011 |
| AP2A1 | 1.05 | 9.50E-05 |
| AP2B1 | -1.13 | 0.00014 |
| AP2M1 | 0.995 | 2.20E-05 |
| AP2S1 | 0.462 | 0.00016 |
| AP3B1 | -0.897 | 1.60E-05 |
| AP4B1 | 0.954 | 0.00045 |
| APBB1 | -0.558 | 0.00021 |
| APBB3 | 0.393 | 0.00013 |
| APCDD1L | 0.192 | 5.20E-06 |
| APC | -0.51 | 8.40E-05 |
| APEX2 | 0.74 | 8.50E-05 |
| APLNR | -0.27 | 9.60E-05 |
| APLP1 | 0.213 | 0.00012 |
| APOBEC3D | 0.369 | 0.00068 |
| APOBEC3G | 0.343 | 0.00069 |
| APOBEC3H | 0.261 | 0.00067 |
| APOL2 | 0.393 | 1.00E-04 |
| APOLD1 | -0.292 | 3.80E-06 |
| APOM | -0.202 | 0.00078 |
| APOOL | -0.383 | 1.50E-06 |
| APPL1 | -0.881 | 8.00E-06 |
| APP | -1.04 | 2.70E-08 |
| AQP1 | -0.371 | 4.90E-09 |
| AQP4 | -0.158 | 0.00075 |
| AQP9 | 0.151 | 0.00066 |
| AQR | -0.624 | 0.00069 |
| ARF5 | 0.622 | 0.00055 |
| ARFGAP1 | 0.693 | 1.60E-08 |
| ARHGAP11A | 0.527 | 3.80E-05 |
| ARHGAP22 | 0.53 | 0.00013 |
| ARHGAP24 | -0.631 | 3.30E-08 |
| ARHGAP31 | -0.444 | 2.10E-05 |
| ARHGAP33 | 0.298 | 0.00016 |
| ARHGAP42 | -0.28 | 0.00018 |
| ARHGAP4 | 0.569 | 5.30E-07 |
| ARHGAP5 | -0.538 | 0.00043 |
| ARHGAP6 | -0.492 | 4.70E-05 |
| ARHGAP9 | 0.293 | 0.00083 |
| ARHGEF12 | -0.915 | 2.70E-07 |
| ARHGEF16 | -0.254 | 2.80E-06 |
| ARHGEF19 | 0.629 | 1.60E-06 |
| ARHGEF1 | 0.587 | 5.70E-05 |
| ARHGEF35 | -0.424 | 0.00024 |
| ARHGEF37 | -0.36 | 3.10E-05 |
| ARHGEF3 | -0.693 | 0.00053 |
| ARHGEF9 | -0.66 | 0.001 |
| ARID5A | 0.479 | 0.00014 |
| ARL15 | -0.389 | 4.50E-05 |
| ARL4C | 0.315 | 0.00019 |
| ARL9 | 0.3 | 0.00053 |
| ARPC1B | 0.536 | 0.00013 |
| ARPC3 | 1.05 | 8.10E-08 |
| ARPC5L | 0.752 | 4.30E-07 |
| ARPC5 | 0.965 | 2.00E-05 |
| ARPP19 | -0.863 | 9.80E-05 |
| ARRDC5 | 0.273 | 0.00041 |
| ARSG | -0.41 | 0.00079 |
| ARSI | 0.215 | 2.70E-06 |
| ARTN | 0.277 | 6.50E-06 |
| AR | -0.252 | 9.30E-08 |
| ASAH1 | -0.649 | 2.90E-06 |
| ASAH2 | -0.34 | 0.00032 |
| ASAM | 0.17 | 1.30E-05 |
| ASB6 | 0.897 | 0.00018 |
| ASB8 | -1.36 | 5.50E-05 |
| ASCC2 | 1.04 | 0.00067 |
| ASCL2 | 0.285 | 0.00046 |
| ASF1B | 0.492 | 0.00014 |
| ASGR1 | 0.248 | 6.00E-05 |
| ASH2L | -0.887 | 3.00E-04 |
| ASNS | 0.521 | 4.90E-07 |
| ASPHD2 | 0.313 | 0.00013 |
| ASPM | 0.333 | 0.00013 |
| ASPSCR1 | 0.522 | 8.30E-05 |
| ASRGL1 | -0.29 | 0.00023 |
| ASTN2 | -0.473 | 5.50E-05 |
| ASXL2 | -0.381 | 3.00E-06 |
| ASXL3 | -0.25 | 2.70E-07 |
| ATAD2 | 0.584 | 0.00019 |
| ATAD3B | 0.484 | 4.70E-07 |
| ATAD5 | 0.464 | 0.00052 |
| ATE1 | -0.434 | 7.00E-08 |
| ATF2 | -0.26 | 0.00061 |
| ATF5 | 0.746 | 7.30E-09 |
| ATF6 | -0.733 | 3.00E-05 |
| ATG16L2 | 0.31 | 0.00045 |
| ATG4B | 0.881 | 1.80E-06 |
| ATG4C | -0.517 | 0.00079 |
| ATN1 | 0.781 | 0.00087 |
| ATOH8 | -0.261 | 9.20E-05 |
| ATP10D | -0.604 | 1.00E-05 |
| ATP11A | -0.294 | 5.60E-05 |
| ATP13A1 | 0.802 | 0.00027 |
| ATP1A1 | -0.801 | 6.10E-05 |
| ATP1B3 | 0.901 | 0.00013 |
| ATP2A1 | 0.285 | 2.10E-05 |
| ATP5E | 0.429 | 7.60E-05 |
| ATP5G2 | 0.623 | 0.00054 |
| ATP6V1E2 | 0.733 | 4.40E-05 |
| ATP7A | -0.568 | 0.00075 |
| ATP7B | -0.424 | 7.20E-05 |
| ATP8A1 | -0.335 | 0.00018 |
| ATP8B5P | 0.538 | 0.00036 |
| ATRN | -0.639 | 0.00048 |
| ATXN1L | -0.563 | 3.70E-06 |
| ATXN2L | 1.3 | 1.00E-06 |
| ATXN7L2 | 0.633 | 3.30E-09 |
| ATXN7L3B | -1.09 | 0.00022 |
| ATXN7L3 | 1.62 | 7.00E-08 |
| AUH | -0.608 | 2.70E-05 |
| AUP1 | 1.03 | 4.00E-08 |
| AURKAPS1 | 0.545 | 3.20E-06 |
| AURKA | 0.502 | 1.60E-06 |
| AURKB | 0.474 | 6.30E-11 |
| AVEN | 0.709 | 2.70E-05 |
| AVIL | 0.288 | 0.00029 |
| AVPR1B | -0.241 | 6.40E-07 |
| AXIN1 | 0.873 | 0.00033 |
| AZI1 | 0.592 | 0.00076 |
| B3GAT1 | 0.254 | 0.00017 |
| B3GAT2 | 0.286 | 0.00021 |
| B3GAT3 | 0.748 | 1.90E-07 |
| B3GNTL1 | 0.743 | 2.30E-09 |
| B4GALT2 | 0.567 | 3.00E-04 |
| B4GALT7 | 0.44 | 0.00059 |
| BAG2 | 0.43 | 1.80E-06 |
| BAI1 | 0.258 | 1.50E-07 |
| BAIAP2L1 | 0.306 | 2.10E-05 |
| BAK1 | 0.815 | 7.10E-06 |
| BANF1 | 0.684 | 1.30E-06 |
| BANK1 | -0.277 | 4.10E-05 |
| BARX1 | 0.255 | 1.00E-07 |
| BASP1 | 0.312 | 6.10E-06 |
| BAT1 | 0.744 | 7.20E-05 |
| BATF | 0.295 | 4.90E-05 |
| BAZ1A | 0.626 | 0.00057 |
| BBOX1 | -0.126 | 4.40E-05 |
| BBS12 | -0.657 | 1.30E-05 |
| BBS1 | -0.542 | 3.30E-06 |
| BBS7 | -0.817 | 0.00035 |
| BCAR3 | -0.666 | 5.40E-05 |
| BCAS3 | -0.797 | 0.00014 |
| BCAT1 | 0.348 | 0.00059 |
| BCL2A1 | 0.247 | 0.00079 |
| BCL2L12 | 0.711 | 7.50E-08 |
| BCL2 | -0.554 | 1.40E-07 |
| BCL3 | 0.612 | 6.00E-08 |
| BCL6 | 0.516 | 0.00082 |
| BCL8 | 0.385 | 0.00029 |
| BCO2 | 0.214 | 0.00081 |
| BDH2 | -0.669 | 2.40E-05 |
| BEND6 | 0.296 | 2.10E-05 |
| BEST1 | 0.453 | 3.90E-05 |
| BGLAP | 0.406 | 0.00014 |
| BID | 0.941 | 7.30E-08 |
| BIRC5 | 0.499 | 1.40E-10 |
| BLM | 0.489 | 3.90E-05 |
| BLOC1S3 | 0.99 | 3.90E-05 |
| BMP1 | 0.618 | 1.50E-06 |
| BMP5 | -0.203 | 0.00041 |
| BNIP3 | -0.374 | 0.00069 |
| BNIPL | 0.435 | 7.80E-07 |
| BOLA3 | 0.534 | 8.20E-05 |
| BPHL | -0.591 | 0.00017 |
| BPNT1 | -0.948 | 5.80E-07 |
| BRAF | -0.498 | 5.00E-05 |
| BRD7P3 | 0.35 | 0.00034 |
| BRD9 | 1.07 | 2.10E-06 |
| BREA2 | 0.499 | 1.70E-05 |
| BRMS1L | -0.526 | 0.00092 |
| BRMS1 | 0.567 | 0.00091 |
| BRSK1 | 0.341 | 6.70E-05 |
| BSPRY | -0.206 | 2.00E-04 |
| BTBD11 | 0.204 | 5.90E-05 |
| BTBD12 | 0.731 | 0.00044 |
| BTBD8 | -0.345 | 1.30E-05 |
| BTBD9 | -0.781 | 0.00097 |
| BTD | -0.84 | 4.40E-05 |
| BUB1B | 0.466 | 8.30E-07 |
| BUB1 | 0.411 | 3.10E-06 |
| BUB3 | 1.15 | 0.00028 |
| BUD31 | 0.539 | 7.40E-05 |
| BZW2 | 0.43 | 0.00067 |
| C10orf108 | -0.268 | 0.00027 |
| C10orf118 | -0.406 | 1.90E-05 |
| C10orf58 | -0.438 | 4.90E-05 |
| C10orf81 | 0.155 | 0.00014 |
| C10orf90 | 0.361 | 6.50E-05 |
| C11orf24 | 0.9 | 1.80E-06 |
| C11orf45 | 0.437 | 1.20E-05 |
| C11orf48 | 0.644 | 7.80E-08 |
| C11orf52 | -0.373 | 1.60E-05 |
| C11orf54 | -0.491 | 2.00E-07 |
| C11orf61 | 0.65 | 1.10E-05 |
| C11orf70 | 0.223 | 0.00022 |
| C11orf73 | 0.972 | 8.40E-07 |
| C11orf82 | 0.518 | 1.80E-05 |
| C11orf84 | 0.914 | 9.10E-08 |
| C11orf87 | 0.36 | 0.00036 |
| C11orf92 | -0.238 | 0.00048 |
| C12orf32 | 0.899 | 1.20E-08 |
| C12orf39 | -0.189 | 1.50E-05 |
| C12orf45 | 0.962 | 8.70E-09 |
| C12orf48 | 0.422 | 0.00022 |
| C12orf49 | -0.618 | 1.80E-05 |
| C12orf65 | 0.858 | 2.50E-05 |
| C12orf70 | 0.616 | 5.30E-09 |
| C12orf72 | -1.09 | 2.70E-05 |
| C12orf73 | 0.628 | 0.00047 |
| C13orf16 | 0.345 | 2.60E-06 |
| C13orf34 | 0.983 | 5.00E-07 |
| C14orf105 | -0.359 | 2.80E-06 |
| C14orf138 | 0.682 | 3.00E-06 |
| C14orf153 | 0.624 | 0.00023 |
| C14orf156 | 0.567 | 0.00022 |
| C14orf166 | 0.733 | 0.00086 |
| C14orf176 | 0.453 | 4.00E-05 |
| C14orf181 | 0.446 | 1.60E-06 |
| C14orf182 | 0.467 | 1.10E-05 |
| C14orf184 | 0.323 | 9.00E-05 |
| C14orf21 | -0.499 | 4.70E-05 |
| C14orf34 | 0.434 | 2.80E-07 |
| C14orf80 | 0.393 | 0.00024 |
| C15orf23 | 0.861 | 3.10E-09 |
| C15orf29 | 0.882 | 3.40E-05 |
| C15orf33 | -0.377 | 1.30E-06 |
| C15orf38 | -0.764 | 1.10E-05 |
| C15orf42 | 0.579 | 1.10E-10 |
| C15orf52 | 0.352 | 0.00091 |
| C15orf5 | 0.678 | 5.90E-07 |
| C16orf57 | 1.02 | 1.50E-07 |
| C16orf59 | 0.475 | 3.00E-08 |
| C16orf62 | -1.12 | 1.80E-05 |
| C16orf68 | 0.744 | 9.10E-06 |
| C16orf79 | 0.296 | 3.60E-05 |
| C16orf93 | 0.362 | 0.00017 |
| C17orf37 | 0.428 | 0.00047 |
| C17orf47 | 0.412 | 4.40E-07 |
| C17orf53 | 0.597 | 6.70E-06 |
| C17orf56 | 0.65 | 1.10E-07 |
| C17orf58 | 0.647 | 0.00021 |
| C17orf60 | 0.39 | 0.00021 |
| C17orf62 | 1.02 | 1.80E-07 |
| C17orf64 | 0.47 | 6.60E-06 |
| C17orf65 | 0.842 | 1.40E-06 |
| C17orf66 | 0.387 | 1.20E-05 |
| C17orf67 | 0.57 | 2.20E-05 |
| C17orf70 | 0.824 | 1.30E-05 |
| C17orf71 | -0.612 | 3.50E-05 |
| C17orf89 | 0.357 | 0.00058 |
| C17orf90 | 0.494 | 2.00E-04 |
| C17orf95 | 1.03 | 6.70E-05 |
| C17orf96 | 0.507 | 6.30E-07 |
| C19orf10 | 0.592 | 0.00084 |
| C19orf22 | 0.655 | 1.80E-05 |
| C19orf36 | 0.306 | 8.60E-06 |
| C19orf48 | 0.458 | 0.00013 |
| C19orf50 | 1.32 | 9.20E-07 |
| C19orf53 | 0.549 | 3.50E-05 |
| C19orf54 | 0.879 | 1.80E-05 |
| C19orf57 | 0.431 | 0.00067 |
| C19orf60 | 0.375 | 0.00043 |
| C19orf61 | 0.896 | 9.10E-08 |
| C19orf66 | 0.812 | 1.40E-06 |
| C19orf73 | 0.358 | 0.00037 |
| C19orf76 | 0.487 | 9.40E-08 |
| C19orf77 | -0.289 | 7.50E-11 |
| C1QTNF1 | 0.264 | 0.00072 |
| C1QTNF6 | 0.583 | 2.80E-08 |
| C1RL | 0.694 | 6.60E-06 |
| C1R | 0.301 | 2.90E-05 |
| C1S | 0.28 | 6.00E-05 |
| C1orf104 | 0.336 | 0.00018 |
| C1orf115 | -0.325 | 4.80E-05 |
| C1orf122 | 0.486 | 0.00016 |
| C1orf131 | 1.16 | 4.00E-05 |
| C1orf135 | 0.551 | 1.30E-06 |
| C1orf150 | -0.358 | 0.00013 |
| C1orf159 | 0.598 | 6.30E-06 |
| C1orf174 | 1.43 | 8.70E-06 |
| C1orf182 | 0.432 | 0.00075 |
| C1orf190 | -0.291 | 0.00068 |
| C1orf192 | -0.389 | 6.70E-06 |
| C1orf204 | 0.39 | 0.00075 |
| C1orf210 | -0.251 | 4.60E-08 |
| C1orf213 | 0.358 | 7.10E-05 |
| C1orf216 | 1.06 | 7.00E-06 |
| C1orf220 | 0.276 | 0.00094 |
| C1orf228 | 0.472 | 1.40E-09 |
| C1orf25 | -0.79 | 9.40E-05 |
| C1orf26 | -0.512 | 0.00031 |
| C1orf52 | 1.24 | 2.10E-06 |
| C1orf58 | -0.353 | 3.40E-05 |
| C1orf61 | 0.357 | 2.70E-05 |
| C1orf63 | 0.318 | 0.00097 |
| C1orf69 | -0.693 | 9.30E-06 |
| C1orf77 | 1.22 | 0.00036 |
| C1orf86 | 0.41 | 0.00048 |
| C1orf88 | -0.309 | 0.00073 |
| C1orf89 | -0.562 | 9.30E-06 |
| C1orf91 | 0.765 | 3.90E-06 |
| C1orf92 | 0.322 | 0.00019 |
| C1orf96 | 0.782 | 1.30E-07 |
| C20orf103 | 0.2 | 0.00022 |
| C20orf106 | 0.437 | 0.00041 |
| C20orf107 | 0.398 | 0.00083 |
| C20orf118 | 0.35 | 2.00E-04 |
| C20orf20 | 0.988 | 2.20E-08 |
| C20orf24 | 0.469 | 0.00055 |
| C20orf27 | 0.596 | 0.00085 |
| C20orf72 | 1.1 | 4.90E-05 |
| C21orf125 | 0.422 | 8.50E-08 |
| C21orf15 | 0.314 | 3.30E-06 |
| C21orf58 | 0.469 | 1.40E-06 |
| C21orf62 | -0.158 | 0.00052 |
| C21orf81 | 0.504 | 0.00011 |
| C21orf84 | 0.437 | 5.40E-07 |
| C22orf9 | 0.742 | 6.50E-06 |
| C2CD2L | 0.993 | 5.80E-05 |
| C2CD4B | 0.245 | 0.00017 |
| C2orf27A | 0.32 | 8.70E-05 |
| C2orf40 | -0.229 | 3.10E-05 |
| C2orf47 | -0.872 | 8.90E-05 |
| C2orf48 | 0.359 | 0.00034 |
| C2orf55 | -0.427 | 0.00022 |
| C2orf58 | 0.296 | 8.30E-05 |
| C2orf66 | 0.486 | 0.00014 |
| C2orf68 | 1.33 | 1.90E-07 |
| C2orf79 | 0.437 | 0.00091 |
| C2orf85 | 0.271 | 0.00061 |
| C2 | 0.218 | 0.00033 |
| C3orf21 | 0.738 | 4.20E-05 |
| C3orf35 | 0.413 | 2.40E-06 |
| C3orf36 | -0.269 | 0.00025 |
| C3orf47 | 0.369 | 0.00043 |
| C3orf55 | 0.248 | 1.40E-05 |
| C3orf62 | 0.576 | 0.00078 |
| C4orf12 | -0.427 | 2.60E-07 |
| C4orf19 | -0.359 | 9.50E-08 |
| C4orf21 | 0.595 | 7.40E-06 |
| C4orf38 | -0.317 | 0.00035 |
| C4orf39 | 0.35 | 0.00027 |
| C4orf3 | -1.01 | 0.00012 |
| C4orf48 | 0.175 | 8.00E-04 |
| C5orf23 | -0.279 | 1.80E-08 |
| C5orf41 | -0.612 | 7.10E-08 |
| C5orf4 | -0.404 | 3.90E-05 |
| C6orf115 | 0.496 | 0.00091 |
| C6orf122 | 0.424 | 3.60E-08 |
| C6orf125 | 0.533 | 1.30E-06 |
| C6orf129 | 0.444 | 0.00015 |
| C6orf130 | -0.906 | 0.00088 |
| C6orf134 | 0.625 | 3.10E-05 |
| C6orf141 | 0.218 | 2.50E-05 |
| C6orf147 | 0.382 | 5.30E-06 |
| C6orf153 | 0.794 | 2.50E-06 |
| C6orf163 | 0.488 | 2.40E-06 |
| C6orf186 | -0.374 | 8.40E-08 |
| C6orf195 | -0.431 | 0.00056 |
| C6orf1 | 0.567 | 4.90E-07 |
| C6orf26 | 0.362 | 0.00023 |
| C6orf27 | -0.261 | 0.00011 |
| C6orf59 | 0.315 | 0.00044 |
| C6orf72 | -0.548 | 0.00015 |
| C6orf81 | 0.497 | 1.20E-12 |
| C6orf89 | -1.16 | 0.00031 |
| C7orf10 | -0.228 | 0.00099 |
| C7orf29 | 0.517 | 7.10E-06 |
| C7orf41 | -0.441 | 0.00041 |
| C7orf43 | 0.797 | 3.20E-06 |
| C7orf47 | 0.605 | 3.40E-06 |
| C7orf51 | 0.337 | 0.00048 |
| C7orf53 | 0.422 | 3.20E-07 |
| C7orf54 | 0.592 | 8.80E-07 |
| C7orf61 | 0.496 | 1.00E-07 |
| C8ORFK29 | 0.384 | 1.10E-06 |
| C8orf30A | 0.691 | 9.50E-06 |
| C8orf31 | 0.207 | 0.00093 |
| C8orf37 | -0.547 | 0.00012 |
| C8orf44 | 0.739 | 2.90E-05 |
| C8orf47 | -0.177 | 2.60E-05 |
| C8orf59 | 0.733 | 1.20E-05 |
| C8orf76 | 0.965 | 4.00E-06 |
| C9orf100 | 0.502 | 3.40E-05 |
| C9orf125 | -0.249 | 1.30E-05 |
| C9orf129 | -0.289 | 5.10E-06 |
| C9orf140 | 0.44 | 1.80E-06 |
| C9orf142 | 0.505 | 4.40E-06 |
| C9orf16 | 0.375 | 1.00E-04 |
| C9orf30 | 0.902 | 1.50E-05 |
| C9orf37 | 0.538 | 3.20E-05 |
| C9orf41 | -0.317 | 2.80E-05 |
| C9orf5 | -0.751 | 1.40E-06 |
| C9orf66 | -0.25 | 5.70E-05 |
| C9orf69 | 0.829 | 0.00075 |
| C9orf71 | -0.153 | 0.00013 |
| C9orf80 | -0.463 | 7.40E-05 |
| C9orf91 | 0.666 | 0.00018 |
| C9orf98 | -0.378 | 0.00033 |
| CA2 | -0.353 | 5.70E-05 |
| CA4 | -0.18 | 4.10E-05 |
| CA5B | -0.787 | 0.00037 |
| CA8 | -0.253 | 0.00047 |
| CAB39L | -0.597 | 8.70E-05 |
| CABLES1 | -0.32 | 0.00016 |
| CABLES2 | 0.959 | 2.00E-08 |
| CABP7 | 0.556 | 5.40E-08 |
| CABYR | 0.543 | 1.20E-07 |
| CACNA1G | 0.275 | 2.30E-07 |
| CACNB1 | 0.644 | 3.80E-09 |
| CACNB3 | 0.555 | 1.80E-06 |
| CADPS2 | -0.654 | 1.40E-06 |
| CALB2 | 0.21 | 0.00096 |
| CALCRL | -0.344 | 2.80E-05 |
| CALML6 | 0.418 | 0.00025 |
| CALR | 0.761 | 1.90E-05 |
| CAMK1D | -0.41 | 0.00027 |
| CAMK2N2 | 0.274 | 3.50E-05 |
| CANX | -0.599 | 0.00031 |
| CAPN3 | 0.289 | 3.10E-05 |
| CAPRIN2 | 0.71 | 1.50E-06 |
| CAPZA1 | 1.2 | 6.00E-08 |
| CAPZB | 0.94 | 0.00015 |
| CARD14 | 0.199 | 0.00022 |
| CARM1 | 1.11 | 1.70E-05 |
| CARNS1 | 0.376 | 5.50E-05 |
| CARS | 1.21 | 1.80E-08 |
| CASC1 | -0.264 | 0.00036 |
| CASKIN2 | -0.454 | 7.10E-05 |
| CASP3 | 1.22 | 1.20E-08 |
| CASP4 | 0.826 | 3.40E-06 |
| CASP5 | 0.286 | 0.00027 |
| CASP9 | 1.17 | 2.00E-07 |
| CATSPER1 | 0.276 | 0.00087 |
| CATSPER2 | 0.419 | 0.00012 |
| CATSPERB | 0.302 | 5.40E-06 |
| CAT | -0.546 | 1.60E-06 |
| CBARA1 | -0.782 | 0.00059 |
| CBFB | 1.03 | 1.70E-05 |
| CBWD1 | -0.63 | 0.00012 |
| CBX3 | 0.808 | 0.00037 |
| CBX4 | 1.03 | 2.10E-06 |
| CBX6 | -0.43 | 0.00022 |
| CBX7 | -0.425 | 1.30E-05 |
| CBX8 | 0.677 | 6.50E-05 |
| CC2D1B | 1.03 | 9.60E-05 |
| CCAR1 | 1.05 | 4.00E-04 |
| CCDC109B | 0.403 | 2.00E-04 |
| CCDC114 | 0.304 | 0.00018 |
| CCDC121 | -0.966 | 2.80E-08 |
| CCDC130 | 0.701 | 1.30E-05 |
| CCDC136 | 0.373 | 9.30E-07 |
| CCDC137 | 0.855 | 9.10E-09 |
| CCDC142 | 0.558 | 4.00E-04 |
| CCDC14 | 0.634 | 2.10E-07 |
| CCDC154 | 0.315 | 1.30E-06 |
| CCDC17 | 0.358 | 1.30E-05 |
| CCDC18 | 0.522 | 9.50E-06 |
| CCDC19 | 0.483 | 4.50E-08 |
| CCDC25 | -0.861 | 4.00E-04 |
| CCDC28B | 0.349 | 2.50E-05 |
| CCDC34 | 0.454 | 0.00036 |
| CCDC41 | 0.746 | 3.50E-06 |
| CCDC42B | 0.383 | 4.40E-06 |
| CCDC47 | -0.748 | 0.00014 |
| CCDC57 | 0.416 | 6.70E-05 |
| CCDC62 | 0.419 | 0.00094 |
| CCDC74A | 0.348 | 0.00061 |
| CCDC76 | 0.629 | 0.00027 |
| CCDC78 | 0.205 | 0.00012 |
| CCDC84 | 0.531 | 3.80E-07 |
| CCDC85A | -0.354 | 8.20E-05 |
| CCDC87 | -0.464 | 0.00074 |
| CCDC88B | 0.325 | 0.00015 |
| CCDC90A | 0.913 | 0.00011 |
| CCL25 | 0.349 | 0.00027 |
| CCL26 | 0.218 | 0.00059 |
| CCL7 | 0.294 | 2.90E-05 |
| CCL8 | 0.317 | 3.60E-05 |
| CCM2 | 0.879 | 3.20E-07 |
| CCNA1 | 0.194 | 4.50E-05 |
| CCNA2 | 0.743 | 5.20E-11 |
| CCNB1 | 0.472 | 7.20E-06 |
| CCNB2 | 0.514 | 9.90E-09 |
| CCNDBP1 | -0.918 | 1.20E-06 |
| CCNE1 | 0.356 | 4.50E-08 |
| CCNE2 | 0.451 | 0.00082 |
| CCNF | 0.813 | 1.80E-11 |
| CCNG1 | -0.47 | 4.30E-05 |
| CCNH | 0.881 | 0.00049 |
| CCNI | -0.461 | 4.10E-07 |
| CCNL1 | 0.503 | 0.00013 |
| CCNL2 | 0.412 | 1.40E-06 |
| CCNT1 | -0.229 | 0.00022 |
| CCNYL1 | -0.514 | 0.00057 |
| CCR10 | 0.299 | 0.00067 |
| CD1E | -0.259 | 7.30E-05 |
| CD207 | -0.264 | 0.00023 |
| CD24 | -0.285 | 0.00073 |
| CD34 | -0.336 | 2.00E-04 |
| CD44 | 0.409 | 2.70E-05 |
| CD46 | -0.634 | 0.00038 |
| CD72 | 0.322 | 2.80E-05 |
| CD7 | 0.283 | 3.20E-05 |
| CDADC1 | -1.09 | 3.40E-07 |
| CDAN1 | 0.838 | 0.00062 |
| CDC123 | 1.32 | 8.60E-05 |
| CDC14B | -0.682 | 4.90E-07 |
| CDC14C | -0.468 | 3.20E-06 |
| CDC16 | 1.07 | 0.00037 |
| CDC20 | 0.522 | 1.60E-10 |
| CDC25A | 0.486 | 1.00E-05 |
| CDC25B | 0.679 | 2.20E-11 |
| CDC25C | 0.382 | 1.40E-06 |
| CDC42BPG | -0.333 | 1.60E-07 |
| CDC42SE2 | -0.579 | 0.00015 |
| CDC45 | 0.377 | 6.50E-05 |
| CDC6 | 0.378 | 0.00074 |
| CDC73 | -0.639 | 0.00064 |
| CDC7 | 0.692 | 4.80E-09 |
| CDCA3 | 0.57 | 2.10E-12 |
| CDCA4 | 0.745 | 4.80E-08 |
| CDCA5 | 0.531 | 8.80E-07 |
| CDCA7 | 0.438 | 2.90E-07 |
| CDCA8 | 0.568 | 6.60E-09 |
| CDH12 | 0.214 | 0.00071 |
| CDH13 | -0.274 | 0.00049 |
| CDH16 | -0.237 | 3.50E-07 |
| CDH1 | -0.278 | 7.80E-05 |
| CDHR4 | 0.365 | 1.50E-06 |
| CDK11A | 0.548 | 9.00E-04 |
| CDK16 | 0.694 | 5.50E-05 |
| CDK1 | 0.567 | 6.90E-07 |
| CDK20 | -0.504 | 0.00049 |
| CDK2AP1 | 0.777 | 5.90E-05 |
| CDK2 | 0.869 | 5.40E-05 |
| CDK3 | 0.376 | 4.40E-05 |
| CDK4 | 0.704 | 0.00016 |
| CDK5RAP1 | 0.956 | 9.30E-07 |
| CDK5RAP3 | 0.646 | 3.50E-06 |
| CDK9 | 1.13 | 9.00E-08 |
| CDKL1 | -0.335 | 2.20E-06 |
| CDKL2 | -0.396 | 1.00E-05 |
| CDKL5 | -0.269 | 7.10E-06 |
| CDKN2D | 0.729 | 0.00029 |
| CDKN3 | 0.459 | 3.30E-08 |
| CDRT15P | 0.396 | 0.00011 |
| CDRT4 | 0.531 | 0.00034 |
| CDS1 | -0.517 | 1.20E-07 |
| CDYL2 | -0.399 | 1.60E-06 |
| CEACAM19 | 0.36 | 5.40E-05 |
| CEACAM22P | 0.395 | 1.50E-06 |
| CEACAM4 | 0.274 | 0.00014 |
| CEBPB | 0.448 | 2.80E-07 |
| CEBPG | 0.741 | 0.00091 |
| CELF6 | 0.278 | 9.70E-05 |
| CELSR3 | 0.331 | 1.40E-05 |
| CENPA | 0.511 | 5.60E-11 |
| CENPBD1 | -1.22 | 1.20E-07 |
| CENPE | 0.445 | 1.20E-06 |
| CENPF | 0.46 | 3.50E-06 |
| CENPH | 0.691 | 7.00E-06 |
| CENPK | 0.405 | 0.00057 |
| CENPM | 0.473 | 5.50E-06 |
| CENPN | 0.66 | 4.00E-05 |
| CENPP | 0.706 | 1.00E-07 |
| CENPT | 0.558 | 4.00E-06 |
| CENPW | 0.743 | 2.80E-12 |
| CEP164 | 0.93 | 6.30E-06 |
| CEP55 | 0.551 | 5.60E-09 |
| CEP68 | -0.773 | 1.10E-05 |
| CERCAM | 0.511 | 1.10E-07 |
| CES2 | -0.387 | 8.00E-04 |
| CFHR3 | 0.237 | 2.40E-05 |
| CFH | 0.23 | 7.00E-04 |
| CFL1 | 0.748 | 0.00076 |
| CG030 | 0.371 | 4.30E-05 |
| CGB7 | 0.333 | 6.50E-05 |
| CGN | -0.249 | 2.10E-05 |
| CHAC1 | 0.349 | 7.30E-08 |
| CHD9 | -0.424 | 0.00024 |
| CHDH | -0.336 | 4.30E-08 |
| CHEK2 | 0.846 | 1.60E-08 |
| CHFR | 1.16 | 2.30E-10 |
| CHGA | 0.251 | 8.80E-06 |
| CHI3L2 | 0.189 | 0.00022 |
| CHKA | 0.495 | 0.00012 |
| CHKB-CPT1B | 0.291 | 6.90E-05 |
| CHMP4C | -0.377 | 1.00E-04 |
| CHMP5 | -0.717 | 0.00033 |
| CHM | -0.453 | 2.00E-04 |
| CHN1 | 0.496 | 2.30E-06 |
| CHORDC1 | 0.603 | 2.00E-04 |
| CHPF2 | 0.942 | 5.90E-06 |
| CHRD | 0.292 | 0.00088 |
| CHRM3 | -0.324 | 2.50E-06 |
| CHRNA5 | 0.315 | 7.30E-05 |
| CHST11 | 0.49 | 0.00018 |
| CHST12 | 0.799 | 0.00019 |
| CHST9 | -0.125 | 0.00087 |
| CHTF18 | 0.543 | 1.60E-08 |
| CHTF8 | -1.1 | 1.70E-07 |
| CHURC1 | -0.731 | 0.00051 |
| CIB1 | 0.533 | 0.00027 |
| CIDECP | 0.573 | 2.90E-05 |
| CILP2 | 0.213 | 8.70E-07 |
| CILP | 0.301 | 1.10E-08 |
| CKAP2L | 0.36 | 0.00037 |
| CKAP4 | 0.778 | 1.80E-09 |
| CKS2 | 0.462 | 2.50E-06 |
| CLASP2 | -0.773 | 0.00017 |
| CLCN3 | -0.428 | 0.00012 |
| CLCN5 | -0.495 | 3.90E-08 |
| CLCN7 | 0.642 | 6.30E-05 |
| CLDN10 | -0.201 | 1.90E-06 |
| CLDN15 | 0.434 | 0.00061 |
| CLDN4 | -0.368 | 1.30E-05 |
| CLDND2 | 0.363 | 4.10E-05 |
| CLEC14A | -0.312 | 0.00017 |
| CLEC2B | 0.401 | 8.60E-05 |
| CLEC2D | 0.501 | 6.10E-05 |
| CLEC3B | -0.262 | 0.00019 |
| CLEC4GP1 | 0.29 | 9.70E-06 |
| CLEC4G | 0.292 | 1.30E-05 |
| CLIC5 | -0.306 | 2.40E-05 |
| CLIP3 | 0.377 | 0.00052 |
| CLIP4 | 0.284 | 0.00086 |
| CLK2P | 0.53 | 1.00E-05 |
| CLK2 | 0.776 | 1.10E-07 |
| CLK3 | 0.777 | 0.00052 |
| CLN5 | -0.986 | 7.70E-06 |
| CLTB | 0.52 | 0.00035 |
| CLUAP1 | -0.809 | 0.00031 |
| CLVS1 | 0.269 | 1.50E-06 |
| CMAS | 0.946 | 0.00013 |
| CMBL | -0.369 | 2.20E-05 |
| CMTM3 | 0.46 | 0.00013 |
| CMTM7 | 0.557 | 1.10E-05 |
| CNDP2 | -0.367 | 0.00035 |
| CNGA1 | -0.259 | 0.00033 |
| CNIH2 | 0.469 | 8.00E-08 |
| CNN2 | 0.433 | 0.00064 |
| CNN3 | -0.798 | 0.00016 |
| CNNM2 | -0.609 | 0.00051 |
| CNNM3 | -0.702 | 0.00076 |
| CNOT1 | -0.863 | 3.50E-05 |
| CNOT3 | 0.975 | 0.00018 |
| CNOT6L | -0.769 | 1.60E-05 |
| CNPY3 | 0.917 | 1.00E-07 |
| CNP | 0.719 | 7.00E-04 |
| CNTD1 | 0.384 | 9.00E-07 |
| CNTD2 | 0.311 | 7.50E-06 |
| CNTNAP1 | 0.58 | 1.20E-07 |
| CNTNAP5 | -0.208 | 8.20E-05 |
| COBLL1 | -0.626 | 7.80E-05 |
| COBL | -0.299 | 1.00E-08 |
| COL10A1 | 0.158 | 0.00091 |
| COL11A1 | 0.16 | 6.30E-06 |
| COL11A2 | 0.244 | 0.00072 |
| COL1A1 | 0.219 | 0.00024 |
| COL22A1 | 0.164 | 0.00017 |
| COL24A1 | 0.246 | 0.00015 |
| COL25A1 | -0.199 | 0.00044 |
| COL4A3BP | -0.774 | 1.40E-05 |
| COL4A3 | -0.235 | 0.001 |
| COL4A4 | -0.388 | 9.70E-09 |
| COL5A1 | 0.281 | 4.20E-05 |
| COL6A1 | 0.541 | 2.40E-07 |
| COL6A2 | 0.398 | 6.80E-05 |
| COL6A3 | 0.257 | 0.00013 |
| COL6A4P2 | 0.351 | 2.80E-07 |
| COL7A1 | 0.277 | 1.90E-11 |
| COL8A2 | 0.265 | 5.00E-05 |
| COLQ | 0.482 | 2.50E-07 |
| COMMD5 | 0.703 | 0.00065 |
| COPG2 | 0.638 | 6.30E-05 |
| COPS4 | -0.959 | 4.30E-06 |
| COPS7B | 1.4 | 7.00E-11 |
| COPZ2 | 0.351 | 0.00062 |
| COQ7 | -1.31 | 3.80E-05 |
| CORO1B | 0.756 | 0.00053 |
| CORO6 | 0.307 | 3.10E-08 |
| COX15 | -1.11 | 2.50E-05 |
| COX19 | 0.962 | 6.40E-07 |
| COX5B | 0.396 | 0.00067 |
| COX6B2 | 0.28 | 6.30E-05 |
| CPA4 | 0.125 | 0.00033 |
| CPNE1 | 0.789 | 1.20E-05 |
| CPNE3 | -0.879 | 5.10E-06 |
| CPNE7 | 0.23 | 4.90E-06 |
| CPOX | 0.886 | 1.90E-07 |
| CPPED1 | -0.998 | 5.00E-05 |
| CPSF1 | 0.733 | 3.30E-05 |
| CPSF3 | 0.827 | 0.00046 |
| CPT1A | -0.61 | 1.50E-06 |
| CPT2 | -0.838 | 2.00E-08 |
| CRABP2 | 0.197 | 1.10E-05 |
| CRADD | -0.649 | 9.90E-06 |
| CRAT | -0.46 | 0.00093 |
| CRB3 | -0.415 | 2.50E-10 |
| CRBN | -0.767 | 0.00097 |
| CREB3L1 | 0.177 | 0.00077 |
| CREBL2 | -0.815 | 6.50E-05 |
| CRELD2 | 0.598 | 0.00015 |
| CRHBP | -0.238 | 0.00081 |
| CRIM1 | -0.607 | 5.90E-07 |
| CRP | 0.11 | 0.00069 |
| CRTC2 | 1.17 | 3.90E-05 |
| CRY2 | -0.745 | 2.00E-07 |
| CRYGS | 0.472 | 3.40E-09 |
| CRYL1 | -0.491 | 2.50E-08 |
| CRYZ | -0.338 | 0.00058 |
| CSAD | 0.343 | 1.60E-05 |
| CSAG1 | 0.217 | 0.00061 |
| CSAG3 | 0.214 | 0.00055 |
| CSDA | 0.652 | 6.20E-05 |
| CSDE1 | -0.965 | 1.30E-05 |
| CSK | 0.634 | 0.001 |
| CSNK1D | 0.69 | 0.00011 |
| CSNK1E | 0.672 | 0.00013 |
| CSNK2A1P | -0.302 | 0.00081 |
| CSRNP2 | 0.952 | 8.00E-04 |
| CST2 | 0.271 | 0.00015 |
| CSTF3 | 1.68 | 3.70E-07 |
| CTBP1 | 0.969 | 0.00012 |
| CTBP2 | -0.98 | 4.00E-05 |
| CTDSPL | -0.866 | 9.00E-06 |
| CTLA4 | 0.21 | 0.00089 |
| CTNNA1 | -0.966 | 3.30E-06 |
| CTNNB1 | -0.826 | 1.00E-06 |
| CTNND1 | -0.589 | 4.40E-05 |
| CTR9 | -0.716 | 0.00081 |
| CTRL | 0.353 | 5.30E-06 |
| CTSO | -0.632 | 5.80E-05 |
| CTTNBP2 | -0.309 | 0.00047 |
| CTU1 | 0.48 | 0.00034 |
| CTU2 | 0.545 | 0.00091 |
| CUBN | -0.157 | 1.60E-06 |
| CUL5 | -0.727 | 0.00094 |
| CX3CL1 | -0.544 | 1.20E-07 |
| CXCL13 | 0.175 | 2.90E-05 |
| CXCL2 | 0.222 | 1.00E-04 |
| CXCL3 | 0.224 | 0.00034 |
| CXCL5 | 0.122 | 2.00E-04 |
| CXorf1 | 0.267 | 0.00095 |
| CXorf26 | 0.868 | 0.00028 |
| CXorf48 | 0.508 | 3.00E-06 |
| CXorf56 | -0.26 | 0.00048 |
| CYB5A | -0.44 | 4.30E-06 |
| CYB5D2 | -0.777 | 0.00054 |
| CYBA | 0.402 | 0.00038 |
| CYFIP1 | -1.07 | 2.30E-05 |
| CYFIP2 | -0.469 | 7.20E-10 |
| CYP19A1 | 0.261 | 0.00019 |
| CYP26A1 | 0.212 | 0.00059 |
| CYP2D7P1 | 0.267 | 7.30E-06 |
| CYP2R1 | 0.859 | 7.30E-05 |
| CYP3A7 | -0.27 | 2.80E-07 |
| CYP4A11 | -0.128 | 4.20E-06 |
| CYP7B1 | -0.441 | 6.40E-06 |
| CYS1 | -0.157 | 3.20E-05 |
| CYSLTR1 | -0.331 | 6.70E-05 |
| CYYR1 | -0.37 | 2.10E-05 |
| DAGLB | 0.823 | 0.00084 |
| DAND5 | 0.301 | 0.00027 |
| DAZAP1 | 1.06 | 6.00E-07 |
| DAZAP2 | -1.28 | 7.00E-05 |
| DBF4B | 0.595 | 5.20E-05 |
| DBF4 | 0.494 | 2.80E-06 |
| DBI | 0.77 | 1.20E-05 |
| DBT | -1.11 | 3.50E-06 |
| DCAF15 | 0.978 | 1.70E-05 |
| DCAF4L1 | 0.454 | 7.10E-06 |
| DCAF7 | -0.73 | 0.00046 |
| DCBLD1 | 0.836 | 4.30E-05 |
| DCBLD2 | 0.362 | 2.60E-05 |
| DCDC2B | 0.485 | 0.00082 |
| DCLRE1C | 0.653 | 8.80E-05 |
| DCST2 | 0.462 | 2.80E-08 |
| DCTN3 | -0.621 | 0.00089 |
| DCTN6 | -0.648 | 0.00029 |
| DCTPP1 | -0.748 | 0.00016 |
| DCUN1D2 | 0.596 | 0.00061 |
| DCUN1D3 | 0.554 | 0.00076 |
| DCUN1D5 | 0.698 | 0.00053 |
| DDAH1 | -0.541 | 1.70E-07 |
| DDAH2 | 0.543 | 0.00056 |
| DDB1 | -0.921 | 0.00072 |
| DDC | -0.125 | 2.90E-05 |
| DDHD1 | 0.807 | 1.90E-05 |
| DDI2 | -0.29 | 1.00E-05 |
| DDIT3 | 0.334 | 1.00E-04 |
| DDR1 | -0.485 | 2.70E-05 |
| DDX11 | 0.519 | 1.70E-05 |
| DDX12 | 0.347 | 1.20E-05 |
| DDX1 | -1.15 | 0.00046 |
| DDX26B | 0.527 | 7.40E-05 |
| DDX39 | 0.961 | 1.60E-07 |
| DDX46 | -0.641 | 0.00065 |
| DDX55 | 0.996 | 1.60E-07 |
| DDX56 | 1.18 | 2.70E-07 |
| DEF6 | 0.331 | 0.00053 |
| DENND2C | -0.42 | 4.10E-08 |
| DENND4B | 0.811 | 1.50E-05 |
| DENND4C | -0.812 | 2.50E-06 |
| DENR | 1.03 | 0.00034 |
| DEPDC1B | 0.334 | 0.00025 |
| DEPDC1 | 0.437 | 1.40E-06 |
| DEPDC6 | -0.589 | 1.20E-05 |
| DERL2 | 0.825 | 0.00018 |
| DERL3 | 0.267 | 7.90E-05 |
| DFFA | -1.02 | 0.00049 |
| DFNB31 | 0.432 | 1.50E-05 |
| DFNB59 | 0.444 | 8.80E-05 |
| DGAT1 | 0.678 | 2.00E-04 |
| DGCR8 | 1.01 | 0.00041 |
| DGKH | -0.346 | 3.90E-05 |
| DGKI | -0.257 | 4.00E-04 |
| DGUOK | 0.923 | 6.30E-07 |
| DHH | -0.374 | 1.00E-04 |
| DHRS12 | -0.69 | 0.00011 |
| DHTKD1 | -0.413 | 0.00049 |
| DHX34 | 0.6 | 0.00052 |
| DHX58 | 0.673 | 9.60E-05 |
| DHX9 | -0.66 | 6.60E-05 |
| DIAPH1 | -0.796 | 5.20E-06 |
| DIP2C | -0.682 | 4.30E-06 |
| DISC2 | 0.668 | 3.70E-06 |
| DISP1 | -0.507 | 0.00033 |
| DKFZP434L187 | 0.29 | 9.10E-05 |
| DKFZP686I15217 | 0.538 | 3.00E-04 |
| DKFZp434J0226 | 0.292 | 1.40E-05 |
| DKFZp686O24166 | -0.269 | 8.50E-06 |
| DLAT | -0.849 | 9.20E-05 |
| DLEU2 | 0.833 | 7.30E-07 |
| DLG4 | 0.49 | 0.00039 |
| DLG5 | 0.567 | 2.00E-05 |
| DLGAP3 | 0.447 | 4.30E-05 |
| DLGAP4 | 1.26 | 1.50E-06 |
| DLGAP5 | 0.362 | 1.70E-05 |
| DLX4 | 0.36 | 1.40E-06 |
| DMGDH | -0.269 | 1.10E-07 |
| DMPK | 0.416 | 0.00032 |
| DMRT3 | 0.271 | 4.30E-08 |
| DMRTA1 | -0.439 | 1.10E-10 |
| DMTF1 | 0.485 | 0.00035 |
| DMXL1 | -0.554 | 1.00E-05 |
| DNA2 | 0.44 | 1.30E-06 |
| DNAH12 | 0.347 | 7.80E-05 |
| DNAH14 | 0.502 | 6.60E-05 |
| DNAH17 | 0.451 | 2.50E-09 |
| DNAJA2 | -1.37 | 0.00069 |
| DNAJB11 | 0.934 | 1.50E-05 |
| DNAJB14 | -0.656 | 6.50E-06 |
| DNAJB7 | 0.433 | 0.00095 |
| DNAJB9 | -0.43 | 0.00046 |
| DNAJC12 | 0.274 | 7.40E-05 |
| DNAJC24 | 0.863 | 0.00031 |
| DNAJC2 | 1.15 | 1.20E-08 |
| DNAJC3 | -0.689 | 0.00039 |
| DNAJC9 | 1.05 | 7.10E-06 |
| DNAL1 | -0.924 | 0.00046 |
| DNASE1L2 | 0.279 | 0.00025 |
| DNASE1L3 | -0.397 | 9.70E-10 |
| DNHD1 | 0.361 | 2.30E-05 |
| DNM1P35 | 0.488 | 3.20E-05 |
| DNMT3A | 0.747 | 0.00039 |
| DNMT3B | 0.35 | 0.00039 |
| DNTTIP1 | 0.539 | 1.70E-06 |
| DOCK8 | -0.323 | 0.00058 |
| DOCK9 | -0.429 | 8.70E-05 |
| DOK1 | 0.795 | 3.80E-06 |
| DOK3 | 0.477 | 1.20E-05 |
| DOLPP1 | 0.639 | 5.00E-04 |
| DOM3Z | 0.557 | 0.00016 |
| DONSON | 0.994 | 5.40E-13 |
| DPAGT1 | 0.926 | 0.00094 |
| DPF1 | 0.386 | 6.90E-07 |
| DPP8 | -0.39 | 0.00021 |
| DPYSL3 | 0.345 | 0.00015 |
| DQX1 | 0.248 | 3.20E-06 |
| DRAP1 | 0.691 | 6.70E-06 |
| DRP2 | 0.283 | 4.70E-05 |
| DSCR3 | -1.07 | 1.90E-06 |
| DSG2 | -0.284 | 0.00037 |
| DSN1 | 0.703 | 0.00071 |
| DTX1 | 0.304 | 0.00036 |
| DTX2 | 0.713 | 2.40E-05 |
| DTYMK | 0.615 | 2.40E-05 |
| DULLARD | 1.23 | 5.80E-06 |
| DUS1L | 0.748 | 3.10E-05 |
| DUSP14 | 0.632 | 3.00E-04 |
| DUSP28 | 0.719 | 0.00083 |
| DUSP2 | 0.235 | 0.00059 |
| DUSP3 | -1.01 | 0.00015 |
| DUSP5P | 0.337 | 1.00E-05 |
| DVL3 | 1.27 | 3.30E-09 |
| DYNC1I2 | -1.09 | 1.80E-05 |
| DYNC2H1 | -0.278 | 0.00086 |
| DYNLL2 | -0.803 | 1.40E-08 |
| DYRK4 | 0.906 | 9.70E-07 |
| DZIP3 | -0.456 | 0.00096 |
| E2F3 | 1.19 | 2.10E-07 |
| E2F4 | 1.15 | 6.60E-05 |
| E2F6 | 1.15 | 1.50E-05 |
| E2F7 | 0.46 | 5.00E-07 |
| E4F1 | 0.726 | 8.40E-05 |
| EBI3 | 0.317 | 0.00058 |
| ECE2 | 0.455 | 3.20E-05 |
| ECM1 | 0.341 | 0.00013 |
| EDAR | -0.224 | 7.20E-05 |
| EDA | -0.423 | 1.50E-05 |
| EDEM2 | 0.634 | 0.00041 |
| EDNRB | -0.426 | 1.90E-09 |
| EED | 1.5 | 1.60E-08 |
| EEF1DP3 | -0.437 | 0.00048 |
| EFCAB10 | 0.588 | 1.10E-07 |
| EFCAB6 | -0.323 | 0.00019 |
| EFHD2 | 0.534 | 0.00015 |
| EFNA4 | 0.624 | 0.00026 |
| EFNB2 | -0.36 | 2.00E-04 |
| EGFL6 | 0.168 | 5.70E-06 |
| EGLN1 | -0.667 | 1.80E-05 |
| EHHADH | -0.331 | 2.80E-07 |
| EIF2B1 | 2.11 | 7.70E-08 |
| EIF2B4 | 1.08 | 2.10E-06 |
| EIF2B5 | 1.08 | 0.00079 |
| EIF2S2 | 0.912 | 0.00044 |
| EIF3A | -0.82 | 1.70E-06 |
| EIF3C | -0.181 | 4.00E-04 |
| EIF3L | -0.755 | 0.00054 |
| EIF4B | -0.834 | 4.70E-06 |
| EIF4E3 | -0.6 | 0.00049 |
| EIF4EBP1 | 0.437 | 1.80E-06 |
| EIF4EBP2 | -1.17 | 1.10E-09 |
| EIF5AL1 | 0.879 | 7.20E-06 |
| EIF5A | 0.817 | 1.30E-06 |
| EIF5B | 0.598 | 0.00076 |
| ELK1 | 0.786 | 3.60E-05 |
| ELK3 | -0.298 | 0.00042 |
| ELL3 | 0.702 | 2.70E-07 |
| ELMO2 | 0.916 | 8.10E-05 |
| ELMOD2 | -0.717 | 0.00033 |
| ELMOD3 | 0.845 | 2.90E-06 |
| ELOVL3 | 0.395 | 0.00026 |
| ELOVL7 | -0.275 | 6.80E-06 |
| EMCN | -0.361 | 2.10E-07 |
| EME1 | 0.487 | 5.40E-10 |
| EMP3 | 0.468 | 9.60E-05 |
| EMX2OS | -0.232 | 1.60E-09 |
| EMX2 | -0.233 | 1.00E-08 |
| EN1 | 0.212 | 0.00039 |
| EN2 | 0.248 | 9.00E-07 |
| ENAM | -0.262 | 5.50E-08 |
| ENDOD1 | -0.681 | 4.40E-06 |
| ENGASE | 0.567 | 3.90E-07 |
| ENO3 | 0.305 | 4.70E-05 |
| ENPP4 | -0.528 | 7.10E-09 |
| ENPP5 | -0.546 | 7.40E-11 |
| ENTHD1 | 0.291 | 0.00047 |
| ENTPD5 | -0.358 | 0.00016 |
| ENTPD6 | 0.741 | 2.10E-06 |
| EP400NL | 0.536 | 5.40E-07 |
| EPAS1 | -0.382 | 1.80E-05 |
| EPB41L1 | -0.6 | 6.80E-06 |
| EPB41L4A | -0.5 | 2.20E-05 |
| EPB41L5 | -0.42 | 0.00089 |
| EPC2 | -0.655 | 0.00036 |
| EPHA4 | -0.299 | 0.00031 |
| EPHA8 | 0.324 | 8.50E-05 |
| EPHB2 | 0.303 | 6.50E-05 |
| EPHX2 | -0.502 | 1.60E-07 |
| EPM2A | -0.723 | 0.00041 |
| EPOR | 0.486 | 1.60E-05 |
| EPS8 | -0.421 | 0.00035 |
| EPYC | 0.236 | 9.50E-05 |
| ERBB2 | -0.503 | 0.00012 |
| ERBB3 | -0.31 | 2.50E-07 |
| ERCC4 | -0.585 | 0.00094 |
| ERCC6L | 0.602 | 4.80E-10 |
| EREG | 0.208 | 2.50E-06 |
| ERGIC2 | 0.733 | 0.00089 |
| ERG | -0.319 | 0.00075 |
| ERH | 0.763 | 5.00E-04 |
| ERLIN2 | -0.807 | 1.40E-05 |
| ERMN | 0.339 | 8.30E-06 |
| ERMP1 | -0.568 | 0.00076 |
| ESR2 | 0.322 | 9.50E-06 |
| ESRRG | -0.227 | 2.60E-06 |
| ETFDH | -0.636 | 0.00011 |
| ETV3 | -0.321 | 2.90E-05 |
| ETV6 | 0.966 | 1.50E-07 |
| EVC | -0.44 | 5.70E-05 |
| EVL | 0.643 | 3.40E-06 |
| EWSR1 | 1.16 | 0.00025 |
| EXO1 | 0.378 | 0.00015 |
| EXOC3 | -0.57 | 0.00033 |
| EXOC6B | -0.32 | 1.40E-05 |
| EXOG | 0.787 | 0.00058 |
| EXOSC1 | 0.9 | 4.70E-07 |
| EXOSC8 | 0.788 | 0.00031 |
| EYA1 | 0.173 | 1.70E-05 |
| EYA3 | -0.38 | 4.20E-06 |
| EZH2 | 0.549 | 9.80E-05 |
| EZR | -0.554 | 3.80E-06 |
| F2RL1 | -0.372 | 4.20E-07 |
| F2RL3 | -0.234 | 0.00022 |
| F3 | 0.216 | 9.00E-05 |
| FAAH2 | -0.386 | 8.10E-07 |
| FAAH | -0.325 | 0.00011 |
| FABP3 | -0.269 | 0.00039 |
| FABP5 | 0.293 | 0.00055 |
| FADS1 | 0.493 | 0.001 |
| FADS3 | 0.618 | 1.50E-05 |
| FAHD1 | -0.563 | 0.00034 |
| FAM100B | 0.426 | 0.00016 |
| FAM101A | 0.19 | 6.00E-06 |
| FAM104A | 1.87 | 2.40E-08 |
| FAM107A | -0.349 | 9.60E-06 |
| FAM109A | 0.688 | 2.20E-05 |
| FAM110C | -0.243 | 1.20E-05 |
| FAM111A | 0.587 | 0.00023 |
| FAM113A | 0.607 | 0.00012 |
| FAM113B | 0.355 | 0.00066 |
| FAM114A2 | -0.87 | 0.00035 |
| FAM115A | -0.389 | 3.20E-05 |
| FAM120B | -0.6 | 0.00051 |
| FAM122A | -0.551 | 0.00017 |
| FAM122B | 0.784 | 0.00071 |
| FAM124B | -0.324 | 0.00063 |
| FAM131A | 0.797 | 4.10E-05 |
| FAM134B | -0.27 | 0.00018 |
| FAM136A | 0.97 | 1.80E-05 |
| FAM13AOS | 0.32 | 0.00014 |
| FAM13B | -0.523 | 0.00029 |
| FAM156A | 0.48 | 3.60E-07 |
| FAM158A | 0.438 | 4.70E-05 |
| FAM160A1 | -0.421 | 1.30E-07 |
| FAM160B2 | 0.829 | 1.30E-05 |
| FAM161B | -0.301 | 4.70E-05 |
| FAM168A | -0.438 | 1.40E-05 |
| FAM175A | -0.635 | 3.00E-06 |
| FAM175B | -1.19 | 7.90E-06 |
| FAM178B | 0.321 | 0.00032 |
| FAM179A | 0.357 | 4.40E-06 |
| FAM183B | 0.418 | 2.10E-05 |
| FAM186A | 0.344 | 0.00056 |
| FAM189B | 0.786 | 4.50E-05 |
| FAM18A | 0.4 | 2.70E-05 |
| FAM18B2 | 0.812 | 2.80E-05 |
| FAM190A | -0.335 | 5.70E-06 |
| FAM192A | 1.13 | 0.00036 |
| FAM193B | 0.384 | 7.80E-06 |
| FAM195B | 0.365 | 0.00047 |
| FAM19A3 | 0.339 | 0.00021 |
| FAM20A | 0.41 | 3.70E-08 |
| FAM47E | -0.281 | 0.00026 |
| FAM50A | 0.572 | 0.00021 |
| FAM50B | -0.28 | 9.00E-04 |
| FAM53A | 0.419 | 0.00064 |
| FAM53B | -0.618 | 0.00016 |
| FAM54A | 0.625 | 4.10E-08 |
| FAM57B | 0.276 | 1.20E-06 |
| FAM59A | -0.402 | 7.20E-07 |
| FAM63A | -0.594 | 7.00E-05 |
| FAM63B | -0.461 | 9.50E-06 |
| FAM64A | 0.425 | 1.10E-06 |
| FAM71F2 | 0.253 | 0.001 |
| FAM72A | 0.587 | 7.20E-09 |
| FAM72B | 0.708 | 1.70E-12 |
| FAM72D | 0.616 | 7.60E-11 |
| FAM73A | -0.668 | 9.20E-08 |
| FAM78B | 0.28 | 0.001 |
| FAM7A3 | 0.475 | 1.90E-07 |
| FAM83A | 0.243 | 3.30E-05 |
| FAM83C | 0.507 | 3.20E-05 |
| FAM83D | 0.337 | 0.00048 |
| FAM84A | -0.323 | 0.00094 |
| FAM84B | -0.347 | 5.90E-06 |
| FAM98B | -0.498 | 1.20E-05 |
| FANCA | 0.37 | 6.10E-05 |
| FANCI | 0.74 | 6.10E-06 |
| FANCL | 1.14 | 2.50E-08 |
| FARS2 | -1.22 | 2.40E-07 |
| FASN | 0.493 | 0.00028 |
| FASTKD2 | -1 | 0.00058 |
| FASTK | 0.524 | 0.00027 |
| FAT4 | -0.265 | 0.00029 |
| FBF1 | 0.512 | 6.80E-07 |
| FBN2 | 0.239 | 0.00028 |
| FBRS | 0.933 | 6.00E-05 |
| FBXL16 | -0.165 | 0.00081 |
| FBXL17 | -0.832 | 0.00014 |
| FBXL19 | 0.721 | 2.10E-05 |
| FBXL3 | -0.697 | 2.80E-07 |
| FBXL4 | -0.576 | 0.00095 |
| FBXL5 | -0.803 | 7.00E-09 |
| FBXL6 | 0.609 | 4.80E-07 |
| FBXO18 | 1.92 | 7.90E-05 |
| FBXO21 | -0.441 | 3.70E-05 |
| FBXO22OS | 0.629 | 0.00022 |
| FBXO34 | -0.428 | 0.00076 |
| FBXO38 | -0.601 | 0.00093 |
| FBXO3 | -0.876 | 7.70E-09 |
| FBXO43 | 0.598 | 1.90E-08 |
| FBXO46 | 0.918 | 2.20E-06 |
| FBXO48 | -0.708 | 4.20E-06 |
| FBXO6 | 0.476 | 0.00056 |
| FBXW10 | 0.379 | 0.00043 |
| FBXW11 | -0.871 | 2.40E-05 |
| FBXW7 | 0.908 | 0.00012 |
| FCER1A | -0.336 | 7.30E-09 |
| FCER1G | 0.36 | 0.00056 |
| FCGR1A | 0.313 | 2.00E-04 |
| FCGR1B | 0.394 | 1.00E-05 |
| FCGR1C | 0.404 | 5.10E-06 |
| FCHO1 | 0.237 | 0.00054 |
| FCHO2 | -0.572 | 5.30E-05 |
| FCHSD1 | 0.471 | 0.00012 |
| FDPSL2A | 0.254 | 0.00092 |
| FDPS | -0.952 | 0.00058 |
| FECH | -0.859 | 5.00E-05 |
| FER1L4 | 0.213 | 7.40E-05 |
| FFAR3 | 0.264 | 7.90E-05 |
| FGD1 | 0.378 | 0.00091 |
| FGD5 | -0.355 | 1.90E-05 |
| FGF12 | -0.424 | 3.30E-05 |
| FGF17 | 0.357 | 1.00E-05 |
| FGGY | -0.409 | 0.00092 |
| FHL3 | 0.48 | 6.70E-05 |
| FHL5 | -0.242 | 3.00E-04 |
| FITM2 | -0.761 | 2.70E-08 |
| FKBP10 | 0.443 | 2.40E-06 |
| FKBP11 | 0.557 | 9.40E-09 |
| FKBP3 | 0.938 | 1.10E-05 |
| FKRP | 0.844 | 0.00023 |
| FLAD1 | 0.912 | 1.70E-05 |
| FLJ11235 | -0.536 | 7.50E-07 |
| FLJ14107 | 0.52 | 3.70E-06 |
| FLJ23867 | 0.269 | 0.00072 |
| FLJ35390 | -0.477 | 7.80E-05 |
| FLJ35776 | 0.576 | 0.00013 |
| FLJ36031 | 0.404 | 0.00079 |
| FLJ39653 | 0.363 | 0.00072 |
| FLJ40292 | 0.452 | 2.70E-06 |
| FLJ40330 | 0.229 | 6.20E-06 |
| FLJ45079 | 0.338 | 0.00063 |
| FLJ45340 | 0.355 | 0.001 |
| FLNC | 0.205 | 3.00E-05 |
| FLOT1 | 0.746 | 0.00046 |
| FLRT3 | -0.196 | 2.10E-05 |
| FLT1 | -0.23 | 0.00073 |
| FNBP1L | -0.487 | 1.20E-05 |
| FNBP4 | 0.674 | 6.70E-06 |
| FNDC3A | -0.446 | 0.00043 |
| FNDC4 | 0.204 | 0.00074 |
| FNIP2 | -0.411 | 5.40E-07 |
| FOSL1 | 0.259 | 5.10E-05 |
| FOXA1 | 0.173 | 2.70E-05 |
| FOXA2 | 0.186 | 1.50E-05 |
| FOXE3 | 0.471 | 0.00018 |
| FOXG1 | 0.328 | 3.70E-06 |
| FOXH1 | 0.309 | 9.50E-05 |
| FOXI2 | -0.271 | 6.20E-05 |
| FOXK2 | 1.16 | 1.10E-06 |
| FOXM1 | 0.481 | 2.00E-09 |
| FOXO4 | -0.725 | 0.00084 |
| FOXP3 | 0.208 | 0.00058 |
| FRAS1 | -0.255 | 2.10E-05 |
| FREM1 | -0.175 | 4.70E-05 |
| FREM2 | -0.188 | 7.80E-07 |
| FRMD3 | -0.277 | 2.50E-07 |
| FRMPD2 | -0.337 | 6.10E-06 |
| FRS3 | 0.546 | 0.00046 |
| FRY | -0.409 | 0.00022 |
| FSD1 | 0.317 | 1.30E-06 |
| FSTL4 | -0.249 | 0.00034 |
| FTH1 | 0.649 | 0.00032 |
| FTO | -0.523 | 0.00039 |
| FTSJ1 | 0.622 | 0.00023 |
| FUT10 | -0.68 | 8.50E-05 |
| FUT11 | -0.367 | 0.00015 |
| FUT1 | -0.381 | 0.00075 |
| FUT3 | -0.191 | 2.00E-04 |
| FUT6 | -0.166 | 1.70E-06 |
| FXYD2 | -0.184 | 6.30E-05 |
| FXYD5 | 0.512 | 4.90E-05 |
| FZD2 | 0.333 | 2.20E-05 |
| FZD4 | -0.44 | 7.90E-05 |
| FZD5 | -0.339 | 0.00057 |
| G0S2 | 0.203 | 0.00099 |
| G3BP2 | -0.697 | 0.00049 |
| G6PC | -0.163 | 1.70E-05 |
| G6PD | 0.558 | 0.00017 |
| GAB1 | -0.576 | 0.00014 |
| GAB2 | -0.716 | 7.00E-05 |
| GABBR1 | 0.451 | 2.90E-08 |
| GABRA3 | 0.321 | 0.00042 |
| GABRQ | -0.311 | 4.50E-05 |
| GAL3ST2 | 0.441 | 0.00015 |
| GALK1 | 0.44 | 0.00079 |
| GALK2 | -0.802 | 0.00025 |
| GALNS | 0.538 | 0.00031 |
| GALNT11 | -0.44 | 0.00034 |
| GALNT2 | 0.573 | 5.60E-07 |
| GALNTL2 | -0.293 | 6.80E-06 |
| GALR2 | 0.471 | 0.00036 |
| GARS | 0.635 | 9.80E-05 |
| GATC | -0.297 | 0.00014 |
| GBA3 | -0.15 | 1.70E-06 |
| GBGT1 | 0.581 | 7.40E-05 |
| GBP2 | 0.484 | 8.90E-05 |
| GCC2 | -0.487 | 0.001 |
| GCFC1 | 0.596 | 3.00E-04 |
| GCKR | 0.23 | 1.40E-06 |
| GCLC | -0.587 | 0.00091 |
| GCLM | -0.451 | 0.00082 |
| GCNT2 | -0.433 | 5.60E-05 |
| GCNT4 | -0.317 | 3.30E-06 |
| GCSH | -0.256 | 0.00068 |
| GDA | -0.144 | 0.00037 |
| GDF5 | 0.212 | 0.00057 |
| GDF6 | -0.162 | 0.00059 |
| GDF7 | -0.406 | 2.50E-05 |
| GDPD3 | 0.394 | 2.00E-06 |
| GDPD5 | 0.426 | 0.00018 |
| GEFT | 0.378 | 4.00E-04 |
| GEMIN6 | 0.747 | 8.10E-05 |
| GEMIN7 | 0.766 | 3.80E-06 |
| GEMIN8P4 | 0.51 | 6.90E-05 |
| GEN1 | 0.783 | 2.50E-07 |
| GET4 | 0.677 | 0.00018 |
| GFPT2 | 0.275 | 7.20E-08 |
| GGCT | 0.744 | 0.00075 |
| GGN | 0.333 | 0.00075 |
| GHDC | -0.484 | 0.00083 |
| GHITM | -0.779 | 0.00075 |
| GHRLOS | 0.405 | 1.20E-05 |
| GHR | -0.312 | 5.40E-06 |
| GIGYF2 | -0.925 | 5.70E-05 |
| GIMAP6 | -0.33 | 0.00018 |
| GIMAP8 | -0.31 | 0.00043 |
| GIN1 | -0.918 | 5.20E-05 |
| GINS1 | 0.531 | 8.10E-05 |
| GIPC1 | 0.5 | 0.00087 |
| GIPC2 | -0.236 | 6.50E-06 |
| GIPR | 0.267 | 5.00E-04 |
| GIYD2 | 0.502 | 8.20E-05 |
| GJA5 | -0.313 | 0.00012 |
| GJA9 | 0.623 | 7.20E-05 |
| GJB1 | -0.189 | 3.90E-06 |
| GJB6 | 0.197 | 3.50E-06 |
| GJD4 | 0.743 | 1.40E-06 |
| GK3P | -0.34 | 1.70E-05 |
| GLIPR2 | 0.477 | 0.00041 |
| GLMN | 0.805 | 3.00E-04 |
| GLOD4 | -1.08 | 9.00E-04 |
| GLRX2 | 0.547 | 0.00073 |
| GLT25D1 | 1.11 | 1.10E-09 |
| GLTSCR1 | 0.761 | 1.20E-05 |
| GLUD2 | -0.457 | 2.70E-08 |
| GLYATL1 | -0.133 | 0.00019 |
| GLYAT | -0.109 | 0.00044 |
| GMIP | 0.439 | 0.00071 |
| GNA11 | -0.477 | 5.30E-07 |
| GNA14 | -0.41 | 7.60E-05 |
| GNA15 | 0.441 | 0.00013 |
| GNAS | 0.581 | 1.20E-05 |
| GNB1L | 0.627 | 0.00012 |
| GNB3 | 0.319 | 2.40E-07 |
| GNE | -0.381 | 3.40E-05 |
| GNG12 | -0.743 | 0.00058 |
| GNG3 | 0.483 | 0.00026 |
| GNG5 | 0.96 | 6.10E-05 |
| GNG7 | -0.598 | 1.20E-07 |
| GNL2 | 0.883 | 0.00052 |
| GNL3 | 0.787 | 4.30E-05 |
| GNRH1 | 0.358 | 1.00E-05 |
| GOLGA3 | 0.955 | 7.90E-06 |
| GOLGA6A | 0.506 | 0.00057 |
| GOLGA6B | 0.341 | 0.00026 |
| GOLGA6L10 | 0.376 | 7.80E-05 |
| GOLGA6L5 | 0.296 | 0.00042 |
| GOLGA6L9 | 0.451 | 1.10E-06 |
| GOLGA8A | 0.25 | 6.40E-05 |
| GOLGA8B | 0.303 | 2.40E-06 |
| GOLGA8C | 0.282 | 4.60E-05 |
| GOLIM4 | -0.307 | 7.80E-05 |
| GOLPH3L | -0.84 | 0.00077 |
| GOLT1B | 0.563 | 0.00062 |
| GORAB | 0.725 | 0.00017 |
| GOSR1 | -0.973 | 0.00099 |
| GOT1 | -0.536 | 7.30E-05 |
| GPC1 | 0.442 | 0.00045 |
| GPC2 | 0.48 | 1.80E-07 |
| GPR108 | -0.639 | 0.00021 |
| GPR116 | -0.37 | 1.70E-05 |
| GPR137 | 0.586 | 0.00036 |
| GPR158 | 0.269 | 2.00E-04 |
| GPR160 | -0.437 | 8.90E-05 |
| GPR162 | 0.388 | 1.50E-05 |
| GPR172A | 0.733 | 3.20E-06 |
| GPR173 | 0.327 | 3.00E-06 |
| GPR179 | 0.414 | 0.00046 |
| GPR19 | 0.419 | 0.00021 |
| GPR56 | -0.392 | 0.00012 |
| GPR68 | 0.321 | 0.00055 |
| GPR77 | -0.38 | 1.50E-05 |
| GPR83 | 0.368 | 1.40E-05 |
| GPR84 | 0.38 | 2.80E-06 |
| GPR89B | -0.281 | 0.00036 |
| GPR97 | 0.24 | 0.00026 |
| GPR98 | -0.27 | 0.00061 |
| GPRASP2 | -0.613 | 3.80E-06 |
| GPRC5A | 0.174 | 7.60E-05 |
| GPRIN1 | 0.343 | 0.00099 |
| GPRIN3 | -0.315 | 1.40E-05 |
| GPSM1 | 0.491 | 2.50E-06 |
| GPSM3 | 0.475 | 6.10E-05 |
| GRAMD1A | 0.776 | 1.00E-05 |
| GRAMD1B | 0.283 | 0.00072 |
| GRAMD1C | -0.418 | 5.30E-06 |
| GRB10 | -0.533 | 1.00E-06 |
| GRIN2D | 0.382 | 2.90E-07 |
| GRIPAP1 | 0.839 | 0.00092 |
| GRLF1 | -0.932 | 1.30E-05 |
| GRM2 | 0.313 | 8.00E-04 |
| GRM4 | 0.332 | 2.50E-08 |
| GSDMB | 0.373 | 1.80E-08 |
| GSG2 | 0.436 | 7.40E-05 |
| GSR | -0.562 | 3.40E-05 |
| GTF2A1 | -0.251 | 3.60E-05 |
| GTF2E2 | 1.37 | 2.10E-08 |
| GTF3C4 | -0.511 | 1.40E-05 |
| GTPBP2 | 1.01 | 4.80E-11 |
| GTPBP3 | 0.883 | 8.10E-06 |
| GTSE1 | 0.545 | 4.40E-08 |
| GUCA1B | 0.416 | 3.80E-06 |
| GUSBP3 | 0.452 | 0.00059 |
| GYG2 | 0.166 | 0.00013 |
| GYLTL1B | -0.223 | 5.40E-06 |
| H2AFB1 | 0.471 | 3.30E-05 |
| H2AFX | 0.767 | 1.20E-09 |
| H3F3A | 0.568 | 0.00056 |
| HADHB | -0.788 | 4.00E-04 |
| HADH | -0.992 | 2.90E-05 |
| HAMP | 0.25 | 2.70E-06 |
| HAO2 | -0.142 | 2.00E-05 |
| HAPLN3 | 0.463 | 1.60E-07 |
| HAS1 | 0.196 | 0.00062 |
| HAUS5 | 0.765 | 3.20E-06 |
| HAUS8 | 1.15 | 5.20E-09 |
| HBE1 | 0.235 | 0.00057 |
| HBP1 | -0.792 | 1.10E-06 |
| HCST | 0.349 | 4.80E-05 |
| HDAC1 | 1.18 | 0.00014 |
| HDAC8 | 1 | 0.00024 |
| HDHD2 | -0.886 | 0.00091 |
| HEBP2 | 0.504 | 0.00033 |
| HECW2 | -0.232 | 0.00059 |
| HELQ | -0.822 | 0.00033 |
| HEPHL1 | 0.336 | 6.00E-05 |
| HERC1 | -0.591 | 0.00073 |
| HERC2P2 | 0.304 | 2.80E-05 |
| HERC3 | -0.312 | 0.00027 |
| HERC4 | 0.942 | 0.00068 |
| HERPUD1 | -0.855 | 4.60E-05 |
| HES7 | 0.587 | 3.10E-09 |
| HESX1 | 0.538 | 9.10E-06 |
| HFE | -0.416 | 0.00023 |
| HGS | 1.09 | 1.00E-06 |
| HHAT | -0.487 | 0.00042 |
| HHIPL2 | 0.435 | 5.50E-12 |
| HHLA2 | -0.136 | 9.90E-06 |
| HHLA3 | 0.528 | 0.00042 |
| HIATL2 | 0.458 | 0.001 |
| HIBCH | -0.762 | 8.90E-08 |
| HIPK2 | -0.541 | 2.50E-06 |
| HIPK3 | -0.322 | 1.30E-05 |
| HIRA | -0.588 | 0.00094 |
| HIST1H1D | 0.335 | 2.30E-05 |
| HIST1H1E | 0.512 | 1.30E-09 |
| HIST1H2AD | 0.326 | 2.00E-04 |
| HIST1H2AM | 0.342 | 7.80E-05 |
| HIST1H2BF | 0.337 | 1.10E-05 |
| HIST1H2BG | 0.235 | 0.00035 |
| HIST1H2BH | 0.359 | 0.00011 |
| HIST1H3D | 0.264 | 0.00029 |
| HIST1H4E | 0.313 | 0.00014 |
| HIST2H2AA3 | 0.294 | 0.00037 |
| HIST2H2AC | 0.494 | 1.20E-06 |
| HIST2H2BF | 0.292 | 0.00042 |
| HIST2H3C | 0.365 | 0.00074 |
| HIST2H3D | 0.468 | 0.00099 |
| HJURP | 0.524 | 3.90E-10 |
| HK3 | 0.396 | 6.80E-06 |
| HLA-E | -0.53 | 0.001 |
| HLF | -0.438 | 3.70E-10 |
| HM13 | 0.551 | 0.00084 |
| HMBS | 0.469 | 6.20E-05 |
| HMGA1 | 0.319 | 0.00031 |
| HMGA2 | 0.195 | 1.20E-06 |
| HMGB2 | 0.985 | 6.50E-07 |
| HMGB3 | 0.341 | 0.00037 |
| HMGCLL1 | -0.246 | 9.20E-05 |
| HMGCR | -0.752 | 0.00019 |
| HMGCS1 | -0.86 | 2.70E-05 |
| HMGCS2 | -0.131 | 4.70E-05 |
| HMGN1 | 1.01 | 5.60E-06 |
| HMGN3 | 0.474 | 0.00037 |
| HMMR | 0.347 | 0.00014 |
| HMP19 | 0.34 | 9.10E-05 |
| HN1 | 0.615 | 4.60E-08 |
| HNMT | -0.506 | 0.00021 |
| HNRNPA2B1 | 1.32 | 4.40E-06 |
| HNRNPAB | 0.64 | 0.00089 |
| HNRNPL | 1.84 | 1.50E-06 |
| HNRNPUL2 | -0.926 | 8.40E-05 |
| HOOK1 | -0.332 | 6.80E-08 |
| HOXA11AS | 0.254 | 0.00072 |
| HOXA1 | 0.438 | 3.20E-05 |
| HOXA2 | 0.365 | 1.40E-06 |
| HOXA3 | 0.394 | 0.00056 |
| HOXB13 | 0.18 | 2.30E-06 |
| HOXC13 | 0.263 | 0.00055 |
| HOXC5 | 0.445 | 7.00E-04 |
| HPX | 0.196 | 6.50E-07 |
| HP | 0.0867 | 0.00052 |
| HRH2 | -0.188 | 5.30E-05 |
| HRNBP3 | 0.462 | 1.20E-05 |
| HSD17B3 | 0.222 | 6.70E-05 |
| HSD17B7P2 | 0.349 | 0.00072 |
| HSD17B8 | -0.534 | 2.00E-06 |
| HSF2BP | 0.341 | 8.00E-05 |
| HSH2D | 0.302 | 3.20E-05 |
| HSN2 | 0.352 | 2.90E-05 |
| HSP90AB2P | -0.292 | 0.00021 |
| HSP90AB4P | 0.405 | 3.00E-08 |
| HSPA1L | -0.733 | 0.00048 |
| HSPA4L | -0.399 | 3.70E-07 |
| HSPA6 | 0.421 | 3.00E-05 |
| HSPA7 | 0.292 | 1.70E-05 |
| HSPA8 | -0.537 | 0.00011 |
| HSPB11 | 1.01 | 2.50E-06 |
| HSPBAP1 | 0.707 | 6.50E-06 |
| HSPC159 | -0.548 | 0.00083 |
| HTATIP2 | 0.668 | 3.70E-05 |
| HTR1D | 0.27 | 3.40E-05 |
| HTR3A | 0.203 | 0.00058 |
| HUNK | -0.191 | 0.00048 |
| HUS1B | 0.421 | 2.90E-05 |
| HYAL1 | -0.404 | 2.70E-06 |
| HYMAI | 0.338 | 0.00038 |
| IARS2 | -0.691 | 0.00043 |
| IBSP | 0.158 | 0.00018 |
| ICAM4 | 0.334 | 8.00E-04 |
| ICAM5 | 0.222 | 0.00047 |
| IDUA | 0.582 | 5.00E-07 |
| IFFO1 | 0.637 | 1.40E-06 |
| IFI16 | 0.574 | 0.00042 |
| IFI44 | 0.451 | 0.00018 |
| IFIH1 | -0.425 | 0.00053 |
| IFITM1 | 0.521 | 4.90E-06 |
| IFITM2 | 0.486 | 0.00018 |
| IFITM3 | 0.63 | 5.50E-05 |
| IFNAR1 | -0.796 | 0.00049 |
| IFNE | 0.354 | 4.70E-08 |
| IFNGR2 | 0.759 | 0.00044 |
| IFNG | 0.194 | 0.00026 |
| IFRD1 | 0.782 | 1.80E-07 |
| IFT20 | 0.89 | 5.50E-06 |
| IFT27 | 0.59 | 0.00053 |
| IGDCC4 | 0.189 | 9.40E-06 |
| IGF2BP2 | 0.182 | 0.00012 |
| IGF2BP3 | 0.235 | 3.60E-07 |
| IGFL2 | 0.319 | 1.30E-06 |
| IGFN1 | 0.15 | 3.50E-06 |
| IGHMBP2 | 0.799 | 1.20E-05 |
| IKBKE | 0.432 | 1.60E-05 |
| IKBKG | 0.878 | 0.00077 |
| IL11 | 0.235 | 0.00015 |
| IL12A | 0.362 | 0.001 |
| IL15RA | 0.582 | 5.40E-06 |
| IL17RD | -0.33 | 3.70E-05 |
| IL1RN | 0.232 | 0.00062 |
| IL20RB | 0.147 | 1.90E-05 |
| IL23A | 0.375 | 0.00044 |
| IL27 | 0.374 | 1.30E-05 |
| IL31RA | 0.262 | 2.50E-05 |
| IL4I1 | 0.43 | 4.30E-05 |
| IL6R | -0.344 | 1.60E-05 |
| IL6ST | -0.242 | 1.10E-05 |
| IL6 | 0.19 | 4.50E-07 |
| ILDR2 | -0.198 | 0.00045 |
| IMPA2 | -0.555 | 4.30E-09 |
| IMPDH1 | 0.855 | 2.80E-09 |
| INADL | -0.365 | 2.10E-07 |
| INCA1 | 0.66 | 5.40E-05 |
| INE1 | 0.4 | 0.00012 |
| INGX | 0.555 | 7.60E-05 |
| INHBE | 0.187 | 0.00022 |
| INPP5E | 0.666 | 1.80E-05 |
| INSL3 | 0.309 | 0.00014 |
| INSR | -0.361 | 0.00057 |
| INTS3 | 0.843 | 2.00E-06 |
| INTS8 | 1.04 | 2.70E-05 |
| IP6K2 | 0.872 | 7.30E-06 |
| IPMK | -0.27 | 0.00047 |
| IPO11 | -0.726 | 2.20E-05 |
| IPP | -0.697 | 4.70E-07 |
| IQGAP2 | -0.531 | 2.40E-10 |
| IQGAP3 | 0.449 | 8.70E-07 |
| IQSEC2 | 0.708 | 0.00068 |
| IQUB | -0.253 | 5.10E-05 |
| IREB2 | -0.563 | 0.00044 |
| IRF3 | 0.79 | 5.80E-07 |
| IRF6 | -0.255 | 2.30E-07 |
| IRF7 | 0.581 | 3.70E-08 |
| ISG15 | 0.36 | 5.00E-06 |
| ISG20 | 0.484 | 1.10E-05 |
| ISL2 | 0.427 | 3.70E-09 |
| ISPD | -0.596 | 1.70E-05 |
| ITFG1 | -0.797 | 6.90E-07 |
| ITGA2B | 0.266 | 6.80E-05 |
| ITGA2 | -0.46 | 2.50E-06 |
| ITGA6 | -0.659 | 7.00E-10 |
| ITGA8 | -0.261 | 9.10E-10 |
| ITGA9 | -0.307 | 5.40E-06 |
| ITGAE | 0.754 | 2.90E-07 |
| ITGB1BP1 | 0.719 | 5.00E-05 |
| ITGB1BP2 | 0.331 | 0.00015 |
| ITIH4 | 0.206 | 2.10E-07 |
| ITM2A | -0.308 | 9.00E-04 |
| ITM2B | -0.689 | 7.70E-06 |
| ITPA | 0.656 | 0.00031 |
| ITPKA | 0.3 | 5.90E-10 |
| IVNS1ABP | -0.484 | 0.00016 |
| IYD | -0.191 | 6.40E-05 |
| JAK1 | -0.721 | 2.50E-06 |
| JAK3 | 0.41 | 4.00E-06 |
| JMJD6 | 0.773 | 3.80E-05 |
| JMJD7-PLA2G4B | 0.533 | 1.30E-05 |
| JPH3 | 0.24 | 0.00025 |
| JSRP1 | 0.207 | 1.10E-05 |
| JUP | -0.442 | 3.80E-05 |
| KAT2A | 0.609 | 4.80E-08 |
| KAT5 | -1.09 | 3.30E-06 |
| KBTBD3 | -0.924 | 1.50E-07 |
| KBTBD4 | -0.224 | 6.00E-04 |
| KCNAB1 | -0.304 | 0.00044 |
| KCNAB3 | 0.322 | 1.00E-05 |
| KCNB1 | -0.24 | 0.00065 |
| KCND1 | 0.505 | 6.40E-09 |
| KCNE1L | 0.29 | 2.00E-07 |
| KCNG1 | 0.207 | 5.50E-05 |
| KCNH1 | 0.363 | 7.20E-06 |
| KCNH3 | 0.211 | 0.00025 |
| KCNIP2 | 0.337 | 3.20E-05 |
| KCNJ14 | 0.522 | 2.50E-05 |
| KCNJ15 | -0.252 | 3.50E-07 |
| KCNJ3 | -0.196 | 7.10E-05 |
| KCNK12 | 0.485 | 1.30E-05 |
| KCNK17 | 0.189 | 6.50E-05 |
| KCNK5 | -0.253 | 0.00056 |
| KCNMB3 | 0.451 | 7.60E-07 |
| KCNN3 | -0.36 | 8.50E-07 |
| KCNN4 | 0.351 | 1.40E-06 |
| KCTD13 | 0.567 | 0.00077 |
| KCTD16 | -0.251 | 1.10E-05 |
| KCTD17 | 0.587 | 2.90E-07 |
| KCTD18 | -0.714 | 0.00049 |
| KCTD5 | 1.41 | 4.20E-06 |
| KDELR3 | 0.36 | 1.90E-05 |
| KDM2B | 0.912 | 0.00094 |
| KDM3B | -0.604 | 3.90E-05 |
| KDR | -0.302 | 1.80E-06 |
| KDSR | -0.942 | 4.80E-05 |
| KHDRBS3 | -0.459 | 0.00011 |
| KIAA0101 | 0.46 | 9.80E-06 |
| KIAA0196 | -0.596 | 0.00029 |
| KIAA0232 | -0.656 | 0.00066 |
| KIAA0355 | -0.596 | 0.00076 |
| KIAA0415 | 0.692 | 0.00012 |
| KIAA0430 | -0.745 | 0.00014 |
| KIAA0494 | -1.06 | 1.20E-06 |
| KIAA0562 | -0.753 | 0.00029 |
| KIAA0564 | -0.487 | 3.30E-05 |
| KIAA0652 | 1.17 | 0.00018 |
| KIAA0754 | -0.273 | 1.70E-05 |
| KIAA0907 | 0.503 | 2.30E-05 |
| KIAA1024 | 0.494 | 2.50E-07 |
| KIAA1109 | -0.417 | 0.00018 |
| KIAA1143 | -0.757 | 0.00019 |
| KIAA1147 | -0.574 | 2.20E-06 |
| KIAA1161 | -0.625 | 6.40E-08 |
| KIAA1191 | -0.685 | 2.30E-06 |
| KIAA1310 | 1.44 | 3.00E-06 |
| KIAA1324 | 0.329 | 1.40E-06 |
| KIAA1328 | -0.577 | 0.00092 |
| KIAA1539 | 0.687 | 0.00096 |
| KIAA1549 | -0.51 | 1.50E-07 |
| KIAA1614 | -0.404 | 9.90E-06 |
| KIAA1644 | 0.199 | 6.00E-05 |
| KIAA1671 | -0.602 | 6.90E-08 |
| KIAA1704 | -0.709 | 2.30E-05 |
| KIAA1841 | 0.749 | 2.40E-05 |
| KIAA1875 | 0.245 | 8.90E-05 |
| KIAA1949 | 0.737 | 8.10E-06 |
| KIAA1958 | -0.426 | 8.80E-05 |
| KIF11 | 0.501 | 4.30E-05 |
| KIF13A | -0.486 | 7.80E-05 |
| KIF13B | -0.599 | 0.00013 |
| KIF14 | 0.45 | 7.20E-07 |
| KIF15 | 0.378 | 2.70E-05 |
| KIF18A | 0.559 | 8.30E-08 |
| KIF18B | 0.519 | 2.70E-11 |
| KIF19 | 0.331 | 0.00013 |
| KIF1B | -0.609 | 0.00059 |
| KIF20A | 0.41 | 1.70E-07 |
| KIF22 | 0.668 | 8.00E-06 |
| KIF23 | 0.547 | 6.40E-09 |
| KIF2C | 0.529 | 1.30E-08 |
| KIF3B | -0.999 | 1.60E-05 |
| KIF4A | 0.511 | 5.90E-08 |
| KIF4B | 0.541 | 1.40E-05 |
| KIF7 | 0.39 | 0.00017 |
| KIFC1 | 0.507 | 3.60E-06 |
| KIFC2 | 0.368 | 9.10E-05 |
| KILLIN | -0.579 | 0.00015 |
| KISS1 | 0.243 | 8.50E-05 |
| KITLG | -0.347 | 0.00074 |
| KIT | -0.259 | 0.00082 |
| KLC4 | -0.628 | 0.00011 |
| KLF11 | -0.723 | 0.00027 |
| KLF12 | -0.567 | 1.00E-04 |
| KLF13 | -0.485 | 0.00058 |
| KLF15 | -0.285 | 2.40E-05 |
| KLF6 | -0.377 | 0.00051 |
| KLF9 | -0.479 | 2.40E-05 |
| KLHDC10 | -0.417 | 5.30E-05 |
| KLHDC2 | -0.698 | 0.00092 |
| KLHDC7A | -0.265 | 1.30E-07 |
| KLHDC7B | 0.246 | 4.10E-05 |
| KLHL11 | -0.367 | 3.70E-05 |
| KLHL13 | -0.375 | 0.00086 |
| KLHL17 | 0.393 | 0.00019 |
| KLHL32 | -0.251 | 0.00012 |
| KLHL8 | -0.657 | 2.50E-05 |
| KLHL9 | -0.908 | 9.00E-07 |
| KLK14 | 0.503 | 1.20E-07 |
| KLRA1 | 0.28 | 0.00055 |
| KLRC2 | 0.309 | 7.40E-05 |
| KL | -0.304 | 7.00E-09 |
| KNTC1 | 0.536 | 5.20E-05 |
| KPNA2 | 0.628 | 1.30E-06 |
| KPNA5 | -0.372 | 0.00065 |
| KPTN | 0.628 | 3.70E-05 |
| KRBA2 | -0.406 | 0.00022 |
| KREMEN2 | 0.386 | 2.40E-08 |
| KRI1 | 1.14 | 5.70E-07 |
| KRT10 | 0.534 | 0.00016 |
| KRT13 | 0.144 | 0.00065 |
| KRT15 | 0.219 | 1.10E-06 |
| KRT16 | 0.197 | 8.00E-04 |
| KRT19 | 0.128 | 0.00057 |
| KRT32 | 0.306 | 0.00039 |
| KRTAP5-10 | 0.314 | 0.00099 |
| KRTAP5-1 | 0.226 | 0.001 |
| KRTCAP2 | 0.596 | 6.60E-06 |
| L2HGDH | -0.43 | 0.00017 |
| LAG3 | 0.208 | 5.00E-05 |
| LAGE3 | 0.611 | 4.70E-07 |
| LAMB2 | -0.466 | 0.00013 |
| LANCL1 | -0.701 | 4.20E-05 |
| LASS5 | 1.61 | 3.80E-07 |
| LAT | 0.354 | 6.80E-06 |
| LBP | 0.0801 | 0.00082 |
| LBX2 | 0.643 | 1.00E-09 |
| LCAT | 0.338 | 0.00024 |
| LCMT2 | -1.06 | 4.60E-07 |
| LCNL1 | 0.423 | 1.70E-05 |
| LCTL | 0.426 | 3.00E-11 |
| LDB2 | -0.3 | 0.00037 |
| LDHAL6B | 0.428 | 0.00041 |
| LDHD | -0.285 | 0.00012 |
| LDOC1L | -0.715 | 2.40E-06 |
| LEMD2 | 1.07 | 3.60E-05 |
| LENG8 | 0.333 | 0.00019 |
| LENG9 | 0.366 | 2.00E-05 |
| LEPRE1 | 0.707 | 2.40E-10 |
| LEPROT | -0.386 | 1.10E-07 |
| LGALS1 | 0.463 | 2.40E-05 |
| LGALS3BP | 0.468 | 4.50E-05 |
| LGR4 | -0.379 | 0.00011 |
| LHB | 0.617 | 2.20E-06 |
| LHX2 | 0.173 | 0.00022 |
| LIFR | -0.375 | 8.40E-05 |
| LIF | 0.284 | 7.10E-06 |
| LILRB3 | 0.334 | 0.00026 |
| LILRP2 | 0.267 | 0.00074 |
| LIMCH1 | -0.697 | 2.40E-09 |
| LIMD2 | 0.416 | 8.00E-06 |
| LIMK1 | 0.604 | 1.20E-07 |
| LIN54 | -0.751 | 7.00E-04 |
| LIN7A | -0.252 | 5.60E-06 |
| LMBR1L | 0.726 | 2.10E-06 |
| LMBRD1 | -0.484 | 1.00E-05 |
| LMBRD2 | -0.345 | 2.80E-05 |
| LMNB1 | 0.531 | 0.00012 |
| LMNB2 | 0.859 | 2.70E-05 |
| LMO1 | 0.176 | 0.00035 |
| LMO7 | -0.543 | 8.00E-07 |
| LMOD3 | 0.377 | 0.001 |
| LMTK2 | -0.345 | 3.40E-06 |
| LNPEP | -0.312 | 0.00065 |
| LNX2 | -0.797 | 1.40E-07 |
| LOC100128076 | 0.385 | 4.10E-07 |
| LOC100128191 | 0.746 | 2.10E-05 |
| LOC100128288 | 0.416 | 6.30E-05 |
| LOC100128292 | 0.478 | 0.00016 |
| LOC100128788 | 0.473 | 0.00038 |
| LOC100129055 | 0.566 | 7.50E-06 |
| LOC100129637 | 0.548 | 1.20E-05 |
| LOC100130776 | 0.428 | 1.60E-05 |
| LOC100130872 | 0.251 | 0.00064 |
| LOC100130932 | 0.721 | 1.70E-06 |
| LOC100131434 | 0.244 | 0.00054 |
| LOC100132247 | 0.296 | 0.00024 |
| LOC100132287 | 0.436 | 1.40E-05 |
| LOC100133161 | 0.357 | 2.30E-05 |
| LOC100133545 | 0.24 | 0.00016 |
| LOC100134259 | 0.498 | 0.00013 |
| LOC100144603 | 0.337 | 0.00026 |
| LOC100144604 | 0.388 | 3.70E-07 |
| LOC100190939 | 0.74 | 4.30E-06 |
| LOC100270710 | 0.402 | 5.50E-05 |
| LOC100270804 | 0.381 | 3.10E-05 |
| LOC100272146 | 0.636 | 8.10E-10 |
| LOC100272228 | 0.273 | 0.00013 |
| LOC100286793 | 0.76 | 2.30E-06 |
| LOC121952 | 0.354 | 0.00097 |
| LOC145474 | 0.588 | 8.50E-09 |
| LOC146880 | 0.376 | 4.70E-05 |
| LOC148413 | 0.791 | 0.00036 |
| LOC149134 | 0.358 | 6.30E-06 |
| LOC150197 | 0.359 | 1.30E-06 |
| LOC150776 | 0.561 | 9.90E-08 |
| LOC153328 | -0.206 | 8.30E-05 |
| LOC221442 | 0.235 | 8.00E-04 |
| LOC26102 | 0.368 | 0.00013 |
| LOC283392 | -0.166 | 4.00E-05 |
| LOC283663 | 0.321 | 8.20E-06 |
| LOC283761 | 0.306 | 6.00E-04 |
| LOC284233 | 0.249 | 2.00E-04 |
| LOC284441 | -0.275 | 0.00067 |
| LOC284837 | 0.37 | 0.00014 |
| LOC285074 | 0.718 | 0.00011 |
| LOC285359 | 0.667 | 1.20E-06 |
| LOC285456 | -0.529 | 0.00067 |
| LOC285768 | -0.139 | 8.00E-04 |
| LOC286467 | 0.385 | 1.20E-10 |
| LOC338799 | 0.343 | 2.80E-05 |
| LOC339047 | 0.41 | 2.00E-04 |
| LOC339240 | 0.826 | 6.80E-08 |
| LOC340508 | 0.365 | 1.80E-05 |
| LOC349114 | 0.266 | 0.00058 |
| LOC374443 | 0.603 | 0.00031 |
| LOC375190 | 0.589 | 0.00046 |
| LOC388692 | 0.518 | 2.50E-06 |
| LOC388796 | 0.674 | 2.00E-08 |
| LOC389634 | 0.354 | 1.50E-05 |
| LOC399744 | 0.56 | 3.00E-04 |
| LOC400696 | 0.181 | 0.00024 |
| LOC400931 | 0.319 | 7.90E-05 |
| LOC402377 | -0.39 | 8.30E-05 |
| LOC440896 | 0.454 | 3.80E-06 |
| LOC440944 | 0.476 | 2.40E-05 |
| LOC55908 | 0.123 | 0.00031 |
| LOC642826 | 0.354 | 0.00043 |
| LOC642846 | 0.31 | 8.70E-05 |
| LOC643719 | 0.348 | 0.00019 |
| LOC644936 | 0.484 | 0.00059 |
| LOC645431 | 0.357 | 2.60E-05 |
| LOC646214 | 0.578 | 0.00027 |
| LOC646851 | 0.331 | 0.00037 |
| LOC653501 | -0.554 | 3.40E-05 |
| LOC653653 | -0.367 | 3.00E-05 |
| LOC678655 | 0.915 | 1.60E-08 |
| LOC727896 | 0.502 | 8.00E-07 |
| LOC728024 | -0.492 | 7.10E-06 |
| LOC728392 | 0.339 | 3.00E-04 |
| LOC728743 | 0.467 | 4.00E-07 |
| LOC728855 | 0.479 | 0.00044 |
| LOC729020 | -0.494 | 6.20E-05 |
| LOC729234 | 0.412 | 1.70E-05 |
| LOC730668 | 0.441 | 1.30E-05 |
| LOC90110 | 0.844 | 0.00013 |
| LOC90784 | 0.733 | 0.00014 |
| LOC91450 | 0.461 | 1.20E-06 |
| LOC92659 | 0.304 | 0.00085 |
| LONP2 | -0.45 | 0.00073 |
| LOXL1 | 0.247 | 0.00027 |
| LOXL3 | 0.478 | 2.70E-05 |
| LPAR2 | 0.502 | 8.60E-06 |
| LPCAT4 | 0.846 | 1.50E-05 |
| LPO | 0.479 | 8.60E-08 |
| LPPR2 | 0.743 | 3.10E-05 |
| LPPR3 | 0.271 | 5.90E-05 |
| LRAT | 0.225 | 0.00057 |
| LRBA | -0.937 | 1.60E-09 |
| LRCH1 | -0.505 | 4.00E-04 |
| LRCH4 | 0.583 | 0.00026 |
| LRDD | 0.494 | 2.30E-06 |
| LRFN1 | 0.491 | 6.20E-06 |
| LRFN4 | 0.331 | 3.00E-04 |
| LRP2 | -0.104 | 0.00011 |
| LRP5 | -0.42 | 7.50E-07 |
| LRP8 | 0.622 | 1.80E-10 |
| LRPPRC | -0.934 | 1.30E-06 |
| LRRC15 | 0.142 | 0.00075 |
| LRRC19 | -0.233 | 1.20E-07 |
| LRRC37A2 | 0.422 | 1.60E-05 |
| LRRC37A3 | 0.437 | 0.00021 |
| LRRC37A | 0.527 | 6.20E-06 |
| LRRC39 | 0.307 | 2.80E-05 |
| LRRC4C | 0.261 | 1.50E-06 |
| LRRC55 | -0.348 | 2.80E-05 |
| LRRC61 | 0.456 | 0.00019 |
| LSM10 | 0.788 | 3.90E-06 |
| LSM12 | 1.33 | 4.60E-10 |
| LSM1 | 0.87 | 6.60E-05 |
| LSM7 | 0.558 | 2.60E-05 |
| LTB4R2 | 0.37 | 0.00064 |
| LTB4R | 0.523 | 1.30E-07 |
| LTV1 | 1.01 | 0.00017 |
| LUC7L3 | 0.68 | 6.50E-07 |
| LUC7L | 0.521 | 1.90E-06 |
| LUZP1 | -0.689 | 0.00015 |
| LY6G5B | 0.262 | 0.00076 |
| LY96 | 0.334 | 0.00034 |
| LYG2 | 0.378 | 0.00063 |
| LYPD1 | 0.213 | 0.00042 |
| LYPD3 | 0.294 | 0.00072 |
| LYRM7 | -0.823 | 1.90E-05 |
| LYSMD3 | -0.544 | 1.00E-04 |
| LZIC | -0.962 | 8.10E-06 |
| LZTFL1 | -0.805 | 0.00017 |
| LZTS2 | 0.878 | 1.20E-06 |
| MACC1 | -0.275 | 5.50E-06 |
| MAGED4B | 0.169 | 6.00E-05 |
| MAGED4 | 0.193 | 6.80E-06 |
| MAGEE1 | -0.644 | 0.00042 |
| MAGI1 | -0.476 | 6.10E-05 |
| MAGOHB | 0.713 | 6.60E-05 |
| MAML2 | -0.269 | 2.00E-04 |
| MAML3 | -0.562 | 3.10E-05 |
| MAN1A2 | -0.405 | 0.00071 |
| MAN2A1 | -0.324 | 7.00E-05 |
| MAN2A2 | 0.804 | 0.00085 |
| MANSC1 | -0.552 | 1.50E-05 |
| MAP2K5 | -0.89 | 0.00047 |
| MAP3K12 | 0.594 | 4.20E-06 |
| MAP3K8 | 0.536 | 1.90E-06 |
| MAP6D1 | 0.599 | 3.50E-11 |
| MAP7D1 | 0.846 | 5.30E-05 |
| MAP7 | -0.433 | 6.70E-10 |
| MAPK10 | -0.435 | 0.00083 |
| MAPK1 | -0.554 | 0.00013 |
| MAPK8IP1 | -0.352 | 0.00037 |
| MAPK8IP3 | 0.367 | 4.30E-05 |
| MAPK8 | -0.27 | 3.00E-05 |
| MAPT | -0.213 | 6.40E-05 |
| 6-Mar | -0.503 | 0.00087 |
| 8-Mar | -0.382 | 4.20E-06 |
| MARS | 1.03 | 4.00E-08 |
| MASP1 | -0.152 | 0.001 |
| MAST1 | 0.243 | 0.00052 |
| MAST2 | 0.891 | 1.00E-06 |
| MAST4 | -0.367 | 1.20E-06 |
| MAT1A | 0.159 | 3.80E-06 |
| MAT2B | -0.543 | 6.20E-05 |
| MATN1 | 0.557 | 5.20E-07 |
| MATR3 | -1.01 | 7.00E-05 |
| MAZ | 1.09 | 3.30E-06 |
| MBD6 | 0.829 | 0.00018 |
| MBLAC2 | -0.669 | 1.90E-05 |
| MBNL3 | -0.209 | 0.001 |
| MBOAT7 | 0.792 | 9.30E-08 |
| MBTPS1 | -0.912 | 0.00065 |
| MC1R | 0.589 | 5.70E-10 |
| MCF2L2 | 0.426 | 1.10E-05 |
| MCFD2 | -0.585 | 0.001 |
| MCM10 | 0.384 | 0.00043 |
| MCTS1 | 0.909 | 2.50E-06 |
| MDGA1 | 0.372 | 0.00036 |
| MDK | 0.317 | 4.80E-07 |
| MDM4 | 0.435 | 0.00097 |
| MEA1 | 0.789 | 4.60E-05 |
| MECP2 | -0.875 | 6.00E-04 |
| MED15 | 0.828 | 0.00019 |
| MED26 | 1.12 | 1.90E-05 |
| MED8 | 1.32 | 1.40E-05 |
| MEF2A | -0.567 | 0.00042 |
| MEF2B | 0.385 | 8.10E-06 |
| MEG3 | 0.194 | 0.00036 |
| MEGF9 | -0.494 | 1.30E-05 |
| MEI1 | 0.265 | 0.00035 |
| MEIS3 | 0.367 | 1.30E-05 |
| MELK | 0.489 | 1.70E-06 |
| MEMO1 | 0.877 | 2.00E-07 |
| MERTK | -0.453 | 7.40E-05 |
| METTL14 | -0.608 | 5.00E-07 |
| METTL1 | 0.552 | 0.00052 |
| METTL6 | 1.16 | 1.00E-06 |
| METTL7A | -0.621 | 6.40E-09 |
| METTL9 | -0.741 | 7.40E-05 |
| MFAP1 | -1.33 | 5.40E-07 |
| MFAP3L | -0.345 | 4.90E-05 |
| MFHAS1 | -0.313 | 6.50E-05 |
| MFI2 | 0.27 | 1.30E-08 |
| MFSD10 | 0.604 | 0.00023 |
| MFSD2A | 0.216 | 1.80E-05 |
| MFSD2B | 0.298 | 0.00042 |
| MFSD4 | -0.532 | 2.40E-07 |
| MFSD5 | 0.679 | 0.00013 |
| MFSD6 | -0.572 | 3.00E-04 |
| MFSD9 | -0.556 | 0.00038 |
| MGAM | -0.217 | 1.50E-08 |
| MGAT5 | -0.236 | 1.10E-05 |
| MGC2889 | 0.322 | 0.00061 |
| MGC29506 | 0.144 | 0.00078 |
| MGC72080 | 0.488 | 0.00019 |
| MIA2 | -0.268 | 2.60E-09 |
| MIAT | 0.281 | 3.10E-07 |
| MIA | 0.225 | 2.00E-05 |
| MICAL1 | 0.661 | 3.70E-07 |
| MICALL2 | 0.617 | 1.50E-07 |
| MIER2 | 0.742 | 0.00017 |
| MIIP | 0.532 | 2.40E-07 |
| MINA | 0.411 | 0.00074 |
| MIPEP | -0.95 | 3.20E-07 |
| MIR155HG | 0.282 | 1.70E-05 |
| MITD1 | 1.21 | 9.50E-07 |
| MKI67IP | 0.83 | 0.00023 |
| MKI67 | 0.371 | 0.00028 |
| MKL1 | 0.764 | 0.00015 |
| MKL2 | -0.61 | 0.00088 |
| MKNK1 | 1.01 | 6.80E-05 |
| MKS1 | 0.802 | 0.00095 |
| MLLT11 | 0.382 | 3.10E-07 |
| MLNR | -0.416 | 0.001 |
| MLYCD | -0.543 | 6.80E-05 |
| MMAA | -0.887 | 4.90E-06 |
| MMGT1 | -0.701 | 1.10E-06 |
| MMP12 | 0.139 | 0.00024 |
| MMP14 | 0.464 | 0.00019 |
| MMP17 | 0.271 | 2.30E-07 |
| MMP19 | 0.537 | 3.90E-07 |
| MMP3 | 0.222 | 2.00E-05 |
| MMP9 | 0.177 | 1.00E-04 |
| MMRN2 | -0.371 | 2.10E-05 |
| MND1 | 0.345 | 0.00045 |
| MOAP1 | -0.979 | 1.20E-06 |
| MOBKL2B | -0.408 | 1.10E-08 |
| MOCOS | 0.232 | 4.00E-06 |
| MOGS | 1.43 | 1.50E-07 |
| MOSC2 | -0.604 | 2.10E-06 |
| MOSPD2 | -0.566 | 1.40E-05 |
| MOV10 | 1.2 | 2.10E-08 |
| MPG | 0.487 | 5.00E-04 |
| MPHOSPH10 | 1.08 | 0.00011 |
| MPHOSPH8 | -0.596 | 0.00017 |
| MPL | -0.41 | 0.00081 |
| MPP2 | 0.207 | 0.00057 |
| MPP3 | 0.402 | 2.00E-05 |
| MPP4 | 0.717 | 7.40E-11 |
| MPP5 | -0.875 | 2.70E-07 |
| MPP7 | -0.423 | 0.00012 |
| MPPED2 | -0.366 | 2.30E-05 |
| MPV17L | -0.19 | 0.00029 |
| MPZL2 | -0.441 | 7.00E-06 |
| MPZL3 | -0.382 | 7.60E-06 |
| MRC2 | 0.363 | 4.50E-05 |
| MRPL14 | 0.618 | 9.40E-05 |
| MRPL17 | 0.815 | 1.00E-06 |
| MRPL18 | 0.759 | 7.10E-05 |
| MRPL40 | 0.547 | 0.00047 |
| MRPL41 | 0.354 | 0.00029 |
| MRPL47 | 0.693 | 8.50E-05 |
| MRPL49 | -0.606 | 0.00067 |
| MRPL9 | 1.09 | 0.00013 |
| MRPS15 | 0.595 | 1.50E-05 |
| MRPS17 | 0.533 | 0.00081 |
| MRPS18B | -0.967 | 8.80E-05 |
| MRPS25 | 0.442 | 0.00067 |
| MRS2P2 | 0.412 | 1.10E-07 |
| MS4A14 | 0.289 | 0.00019 |
| MSH3 | -1.08 | 3.80E-06 |
| MSH5 | 0.285 | 0.00091 |
| MSI2 | -0.442 | 0.00017 |
| MSL1 | 1.33 | 6.70E-07 |
| MSL3 | 1.07 | 7.70E-05 |
| MSRA | -0.433 | 0.00018 |
| MSTO1 | 0.777 | 2.40E-06 |
| MT1F | 0.21 | 0.00069 |
| MT1X | 0.199 | 0.00049 |
| MT2A | 0.318 | 1.90E-05 |
| MTA2 | 1.38 | 4.30E-06 |
| MTCP1NB | 0.548 | 2.30E-05 |
| MTCP1 | 0.398 | 8.90E-05 |
| MTERFD1 | 0.578 | 0.00079 |
| MTF2 | 1.15 | 1.50E-05 |
| MTFR1 | -0.576 | 0.00026 |
| MTHFD2 | 0.683 | 1.30E-08 |
| MTM1 | -0.711 | 1.90E-07 |
| MTMR10 | -1.06 | 9.00E-06 |
| MTMR12 | -1.17 | 1.20E-08 |
| MTMR4 | 0.589 | 0.00073 |
| MTMR7 | 0.498 | 0.00031 |
| MTSS1 | -0.486 | 9.90E-06 |
| MUC12 | 0.227 | 4.30E-05 |
| MUC5B | 0.201 | 7.10E-06 |
| MURC | 0.467 | 8.70E-07 |
| MUTED | -0.639 | 3.30E-05 |
| MUTYH | 0.712 | 0.00012 |
| MUT | -0.477 | 7.90E-07 |
| MX2 | 0.572 | 9.50E-07 |
| MXD3 | 0.623 | 2.60E-08 |
| MXRA8 | 0.227 | 0.00051 |
| MYADML2 | 0.674 | 1.50E-05 |
| MYBL2 | 0.415 | 3.80E-09 |
| MYBPC3 | 0.42 | 1.20E-05 |
| MYB | 0.272 | 0.00055 |
| MYCBPAP | 0.395 | 1.30E-06 |
| MYCN | -0.301 | 3.00E-04 |
| MYL3 | -0.173 | 8.60E-05 |
| MYLIP | -0.445 | 0.00065 |
| MYLK2 | 0.3 | 0.001 |
| MYO15A | 0.336 | 0.00026 |
| MYO1D | -0.573 | 2.00E-04 |
| MYO5B | -0.368 | 6.80E-05 |
| MYO6 | -0.569 | 9.00E-08 |
| MYO9A | -0.428 | 3.30E-06 |
| MYPN | 0.333 | 0.00098 |
| MYPOP | 0.684 | 6.20E-05 |
| MYRIP | -0.263 | 3.10E-05 |
| MYST1 | 0.854 | 0.00025 |
| MZF1 | 0.48 | 0.00012 |
| N4BP1 | -1.42 | 0.00086 |
| NAA10 | 0.617 | 1.40E-05 |
| NAA30 | -0.666 | 0.00088 |
| NAA38 | 1.03 | 0.00016 |
| NAA40 | 0.862 | 5.70E-06 |
| NAALADL2 | -0.387 | 7.80E-05 |
| NAB2 | 0.51 | 0.00014 |
| NACC1 | 0.884 | 3.50E-06 |
| NADSYN1 | 0.608 | 0.001 |
| NAF1 | -0.934 | 6.60E-08 |
| NAGPA | 0.851 | 3.30E-05 |
| NANS | 0.719 | 0.00046 |
| NAP1L1 | 0.704 | 0.00059 |
| NAPB | 0.748 | 0.00031 |
| NAPEPLD | -0.598 | 0.00072 |
| NAPSA | -0.131 | 0.00042 |
| NARF | 1.2 | 1.20E-09 |
| NASP | 1.05 | 4.20E-07 |
| NAT10 | 1.4 | 8.90E-05 |
| NAT8B | -0.195 | 1.60E-05 |
| NAT8 | -0.102 | 3.00E-04 |
| NAT9 | 0.896 | 6.90E-07 |
| NAV3 | 0.229 | 0.00028 |
| NBEAL1 | -0.321 | 6.70E-05 |
| NBEA | -0.486 | 5.10E-07 |
| NBR1 | -1 | 1.40E-06 |
| NCAPD2 | 0.742 | 2.70E-05 |
| NCAPG | 0.582 | 3.90E-08 |
| NCAPH | 0.437 | 1.20E-05 |
| NCBP2 | 1.11 | 2.50E-05 |
| NCF4 | 0.398 | 0.00021 |
| NCKAP5L | 0.901 | 8.60E-10 |
| NCKAP5 | -0.438 | 1.80E-07 |
| NCLN | 0.755 | 5.40E-05 |
| NCOA1 | -0.914 | 0.00027 |
| NCOA2 | -0.296 | 2.50E-06 |
| NCOA4 | -0.64 | 1.30E-07 |
| NCOA7 | -0.41 | 0.00062 |
| NCRNA00085 | 0.497 | 1.40E-07 |
| NCRNA00105 | 0.247 | 7.50E-06 |
| NCRNA00110 | 0.539 | 2.00E-05 |
| NCRNA00115 | 0.337 | 0.00018 |
| NCRNA00152 | 0.399 | 2.10E-05 |
| NCRNA00174 | 0.369 | 3.10E-05 |
| NCRNA00176 | 0.354 | 0.00019 |
| NCRNA00183 | 0.745 | 0.00012 |
| NCRNA00201 | 0.372 | 4.40E-05 |
| NCRNA00204B | 0.403 | 6.20E-05 |
| NCS1 | 0.594 | 1.50E-06 |
| NDC80 | 0.592 | 3.70E-08 |
| NDFIP2 | -0.792 | 9.60E-07 |
| NDOR1 | 0.7 | 5.60E-06 |
| NDRG2 | -0.416 | 0.00015 |
| NDUFS1 | -0.676 | 1.60E-05 |
| NEBL | -0.347 | 9.40E-08 |
| NECAB3 | 0.349 | 3.00E-04 |
| NEDD9 | -0.343 | 0.00035 |
| NEIL3 | 0.434 | 1.70E-08 |
| NEK2 | 0.398 | 8.10E-06 |
| NEK5 | -0.258 | 0.00024 |
| NFATC2IP | 0.853 | 0.00036 |
| NFE2L3 | 0.385 | 0.00016 |
| NFKBIZ | 0.338 | 1.80E-06 |
| NFX1 | -0.762 | 0.00081 |
| NHLH1 | 0.376 | 0.00082 |
| NHLRC2 | -0.269 | 0.00011 |
| NINJ1 | 0.643 | 0.00022 |
| NIPAL2 | -0.387 | 0.00015 |
| NIPAL3 | -0.762 | 3.60E-06 |
| NIPAL4 | 0.216 | 3.80E-06 |
| NIPSNAP1 | -0.543 | 0.00075 |
| NKX2-2 | 0.269 | 1.00E-05 |
| NLGN2 | 0.553 | 1.20E-05 |
| NLGN3 | 0.362 | 0.00019 |
| NLN | -0.443 | 6.90E-06 |
| NLRP14 | -0.317 | 0.00063 |
| NME1-NME2 | 0.492 | 2.30E-05 |
| NME1 | 0.565 | 6.20E-06 |
| NME2 | 0.604 | 1.00E-05 |
| NME4 | 0.506 | 3.30E-05 |
| NMT1 | 1.46 | 0.00062 |
| NMU | 0.194 | 1.50E-05 |
| NNT | -0.771 | 2.20E-06 |
| NOC4L | 0.589 | 0.00074 |
| NOD2 | 0.291 | 0.00045 |
| NOL12 | 0.833 | 7.50E-07 |
| NOP14 | -0.871 | 0.00091 |
| NOP2 | 0.83 | 1.30E-09 |
| NOP56 | 1.18 | 6.10E-09 |
| NOP58 | 1.04 | 5.80E-05 |
| NOS1 | -0.313 | 0.00048 |
| NPAS2 | 0.381 | 0.00093 |
| NPEPL1 | 0.623 | 1.10E-07 |
| NPFFR2 | 0.272 | 0.00054 |
| NPFF | 0.357 | 1.30E-06 |
| NPHP3 | 0.578 | 1.00E-04 |
| NPIPL3 | 0.383 | 1.40E-08 |
| NPIP | 0.422 | 0.00089 |
| NPLOC4 | 1.15 | 1.00E-05 |
| NPM3 | 0.356 | 0.00053 |
| NPNT | -0.383 | 7.50E-06 |
| NPR2 | 0.441 | 0.00094 |
| NPR3 | -0.292 | 2.40E-10 |
| NPTX1 | 0.189 | 0.00094 |
| NPW | 0.315 | 9.50E-05 |
| NPY6R | -0.214 | 5.80E-07 |
| NR1I2 | 0.25 | 0.00037 |
| NR2C1 | 0.804 | 5.20E-05 |
| NR2C2AP | 0.664 | 0.00028 |
| NR3C2 | -0.543 | 7.80E-10 |
| NRBP1 | 1.46 | 3.40E-06 |
| NRBP2 | 0.319 | 0.00094 |
| NRD1 | 1.44 | 5.00E-06 |
| NRG1 | -0.19 | 0.00092 |
| NRM | 0.544 | 3.00E-04 |
| NRSN2 | 0.829 | 4.00E-06 |
| NSMAF | 0.909 | 0.00038 |
| NSUN5P1 | 0.275 | 4.10E-05 |
| NSUN5P2 | 0.31 | 2.20E-05 |
| NSUN5 | 0.532 | 0.00032 |
| NSUN6 | 0.784 | 1.60E-06 |
| NTM | 0.201 | 0.00091 |
| NTN4 | -0.419 | 1.80E-06 |
| NTN5 | 0.31 | 0.00039 |
| NTNG2 | 0.33 | 1.80E-06 |
| NUDT12 | -0.503 | 0.00033 |
| NUDT1 | 0.532 | 3.70E-05 |
| NUDT3 | 1.32 | 0.00015 |
| NUDT5 | 0.804 | 3.90E-06 |
| NUDT6 | -0.668 | 5.20E-05 |
| NUF2 | 0.54 | 3.00E-09 |
| NUMBL | 0.656 | 4.10E-12 |
| NUP133 | -0.845 | 0.00017 |
| NUP155 | -0.528 | 0.00048 |
| NUP62CL | -0.316 | 0.00035 |
| NUP85 | 1.06 | 2.80E-05 |
| NUPL2 | 0.69 | 0.00065 |
| NUTF2 | 0.841 | 1.80E-07 |
| NVL | 0.998 | 1.00E-04 |
| NXT1 | 0.535 | 0.00042 |
| OASL | 0.31 | 0.00042 |
| OBFC2A | 0.436 | 0.00021 |
| OCLN | -0.237 | 0.00021 |
| ODF2L | 0.695 | 1.80E-05 |
| OFD1 | 0.575 | 0.00047 |
| OGDHL | -0.201 | 1.50E-05 |
| OGFOD2 | 0.598 | 0.00029 |
| OGFR | 0.639 | 3.00E-04 |
| OGT | 0.568 | 9.40E-05 |
| OIP5 | 0.501 | 5.90E-06 |
| OIT3 | -0.258 | 2.70E-06 |
| OLAH | 0.296 | 5.10E-05 |
| OLFML2B | 0.287 | 0.001 |
| OMG | 0.212 | 5.20E-05 |
| ONECUT2 | 0.221 | 1.20E-07 |
| OPA3 | -0.647 | 0.00082 |
| OPN4 | 0.266 | 7.70E-07 |
| OR13A1 | 0.337 | 3.10E-06 |
| OR2B6 | 0.542 | 3.50E-05 |
| OR2T10 | -0.222 | 8.00E-05 |
| ORC6L | 0.553 | 2.30E-09 |
| ORMDL1 | 0.984 | 2.00E-07 |
| OSBPL1A | -0.801 | 8.00E-08 |
| OSBPL7 | 0.567 | 1.70E-05 |
| OSBP | -0.612 | 0.00075 |
| OSCAR | 0.372 | 0.00011 |
| OSCP1 | -0.742 | 0.00011 |
| OSGEP | 0.969 | 5.20E-05 |
| OSM | 0.251 | 0.00017 |
| OST4 | 0.592 | 0.00053 |
| OTOF | 0.546 | 4.60E-10 |
| OTUD7A | -0.454 | 5.90E-09 |
| OTX1 | 0.429 | 6.10E-10 |
| OVOL1 | -0.191 | 0.00068 |
| P2RX6 | 0.258 | 0.00035 |
| P2RY11 | 0.342 | 0.00048 |
| P2RY1 | -0.228 | 8.00E-04 |
| P2RY6 | 0.364 | 5.00E-05 |
| P2RY8 | -0.349 | 4.80E-05 |
| P4HA3 | 0.244 | 0.00034 |
| PA2G4 | 1.27 | 5.70E-06 |
| PABPC1L | 0.367 | 2.20E-09 |
| PABPC4L | -0.376 | 6.80E-05 |
| PABPC4 | 0.882 | 0.00024 |
| PABPN1 | 0.85 | 1.40E-08 |
| PACSIN2 | -0.547 | 9.60E-06 |
| PAEP | 0.179 | 4.50E-09 |
| PAFAH1B1 | -0.95 | 0.00074 |
| PAFAH1B2 | -0.322 | 0.00012 |
| PAFAH1B3 | 0.503 | 5.90E-05 |
| PAFAH2 | -1.07 | 3.70E-05 |
| PAIP2B | -0.441 | 9.50E-08 |
| PANK1 | -0.472 | 2.00E-09 |
| PANX2 | 0.194 | 3.60E-05 |
| PAQR4 | 0.463 | 0.00065 |
| PAQR5 | -0.494 | 3.60E-11 |
| PAQR6 | 0.308 | 2.80E-06 |
| PAR5 | -0.272 | 0.00086 |
| PARD3B | -0.386 | 1.10E-07 |
| PARK2 | -0.36 | 2.00E-06 |
| PARN | 0.927 | 0.00044 |
| PARP12 | 0.965 | 5.70E-06 |
| PARP2 | 0.637 | 0.00092 |
| PARP6 | 1 | 1.60E-07 |
| PARS2 | -0.834 | 4.50E-05 |
| PARVG | 0.36 | 0.00021 |
| PAX2 | -0.192 | 0.00011 |
| PBK | 0.369 | 0.00043 |
| PBLD | -0.326 | 6.70E-06 |
| PBX1 | -0.347 | 0.00026 |
| PBX4 | 0.334 | 7.80E-05 |
| PCBP4 | 0.532 | 2.90E-06 |
| PCCA | -0.755 | 2.60E-09 |
| PCDH17 | -0.247 | 0.00016 |
| PCDHAC1 | -0.243 | 0.00094 |
| PCDHAC2 | -0.21 | 0.00034 |
| PCDHGA9 | -0.188 | 0.00099 |
| PCDHGB6 | -0.264 | 9.60E-05 |
| PCGF1 | 0.8 | 0.00047 |
| PCGF5 | -0.727 | 5.20E-05 |
| PCGF6 | 0.823 | 0.00039 |
| PCK1 | -0.185 | 3.40E-07 |
| PCNXL2 | 0.584 | 9.50E-06 |
| PCOLCE | 0.328 | 2.60E-05 |
| PCYOX1L | 0.864 | 1.60E-06 |
| PCYOX1 | -0.66 | 2.70E-06 |
| PDCD2L | 0.626 | 9.10E-05 |
| PDCD5 | 0.949 | 1.60E-09 |
| PDCD7 | 1.41 | 0.00024 |
| PDE2A | -0.293 | 0.00021 |
| PDE4D | -0.698 | 2.20E-06 |
| PDE6D | 0.833 | 0.00052 |
| PDE7B | -0.45 | 1.10E-06 |
| PDGFD | -0.388 | 3.20E-07 |
| PDGFRL | 0.243 | 1.00E-05 |
| PDIA2 | 0.252 | 3.70E-07 |
| PDIA3 | 0.753 | 0.00038 |
| PDIA4 | 0.497 | 0.00052 |
| PDK4 | -0.304 | 5.00E-08 |
| PDLIM7 | 0.472 | 9.00E-06 |
| PDP2 | -0.542 | 2.50E-05 |
| PDPK1 | -0.481 | 0.00011 |
| PDXDC2 | 0.292 | 0.00071 |
| PDZD11 | 0.608 | 0.00032 |
| PDZD2 | -0.254 | 0.00016 |
| PDZD8 | -0.422 | 1.90E-05 |
| PDZK1P1 | -0.161 | 8.40E-05 |
| PDZK1 | -0.198 | 1.50E-05 |
| PEBP4 | -0.449 | 4.60E-05 |
| PECI | -0.799 | 0.00013 |
| PECR | -0.415 | 0.00023 |
| PELO | -1.08 | 0.00011 |
| PER3 | -0.393 | 8.20E-05 |
| PEX11A | -0.677 | 0.00012 |
| PEX12 | -0.588 | 0.00075 |
| PEX19 | -0.921 | 1.20E-05 |
| PEX1 | -0.485 | 5.20E-05 |
| PEX26 | -0.656 | 1.60E-05 |
| PEX5L | 0.281 | 1.40E-06 |
| PEX6 | 0.527 | 9.00E-04 |
| PFDN2 | 0.9 | 2.20E-08 |
| PFDN4 | 0.543 | 1.20E-05 |
| PFDN6 | 0.591 | 1.90E-05 |
| PFN1 | 0.727 | 3.90E-05 |
| PFN4 | 0.442 | 7.90E-05 |
| PGAM2 | 0.211 | 0.00059 |
| PGAM4 | -0.439 | 3.60E-06 |
| PGK1 | -0.633 | 4.70E-06 |
| PGLS | 0.594 | 8.40E-05 |
| PGM5 | -0.328 | 0.001 |
| PGPEP1 | -0.416 | 7.20E-05 |
| PGS1 | 0.947 | 4.30E-05 |
| PHACTR2 | -0.525 | 0.00027 |
| PHAX | -1.05 | 1.40E-06 |
| PHF13 | 0.855 | 0.00011 |
| PHF15 | -0.706 | 0.00014 |
| PHF19 | 0.869 | 9.50E-08 |
| PHF21A | 1.6 | 2.90E-08 |
| PHKB | -1.43 | 6.70E-07 |
| PHKG2 | 0.738 | 1.30E-05 |
| PHLDB1 | 0.579 | 0.00096 |
| PHLDB2 | -0.55 | 5.10E-06 |
| PHOSPHO2 | -0.564 | 1.90E-07 |
| PHYHIPL | -0.245 | 2.20E-06 |
| PHYHIP | 0.255 | 0.00083 |
| PHYH | -0.563 | 0.00023 |
| PI15 | 0.151 | 0.00041 |
| PI3 | 0.122 | 0.00024 |
| PIAS1 | -0.69 | 0.00019 |
| PIAS3 | 0.96 | 9.70E-05 |
| PIF1 | 0.498 | 2.90E-10 |
| PIGL | 0.456 | 0.00021 |
| PIGZ | 0.403 | 9.40E-06 |
| PIK3C3 | -0.924 | 3.90E-05 |
| PIK3R3 | -0.419 | 0.00018 |
| PIK3R4 | -0.495 | 0.00049 |
| PILRB | 0.269 | 2.00E-04 |
| PIM1 | 0.482 | 1.40E-05 |
| PIM2 | 0.328 | 0.00045 |
| PINK1 | -1.04 | 1.30E-09 |
| PIP5K1A | 0.908 | 0.00053 |
| PIP5K1B | -0.293 | 3.00E-04 |
| PIP5KL1 | 0.378 | 1.70E-06 |
| PISD | 0.595 | 0.00019 |
| PITX1 | 0.173 | 3.20E-06 |
| PJA1 | -0.64 | 0.00056 |
| PJA2 | -0.632 | 0.00017 |
| PKD1L2 | 0.216 | 9.60E-05 |
| PKHD1 | -0.173 | 1.60E-06 |
| PKLR | -0.122 | 0.00031 |
| PKMYT1 | 0.515 | 8.20E-07 |
| PKP2 | -0.303 | 3.00E-05 |
| PKP3 | 0.165 | 3.60E-06 |
| PKP4 | -0.646 | 1.00E-05 |
| PL-5283 | 0.789 | 2.60E-05 |
| PLA2G2A | 0.151 | 7.90E-06 |
| PLA2G4D | 0.188 | 0.00027 |
| PLA2G6 | 0.328 | 0.00033 |
| PLAC1 | 0.481 | 2.90E-09 |
| PLAC2 | 0.42 | 2.80E-07 |
| PLAC4 | 0.213 | 0.00025 |
| PLAC8L1 | 0.365 | 4.50E-05 |
| PLAUR | 0.414 | 2.00E-06 |
| PLB1 | 0.365 | 0.00057 |
| PLCL1 | -0.416 | 2.70E-07 |
| PLCL2 | -0.687 | 2.30E-08 |
| PLCXD1 | 0.53 | 1.00E-08 |
| PLEKHA1 | -0.512 | 0.00036 |
| PLEKHA7 | -0.434 | 4.50E-08 |
| PLEKHA9 | 0.753 | 1.20E-07 |
| PLEKHF1 | 0.361 | 0.001 |
| PLEKHF2 | -0.893 | 1.90E-05 |
| PLEKHG2 | 0.465 | 0.00069 |
| PLEKHG4B | 0.211 | 3.20E-05 |
| PLEKHG4 | 0.296 | 0.00081 |
| PLEKHM1P | 0.402 | 0.00085 |
| PLEKHM3 | -0.407 | 8.00E-04 |
| PLEKHO1 | 0.531 | 1.10E-06 |
| PLG | -0.129 | 6.80E-05 |
| PLIN3 | 0.525 | 0.00081 |
| PLK1 | 0.534 | 2.60E-10 |
| PLLP | -0.35 | 0.00014 |
| PLOD2 | 0.381 | 0.00012 |
| PLP2 | 0.42 | 2.80E-05 |
| PLRG1 | -1.15 | 7.00E-04 |
| PLS1 | -0.358 | 3.80E-09 |
| PLTP | 0.284 | 3.50E-06 |
| PLVAP | -0.271 | 0.00043 |
| PLXNA1 | 0.748 | 2.10E-06 |
| PLXNA2 | -0.332 | 0.00067 |
| PLXNB3 | 0.369 | 6.10E-09 |
| PMAIP1 | 0.326 | 1.40E-05 |
| PMCH | 0.216 | 0.00021 |
| PMFBP1 | 0.252 | 0.001 |
| PML | 0.954 | 0.00015 |
| PMM2 | 0.698 | 2.20E-05 |
| PNKP | 0.632 | 2.10E-05 |
| PNMA5 | 0.261 | 0.00042 |
| PNMA6A | -0.207 | 4.00E-04 |
| PNPLA4 | -0.672 | 0.00011 |
| POC1A | 0.521 | 9.10E-06 |
| POC1B | -0.475 | 0.00035 |
| PODNL1 | 0.239 | 1.40E-07 |
| PODXL | -0.442 | 8.10E-07 |
| POFUT2 | 0.898 | 1.80E-11 |
| POLA2 | 1.04 | 5.50E-06 |
| POLD1 | 0.83 | 5.50E-05 |
| POLD4 | 0.535 | 2.40E-05 |
| POLE | 0.891 | 2.90E-06 |
| POLM | 0.729 | 1.20E-05 |
| POLQ | 0.393 | 3.60E-05 |
| POLR2C | -0.937 | 5.50E-05 |
| POLR2G | 0.898 | 5.90E-05 |
| POLR2H | 0.998 | 5.50E-08 |
| POLR2J | 0.486 | 0.00038 |
| POLR3G | 0.612 | 1.50E-05 |
| POM121L1P | 0.367 | 0.00058 |
| POM121L9P | 0.267 | 0.00017 |
| PON2 | 0.424 | 0.00044 |
| POP5 | 0.591 | 5.40E-05 |
| POP7 | 0.768 | 8.70E-06 |
| POPDC3 | 0.285 | 6.70E-09 |
| PORCN | 0.493 | 0.00015 |
| POU2F1 | 0.631 | 3.20E-06 |
| POU2F2 | 0.432 | 4.30E-06 |
| POU3F3 | -0.193 | 0.00013 |
| PPAN | 0.559 | 0.00037 |
| PPAP2A | -0.366 | 0.00064 |
| PPAP2B | -0.52 | 1.60E-09 |
| PPARA | -0.645 | 0.00013 |
| PPARGC1A | -0.243 | 4.10E-06 |
| PPEF1 | 0.281 | 5.90E-06 |
| PPFIBP1 | -0.42 | 0.00068 |
| PPIAL4D | 0.46 | 0.00014 |
| PPIB | 0.781 | 7.30E-07 |
| PPIE | 1.15 | 7.00E-06 |
| PPIH | 0.753 | 2.00E-05 |
| PPIL5 | 0.658 | 0.00031 |
| PPM1A | -0.793 | 0.00027 |
| PPM1G | 1.04 | 0.00016 |
| PPM1J | 0.341 | 0.00038 |
| PPM1L | -0.338 | 4.50E-05 |
| PPM1M | 0.696 | 6.60E-05 |
| PPM1N | 0.444 | 2.50E-06 |
| PPP1R13B | -0.591 | 0.00013 |
| PPP1R14B | 0.566 | 4.80E-06 |
| PPP1R16B | -0.277 | 0.00093 |
| PPP1R1A | 0.107 | 0.00035 |
| PPP1R3D | -0.777 | 0.00033 |
| PPP2CB | -0.839 | 2.00E-04 |
| PPP2R2C | 0.23 | 4.60E-08 |
| PPP2R3B | 0.78 | 1.20E-07 |
| PPP2R4 | 0.596 | 0.00076 |
| PPP4C | 0.696 | 0.00076 |
| PPPDE2 | 0.734 | 1.20E-06 |
| PQBP1 | 0.611 | 0.00073 |
| PQLC2 | 0.558 | 0.00027 |
| PRAF2 | 0.562 | 9.10E-06 |
| PRAME | 0.12 | 0.00085 |
| PRC1 | 0.731 | 2.40E-07 |
| PRCC | 0.825 | 0.00066 |
| PRCD | 0.374 | 0.00096 |
| PRCP | -0.882 | 3.00E-05 |
| PRDM15 | 0.618 | 0.00096 |
| PRDM6 | 0.298 | 0.00077 |
| PRDM8 | 0.262 | 0.00046 |
| PRDXDD1P | 0.567 | 1.80E-05 |
| PREPL | -0.833 | 0.00037 |
| PRG4 | 0.195 | 4.90E-06 |
| PRH1 | 0.659 | 2.50E-06 |
| PRH2 | 0.666 | 3.60E-10 |
| PRKAA2 | -0.31 | 2.20E-08 |
| PRKAR2A | -0.416 | 1.70E-07 |
| PRKCE | -0.547 | 1.20E-07 |
| PRKCG | 0.288 | 4.80E-05 |
| PRKD1 | -0.68 | 9.70E-08 |
| PRKRIP1 | 0.817 | 9.50E-06 |
| PRPF39 | 0.632 | 2.60E-05 |
| PRPF40B | 0.487 | 0.00036 |
| PRPF8 | -0.963 | 2.70E-05 |
| PRR19 | 0.498 | 1.60E-06 |
| PRR24 | 0.439 | 3.10E-06 |
| PRR3 | 0.581 | 0.00089 |
| PRRG4 | -0.364 | 1.70E-05 |
| PRRT2 | 0.284 | 2.00E-04 |
| PRRX1 | 0.254 | 4.80E-05 |
| PRSS36 | 0.546 | 1.60E-05 |
| PRSS3 | 0.174 | 6.60E-05 |
| PRSS50 | 0.253 | 8.60E-05 |
| PRSS53 | 0.532 | 1.60E-08 |
| PRSS8 | -0.248 | 0.00048 |
| PRTFDC1 | 0.431 | 0.00048 |
| PSAT1 | 0.214 | 3.30E-05 |
| PSENEN | 0.594 | 2.30E-05 |
| PSMA1 | 0.993 | 0.00022 |
| PSMA3 | 0.755 | 0.00073 |
| PSMA4 | 1.15 | 1.00E-07 |
| PSMA5 | 0.723 | 0.00069 |
| PSMB10 | 0.333 | 0.001 |
| PSMB1 | 0.667 | 0.00066 |
| PSMB2 | 1.19 | 8.20E-06 |
| PSMB3 | 0.508 | 8.10E-05 |
| PSMB4 | 0.876 | 2.90E-05 |
| PSMB6 | 0.552 | 0.00086 |
| PSMB7 | 0.75 | 8.00E-04 |
| PSMC3IP | 0.515 | 0.00081 |
| PSMC4 | 0.739 | 0.00078 |
| PSMC5 | 0.621 | 0.00047 |
| PSMD13 | 1.07 | 2.20E-08 |
| PSMD14 | 0.669 | 0.00054 |
| PSMD4 | 0.72 | 8.00E-04 |
| PSMD7 | 1.27 | 4.80E-06 |
| PSME2 | 0.676 | 5.60E-07 |
| PSMG1 | 0.751 | 1.30E-05 |
| PSMG3 | 0.57 | 3.60E-06 |
| PSMG4 | 0.528 | 0.00041 |
| PSRC1 | 0.83 | 6.50E-11 |
| PTAR1 | -0.503 | 0.00065 |
| PTK6 | 0.226 | 1.70E-05 |
| PTMA | 0.733 | 0.00013 |
| PTPLA | 0.304 | 1.20E-05 |
| PTPLB | -0.256 | 3.30E-05 |
| PTPN14 | -0.394 | 0.00018 |
| PTPN2 | 1.21 | 7.10E-07 |
| PTPRB | -0.457 | 1.20E-09 |
| PTPRG | -0.653 | 2.90E-11 |
| PTPRH | 0.176 | 1.20E-06 |
| PTPRK | -0.56 | 0.00014 |
| PTPRVP | 0.324 | 0.00061 |
| PTRH2 | 0.872 | 5.30E-05 |
| PTTG1 | 0.525 | 3.60E-10 |
| PTTG2 | 0.502 | 0.00059 |
| PTTG3P | 0.497 | 0.00022 |
| PTX3 | 0.315 | 5.00E-06 |
| PURA | -1.02 | 2.00E-08 |
| PUS1 | 0.919 | 6.00E-09 |
| PUSL1 | 0.528 | 3.10E-06 |
| PVRL2 | 0.682 | 0.00024 |
| PVRL3 | -0.258 | 1.10E-05 |
| PVRL4 | 0.176 | 0.00058 |
| PVT1 | 0.464 | 0.00013 |
| PXMP2 | -0.409 | 0.00044 |
| PXN | 0.648 | 0.00067 |
| PYCARD | 0.32 | 0.00025 |
| PYCR1 | 0.313 | 3.30E-06 |
| PYGO2 | 1.25 | 1.10E-06 |
| PYY2 | 0.436 | 1.00E-06 |
| QRFPR | -0.174 | 1.40E-05 |
| QSOX1 | 0.381 | 0.00034 |
| QSOX2 | 1.07 | 4.80E-06 |
| QTRT1 | 0.5 | 0.00021 |
| RAB13 | 0.926 | 6.00E-05 |
| RAB15 | 0.42 | 0.00012 |
| RAB17 | -0.393 | 5.30E-07 |
| RAB19 | -0.354 | 5.00E-07 |
| RAB21 | -1.02 | 1.00E-04 |
| RAB26 | 0.211 | 0.00061 |
| RAB3IP | -0.414 | 2.30E-06 |
| RAB4A | -0.859 | 0.00054 |
| RAB4B | 0.704 | 3.20E-06 |
| RAB5C | -0.783 | 0.00022 |
| RAB8A | 1.16 | 8.90E-05 |
| RABEP2 | 0.506 | 0.00048 |
| RABL2A | 0.779 | 0.00022 |
| RABL3 | -0.866 | 5.00E-05 |
| RAC3 | 0.281 | 0.00012 |
| RAD17 | -1.2 | 5.20E-05 |
| RAD50 | -0.517 | 5.70E-05 |
| RAD51 | 0.445 | 0.00028 |
| RAD52 | 0.517 | 0.00012 |
| RAD54L2 | -0.459 | 2.20E-06 |
| RAD54L | 0.582 | 2.30E-09 |
| RAD9A | 0.728 | 2.80E-08 |
| RAET1K | 0.451 | 4.50E-05 |
| RAG1AP1 | 0.809 | 4.30E-06 |
| RAG1 | -0.333 | 7.90E-07 |
| RAI2 | -0.529 | 2.70E-08 |
| RALBP1 | -0.812 | 0.00025 |
| RALB | -0.665 | 7.50E-05 |
| RALGAPA2 | -0.274 | 0.00061 |
| RAMP3 | -0.283 | 0.00023 |
| RANBP2 | -0.767 | 0.00015 |
| RANGAP1 | 0.618 | 2.10E-05 |
| RANGRF | 0.495 | 0.00051 |
| RAP1B | 0.844 | 0.00024 |
| RAPGEF2 | -0.531 | 3.90E-07 |
| RAPGEF4 | -0.336 | 4.60E-06 |
| RAPGEF5 | -0.38 | 2.20E-05 |
| RAPGEF6 | -0.224 | 0.00015 |
| RAPGEFL1 | 0.631 | 2.10E-09 |
| RAPSN | 0.346 | 6.40E-05 |
| RARB | -0.411 | 6.80E-05 |
| RASEF | -0.282 | 1.40E-07 |
| RASGRF2 | -0.314 | 3.50E-05 |
| RASSF1 | 0.732 | 0.00034 |
| RASSF6 | -0.185 | 0.001 |
| RAVER1 | 0.942 | 6.90E-05 |
| RAVER2 | -0.571 | 4.10E-06 |
| RBBP5 | -0.339 | 0.00021 |
| RBCK1 | 0.806 | 3.20E-09 |
| RBM17 | 1.59 | 1.20E-05 |
| RBM18 | -0.614 | 0.00072 |
| RBM28 | 0.906 | 5.90E-05 |
| RBM33 | 0.593 | 0.001 |
| RBM34 | 1.1 | 2.40E-05 |
| RBM38 | 0.57 | 6.00E-05 |
| RBM39 | 0.711 | 0.00054 |
| RBM46 | 0.259 | 0.00022 |
| RBM47 | -0.529 | 2.70E-07 |
| RBM6 | 0.496 | 4.00E-05 |
| RBM8A | -0.757 | 0.00023 |
| RBMX2 | 0.723 | 9.60E-06 |
| RBPJ | 0.673 | 0.00013 |
| RC3H2 | -0.501 | 2.20E-05 |
| RCAN2 | -0.36 | 7.80E-06 |
| RCBTB2 | -0.68 | 1.10E-06 |
| RCC2 | 1.06 | 4.00E-07 |
| RCCD1 | 0.815 | 4.70E-05 |
| RCE1 | 0.836 | 0.00073 |
| RCHY1 | -0.827 | 6.00E-05 |
| RCN1 | 0.829 | 2.20E-07 |
| RCN3 | 0.447 | 2.50E-07 |
| RDH16 | 0.237 | 9.00E-07 |
| RDX | -0.468 | 0.00012 |
| REC8 | 0.283 | 3.00E-04 |
| RECQL4 | 0.486 | 1.10E-06 |
| RECQL5 | 0.63 | 0.00084 |
| REEP2 | 0.196 | 0.00021 |
| REEP4 | 0.875 | 3.20E-08 |
| RELT | 0.729 | 2.80E-08 |
| REM2 | 0.473 | 1.30E-06 |
| RERG | -0.283 | 0.00099 |
| REST | -0.283 | 3.00E-05 |
| RETSAT | -0.871 | 2.60E-06 |
| RET | 0.24 | 0.00042 |
| RFC1 | -0.687 | 0.00081 |
| RFC4 | 0.8 | 7.90E-08 |
| RFPL3S | 0.437 | 1.00E-06 |
| RGL1 | -0.318 | 0.00047 |
| RGNEF | -0.643 | 1.00E-09 |
| RGN | -0.317 | 8.30E-06 |
| RGP1 | -0.508 | 3.20E-09 |
| RGS10 | 0.443 | 0.00011 |
| RGS11 | 0.298 | 5.40E-05 |
| RGS17 | 0.465 | 1.90E-12 |
| RGS19 | 0.624 | 4.60E-05 |
| RGS20 | 0.296 | 4.20E-07 |
| RGS2 | 0.272 | 0.00024 |
| RGS7BP | -0.213 | 9.80E-05 |
| RHBDD1 | -0.638 | 3.10E-05 |
| RHBDD3 | 0.695 | 1.90E-06 |
| RHBDF1 | 0.714 | 6.20E-06 |
| RHBDF2 | 0.564 | 0.00084 |
| RHBDL2 | 0.433 | 0.00027 |
| RHEBL1 | 0.571 | 7.60E-06 |
| RHOG | 0.803 | 6.30E-06 |
| RHOQ | 0.777 | 1.10E-05 |
| RHOXF1 | 0.288 | 0.00022 |
| RHPN2 | -0.416 | 1.40E-07 |
| RILPL1 | 0.676 | 0.00023 |
| RIMKLA | -0.212 | 9.80E-05 |
| RIN1 | 0.378 | 5.50E-05 |
| RIN2 | -0.814 | 4.30E-07 |
| RIPK2 | 0.758 | 3.30E-06 |
| RIPK4 | -0.301 | 0.00039 |
| RMND5A | -0.718 | 0.00045 |
| RMRP | 0.207 | 0.00034 |
| RNASE2 | 0.321 | 2.20E-05 |
| RNASEH2A | 0.593 | 0.00091 |
| RNASEH2C | 0.486 | 0.00043 |
| RNF10 | 1.07 | 9.60E-05 |
| RNF111 | -0.687 | 0.00016 |
| RNF113B | 0.33 | 0.00098 |
| RNF11 | -0.921 | 0.00013 |
| RNF121 | -1.01 | 0.00011 |
| RNF13 | -0.701 | 8.20E-05 |
| RNF152 | -0.365 | 2.30E-06 |
| RNF166 | 0.736 | 1.10E-05 |
| RNF170 | -0.718 | 0.00088 |
| RNF175 | 0.29 | 4.30E-06 |
| RNF185 | -0.717 | 0.00077 |
| RNF19B | -0.574 | 6.70E-05 |
| RNF207 | 0.314 | 0.00015 |
| RNF20 | -0.989 | 1.70E-07 |
| RNF215 | 0.84 | 4.80E-06 |
| RNF216L | 1.02 | 2.70E-06 |
| RNF220 | 1.24 | 8.00E-05 |
| RNF34 | 1.96 | 3.80E-08 |
| RNF38 | -0.598 | 5.90E-05 |
| RNF7 | 1.08 | 1.80E-06 |
| RNFT2 | 0.358 | 0.00023 |
| RNLS | -0.41 | 0.00063 |
| RNPS1 | 0.978 | 0.00026 |
| ROBLD3 | 0.547 | 1.10E-05 |
| ROBO3 | 0.448 | 1.90E-07 |
| ROCK2 | -0.392 | 5.20E-05 |
| ROM1 | 0.536 | 4.10E-07 |
| ROR2 | 0.186 | 0.00011 |
| RORA | -0.4 | 0.00016 |
| RORC | -0.299 | 5.20E-07 |
| RP1 | 0.257 | 0.00048 |
| RP9P | 0.593 | 9.70E-07 |
| RP9 | 0.706 | 1.50E-06 |
| RPA4 | 0.401 | 0.00015 |
| RPF2 | 0.66 | 0.00051 |
| RPIA | 1.14 | 1.40E-05 |
| RPL12 | 0.554 | 0.00031 |
| RPL22L1 | 0.526 | 1.90E-08 |
| RPL23AP32 | 0.334 | 2.10E-07 |
| RPL23P8 | 0.446 | 0.00018 |
| RPL24 | 0.415 | 0.00094 |
| RPL27A | 0.471 | 1.30E-05 |
| RPL28 | 0.402 | 0.00039 |
| RPL32P3 | 0.436 | 0.00062 |
| RPL35 | 0.389 | 0.00029 |
| RPL36A | 0.328 | 0.00042 |
| RPL37 | 0.506 | 0.00075 |
| RPL38 | 0.491 | 0.00028 |
| RPL39 | 0.296 | 0.00031 |
| RPLP0P2 | 0.311 | 2.80E-06 |
| RPLP1 | 0.596 | 9.30E-06 |
| RPLP2 | 0.393 | 0.00015 |
| RPP14 | -1.03 | 0.00042 |
| RPP21 | 0.497 | 0.00083 |
| RPP30 | 1.03 | 6.80E-05 |
| RPP40 | 0.71 | 6.80E-06 |
| RPPH1 | 0.243 | 1.80E-05 |
| RPRD2 | -0.574 | 0.00059 |
| RPS17 | 0.443 | 0.001 |
| RPS19 | 0.477 | 3.80E-05 |
| RPS20 | 0.483 | 0.00019 |
| RPS24 | 0.619 | 0.00024 |
| RPS2 | 0.601 | 5.00E-05 |
| RPS6KA2 | -0.63 | 2.10E-08 |
| RPS6KA4 | 0.912 | 2.70E-05 |
| RPS6KA5 | -0.38 | 0.00075 |
| RPS7 | 0.543 | 0.00022 |
| RPSAP9 | 0.474 | 0.00015 |
| RRAGD | -0.393 | 0.00035 |
| RRAS | 0.566 | 2.90E-05 |
| RRM2 | 0.462 | 5.00E-07 |
| RRN3P2 | 0.365 | 1.10E-05 |
| RRP7B | 0.436 | 0.00038 |
| RRP8 | 0.661 | 0.00016 |
| RSPH10B2 | 0.42 | 0.00013 |
| RSPO4 | 0.256 | 5.80E-06 |
| RSPRY1 | -0.408 | 5.20E-05 |
| RTEL1 | 0.486 | 0.00011 |
| RTKN2 | 0.446 | 6.90E-05 |
| RTN4R | 0.447 | 1.20E-05 |
| RTP1 | 0.552 | 4.10E-05 |
| RUFY3 | 0.511 | 0.00016 |
| RUFY4 | 0.281 | 6.20E-08 |
| RUNDC2A | -0.37 | 1.90E-07 |
| RUNDC3B | -0.311 | 1.10E-05 |
| RUNX1 | 0.501 | 3.50E-06 |
| RUNX2 | 0.392 | 0.00019 |
| RWDD3 | 1.03 | 0.00015 |
| RYR2 | 0.169 | 0.00014 |
| S100A11 | 0.574 | 8.60E-07 |
| S100A16 | 0.575 | 0.00061 |
| S1PR1 | -0.33 | 1.30E-05 |
| S1PR2 | 0.493 | 3.60E-05 |
| SAA1 | 0.104 | 1.20E-05 |
| SAA2 | 0.0983 | 0.00011 |
| SAA4 | 0.128 | 0.00065 |
| SAAL1 | 0.776 | 0.00018 |
| SAC3D1 | 0.451 | 5.00E-07 |
| SALL1 | -0.225 | 1.20E-06 |
| SALL4 | 0.242 | 9.50E-06 |
| SAMM50 | -1.17 | 1.10E-06 |
| SAP30BP | 0.935 | 0.00068 |
| SAP30L | -1.03 | 1.10E-06 |
| SAPS1 | 0.811 | 0.00048 |
| SARNP | 1.05 | 7.90E-06 |
| SARS2 | 0.7 | 5.10E-05 |
| SART1 | 0.857 | 0.00043 |
| SASH1 | -0.421 | 0.00044 |
| SAT1 | 0.548 | 0.00019 |
| SBNO1 | -0.214 | 0.00039 |
| SBNO2 | 0.671 | 6.00E-05 |
| SBSN | 0.211 | 5.30E-08 |
| SC65 | 0.495 | 7.70E-05 |
| SCAPER | -0.554 | 5.80E-07 |
| SCARNA10 | 0.307 | 1.30E-05 |
| SCARNA12 | 0.511 | 2.00E-08 |
| SCARNA16 | 0.386 | 8.90E-05 |
| SCARNA5 | 0.256 | 0.00014 |
| SCARNA7 | 0.277 | 0.00015 |
| SCARNA9 | 0.357 | 2.00E-05 |
| SCD5 | -0.235 | 0.00068 |
| SCFD2 | -1.08 | 0.00051 |
| SCG5 | 0.193 | 5.00E-05 |
| SCGB1D2 | -0.217 | 0.00023 |
| SCGN | -0.128 | 6.80E-05 |
| SCML1 | 0.437 | 0.00029 |
| SCN4A | -0.207 | 0.00015 |
| SCN4B | -0.305 | 0.00018 |
| SCNM1 | 0.767 | 1.50E-05 |
| SCNN1D | 0.234 | 0.00026 |
| SCO2 | 0.56 | 4.10E-05 |
| SCXB | 0.377 | 0.00015 |
| SDC2 | -0.646 | 0.00035 |
| SDCCAG3 | 0.885 | 4.30E-05 |
| SDHAF2 | 0.772 | 0.00048 |
| SDPR | -0.413 | 5.30E-08 |
| SEC14L1 | -0.5 | 0.00078 |
| SEC23B | -0.465 | 0.00091 |
| SEC24B | -0.817 | 7.30E-06 |
| SEC31B | 0.279 | 7.20E-05 |
| SEC61A1 | 0.899 | 4.70E-05 |
| SEC61A2 | 0.814 | 6.10E-09 |
| SEMA3A | 0.283 | 1.10E-05 |
| SEMA3D | -0.207 | 1.30E-05 |
| SEMA3E | 0.156 | 2.50E-05 |
| SEMA3G | -0.368 | 8.20E-08 |
| SEMA4B | 0.48 | 5.60E-05 |
| SEMA4F | 0.704 | 6.90E-05 |
| SEMA5A | -0.413 | 5.60E-06 |
| SEMA7A | 0.47 | 1.10E-06 |
| SENP5 | 1.01 | 5.50E-05 |
| SENP8 | -0.748 | 1.20E-05 |
| SEPP1 | -0.532 | 8.70E-08 |
| 11-Sep | -0.711 | 0.00013 |
| SERF2 | 0.512 | 4.10E-05 |
| SERINC1 | -0.68 | 6.40E-07 |
| SERINC3 | -0.702 | 0.00022 |
| SERINC4 | 0.413 | 5.10E-06 |
| SERINC5 | -0.238 | 4.00E-05 |
| SERPINA3 | 0.148 | 6.70E-05 |
| SERPINB5 | 0.237 | 0.00014 |
| SERPIND1 | 0.151 | 0.00015 |
| SERPINE1 | 0.198 | 0.00071 |
| SERPINF1 | 0.249 | 2.20E-05 |
| SERPINH1 | 0.632 | 2.00E-06 |
| SERTAD4 | -0.279 | 6.10E-05 |
| SESTD1 | -0.369 | 1.60E-05 |
| SETD3 | -0.944 | 9.70E-06 |
| SETD4 | 0.701 | 8.00E-05 |
| SETD8 | 1.33 | 1.30E-08 |
| SF3A1 | -1.15 | 3.80E-07 |
| SF3B4 | 1.04 | 1.30E-05 |
| SFRP5 | 0.244 | 5.00E-06 |
| SFRS11 | 0.625 | 0.00028 |
| SFRS13A | 0.974 | 0.00099 |
| SFRS16 | 0.5 | 3.00E-04 |
| SFRS17A | 0.501 | 0.00038 |
| SFRS18 | 0.52 | 0.00012 |
| SFRS1 | 1.25 | 0.00032 |
| SFRS2 | 1.06 | 2.30E-06 |
| SFRS4 | 1.04 | 0.00011 |
| SFRS8 | 0.725 | 3.20E-05 |
| SFRS9 | 1.08 | 0.00048 |
| SGCB | -0.482 | 6.90E-05 |
| SGK3 | -0.457 | 0.00019 |
| SGMS1 | -0.944 | 5.70E-06 |
| SGMS2 | -0.448 | 0.00011 |
| SGOL1 | 0.431 | 1.60E-05 |
| SGPP2 | -0.254 | 2.50E-05 |
| SGSH | 0.93 | 9.90E-06 |
| SGSM1 | -0.343 | 5.30E-07 |
| SH2D2A | 0.306 | 2.30E-05 |
| SH2D4A | -0.439 | 0.00046 |
| SH2D6 | 0.657 | 2.50E-06 |
| SH3BGRL2 | -0.545 | 1.40E-05 |
| SH3BGRL3 | 0.575 | 3.90E-08 |
| SH3BP4 | -0.458 | 0.00043 |
| SH3D19 | -0.734 | 1.00E-06 |
| SH3GL3 | 0.268 | 0.00094 |
| SH3RF1 | -0.591 | 0.00038 |
| SH3YL1 | -0.505 | 0.00013 |
| SHANK2 | -0.357 | 0.00024 |
| SHC1 | 0.879 | 2.80E-08 |
| SHC3 | 0.311 | 5.50E-05 |
| SHCBP1 | 0.429 | 2.40E-05 |
| SHE | -0.45 | 8.70E-07 |
| SHFM1 | 0.713 | 2.30E-06 |
| SHISA5 | 0.905 | 8.40E-05 |
| SHISA6 | -0.302 | 0.00029 |
| SHISA9 | -0.217 | 1.10E-06 |
| SHOX2 | 0.462 | 8.00E-13 |
| SHROOM3 | -0.267 | 3.00E-04 |
| SHROOM4 | -0.398 | 3.00E-05 |
| SIGMAR1 | 0.655 | 6.00E-04 |
| SIK2 | -0.793 | 3.70E-06 |
| SIM2 | 0.378 | 1.20E-05 |
| SIPA1 | 0.559 | 0.00082 |
| SIRT7 | 0.978 | 5.60E-08 |
| SIX1 | 0.263 | 0.00028 |
| SIX2 | 0.221 | 0.00027 |
| SIX5 | 0.805 | 8.20E-08 |
| SKA1 | 0.662 | 1.30E-12 |
| SKA3 | 0.581 | 3.10E-07 |
| SKINTL | 0.439 | 3.50E-06 |
| SLAMF9 | 0.247 | 0.00026 |
| SLC10A2 | -0.152 | 8.00E-07 |
| SLC10A6 | -0.297 | 6.00E-06 |
| SLC11A1 | 0.434 | 6.20E-07 |
| SLC12A8 | 0.249 | 3.30E-05 |
| SLC12A9 | 0.691 | 0.00033 |
| SLC13A1 | -0.122 | 1.40E-05 |
| SLC13A4 | 0.25 | 0.00052 |
| SLC16A12 | -0.275 | 1.10E-13 |
| SLC16A9 | -0.143 | 0.00017 |
| SLC17A1 | -0.113 | 0.00087 |
| SLC17A3 | -0.0929 | 0.00075 |
| SLC17A9 | 0.346 | 1.10E-07 |
| SLC18A2 | -0.249 | 0.00049 |
| SLC18A3 | 0.125 | 0.00014 |
| SLC19A1 | 0.487 | 7.50E-05 |
| SLC1A1 | -0.239 | 8.80E-06 |
| SLC1A5 | 0.674 | 4.60E-05 |
| SLC20A1 | 0.746 | 1.00E-08 |
| SLC22A12 | -0.107 | 3.00E-04 |
| SLC22A13 | -0.159 | 0.00088 |
| SLC22A20 | 0.311 | 0.00054 |
| SLC22A24 | -0.279 | 2.40E-06 |
| SLC22A2 | -0.117 | 4.30E-05 |
| SLC22A6 | -0.102 | 0.00026 |
| SLC25A14 | 0.758 | 0.00061 |
| SLC25A19 | 0.792 | 7.60E-07 |
| SLC25A21 | -0.3 | 5.00E-04 |
| SLC25A22 | 0.859 | 5.10E-07 |
| SLC25A23 | -0.55 | 2.30E-05 |
| SLC25A24 | -0.453 | 0.00017 |
| SLC25A29 | 0.449 | 4.80E-05 |
| SLC25A2 | 0.451 | 4.40E-07 |
| SLC25A30 | -0.623 | 3.50E-05 |
| SLC25A35 | 0.522 | 2.10E-05 |
| SLC25A37 | 0.604 | 1.30E-07 |
| SLC25A40 | -0.233 | 0.00089 |
| SLC25A4 | -0.731 | 9.00E-08 |
| SLC26A10 | 0.219 | 4.20E-05 |
| SLC26A6 | 0.578 | 7.90E-08 |
| SLC26A8 | 0.456 | 0.00044 |
| SLC27A2 | -0.242 | 1.60E-08 |
| SLC2A3 | 0.289 | 0.00081 |
| SLC2A6 | 0.527 | 3.30E-07 |
| SLC2A9 | -0.42 | 1.10E-07 |
| SLC30A1 | -0.384 | 6.00E-05 |
| SLC30A4 | -0.453 | 1.00E-06 |
| SLC30A9 | -0.829 | 3.20E-06 |
| SLC33A1 | -0.664 | 0.00027 |
| SLC35A3 | -0.275 | 0.00029 |
| SLC35A5 | -0.6 | 0.00037 |
| SLC35C2 | 0.91 | 1.00E-06 |
| SLC35E4 | 0.855 | 7.90E-07 |
| SLC38A11 | -0.265 | 0.00052 |
| SLC38A5 | 0.221 | 1.70E-08 |
| SLC39A11 | 0.573 | 0.00048 |
| SLC39A5 | -0.107 | 0.00079 |
| SLC39A8 | -0.365 | 0.001 |
| SLC3A1 | -0.25 | 7.00E-11 |
| SLC40A1 | -0.639 | 9.30E-08 |
| SLC43A3 | 0.379 | 0.00074 |
| SLC44A3 | -0.528 | 3.00E-06 |
| SLC44A4 | -0.205 | 4.00E-04 |
| SLC47A1 | -0.158 | 4.00E-06 |
| SLC4A3 | 0.178 | 6.10E-05 |
| SLC4A4 | -0.183 | 0.00048 |
| SLC4A5 | 0.286 | 5.30E-05 |
| SLC5A10 | -0.129 | 4.90E-05 |
| SLC5A12 | -0.116 | 0.00011 |
| SLC5A1 | -0.152 | 2.90E-06 |
| SLC5A6 | 0.826 | 8.10E-08 |
| SLC5A8 | -0.12 | 2.00E-05 |
| SLC6A13 | -0.109 | 5.00E-04 |
| SLC6A19 | -0.136 | 1.70E-06 |
| SLC7A5 | 0.376 | 1.20E-08 |
| SLC7A6 | 0.875 | 9.50E-06 |
| SLC9A11 | 0.202 | 0.00098 |
| SLC9A1 | 0.679 | 5.50E-05 |
| SLC9A5 | 0.421 | 5.00E-06 |
| SLCO2A1 | -0.38 | 1.30E-08 |
| SLCO4C1 | -0.179 | 6.80E-06 |
| SLCO5A1 | 0.309 | 0.00025 |
| SLFNL1 | 0.293 | 0.00076 |
| SLIT1 | 0.38 | 0.00026 |
| SLK | -0.328 | 0.00012 |
| SLMO1 | 0.593 | 1.60E-07 |
| SLN | 0.16 | 5.60E-06 |
| SLU7 | -0.912 | 0.00012 |
| SMARCA2 | -0.757 | 2.00E-06 |
| SMARCD1 | 1.35 | 8.60E-06 |
| SMARCD3 | 0.351 | 0.00027 |
| SMC4 | 0.937 | 7.50E-07 |
| SMCR7L | -0.948 | 6.00E-05 |
| SMCR8 | -0.609 | 5.30E-06 |
| SMPD2 | 0.626 | 3.00E-05 |
| SMPD3 | 0.389 | 0.00059 |
| SMPD4 | 1.4 | 8.70E-09 |
| SMU1 | -0.999 | 0.00062 |
| SMUG1 | 0.658 | 6.70E-05 |
| SNAI1 | 0.287 | 0.00073 |
| SNAPC2 | 0.768 | 5.30E-05 |
| SNF8 | 1.04 | 1.00E-06 |
| SNHG10 | 0.438 | 1.30E-05 |
| SNHG12 | 0.365 | 1.60E-05 |
| SNHG1 | 0.417 | 0.00056 |
| SNHG3-RCC1 | 0.464 | 0.00018 |
| SNHG3 | 0.453 | 1.10E-08 |
| SNN | -0.72 | 0.001 |
| SNORA67 | 0.646 | 5.20E-06 |
| SNORA7B | 0.53 | 3.30E-08 |
| SNORA8 | 0.565 | 5.30E-07 |
| SNORD10 | 0.379 | 1.10E-05 |
| SNORD116-4 | -0.185 | 0.00039 |
| SNORD17 | 0.349 | 2.90E-07 |
| SNRK | -0.54 | 0.00016 |
| SNRNP70 | 0.553 | 2.40E-05 |
| SNRPA1 | 1.25 | 3.50E-09 |
| SNRPA | 0.742 | 7.60E-06 |
| SNRPB | 0.641 | 6.50E-05 |
| SNRPC | 0.683 | 0.00043 |
| SNRPD1 | 0.844 | 0.00085 |
| SNRPD2 | 0.501 | 0.00038 |
| SNRPF | 0.869 | 7.90E-08 |
| SNRPG | 0.536 | 0.00063 |
| SNURF | -0.178 | 2.80E-05 |
| SNX12 | -0.531 | 9.00E-06 |
| SNX18 | -0.59 | 0.00073 |
| SNX21 | 0.546 | 7.50E-05 |
| SNX22 | 0.864 | 1.50E-08 |
| SNX29 | -0.576 | 2.50E-05 |
| SNX2 | -0.755 | 1.10E-05 |
| SNX30 | -0.579 | 1.40E-07 |
| SNX7 | -0.528 | 2.00E-05 |
| SOCS1 | 0.35 | 1.70E-06 |
| SOCS2 | -0.352 | 0.00062 |
| SOCS3 | 0.331 | 3.90E-05 |
| SOCS7 | -0.333 | 0.00014 |
| SORBS1 | -0.414 | 0.00012 |
| SORBS2 | -0.464 | 4.10E-12 |
| SORL1 | -0.476 | 1.80E-06 |
| SOS2 | -0.806 | 0.00013 |
| SOX12 | 0.885 | 7.40E-08 |
| SOX13 | -0.387 | 0.00026 |
| SOX2 | 0.483 | 2.60E-08 |
| SOX6 | -0.243 | 0.00022 |
| SP140L | 0.853 | 2.50E-06 |
| SP2 | -0.868 | 1.80E-05 |
| SPAG9 | -0.73 | 9.80E-05 |
| SPATA13 | -0.366 | 1.40E-05 |
| SPATA18 | -0.332 | 9.90E-08 |
| SPATA21 | 0.289 | 0.00076 |
| SPATA4 | -0.285 | 0.00061 |
| SPATA6 | -0.603 | 6.20E-09 |
| SPATA9 | 0.568 | 3.60E-06 |
| SPATS2 | 0.81 | 0.00057 |
| SPC24 | 0.379 | 0.00015 |
| SPC25 | 0.569 | 5.80E-07 |
| SPDEF | 0.206 | 5.50E-05 |
| SPDYA | 0.591 | 1.90E-10 |
| SPEG | 0.255 | 7.20E-06 |
| SPG20 | -0.783 | 2.30E-05 |
| SPG7 | 0.701 | 0.00066 |
| SPHK1 | 0.391 | 7.40E-07 |
| SPINT1 | -0.348 | 0.00043 |
| SPINT2 | -0.375 | 2.00E-04 |
| SPOCD1 | 0.324 | 5.20E-08 |
| SPON2 | 0.244 | 0.00026 |
| SPOPL | -0.422 | 0.00035 |
| SPRED3 | 0.346 | 7.00E-04 |
| SPRN | 0.442 | 5.50E-05 |
| SPSB2 | 0.508 | 7.80E-05 |
| SPTBN1 | -0.808 | 9.90E-10 |
| SPTLC2 | -0.589 | 3.20E-05 |
| SPTLC3 | -0.461 | 7.20E-08 |
| SPTY2D1 | -0.659 | 0.00024 |
| SR140 | 0.892 | 0.00013 |
| SRA1 | 0.533 | 0.00032 |
| SRC | 0.688 | 3.90E-05 |
| SRD5A3 | 0.703 | 0.00033 |
| SRFBP1 | -0.35 | 0.00073 |
| SRL | -0.392 | 0.00014 |
| SRM | 0.671 | 1.20E-06 |
| SRP19 | 1.24 | 1.30E-07 |
| SRP54 | -0.769 | 0.00065 |
| SRPK3 | 0.248 | 2.60E-05 |
| SRPX2 | 0.191 | 0.00027 |
| SRPX | 0.266 | 9.80E-05 |
| SRRM5 | 0.602 | 2.90E-06 |
| SRRT | 0.927 | 0.00022 |
| SS18L2 | 0.815 | 4.20E-06 |
| SSBP4 | 0.553 | 2.50E-05 |
| SSC5D | 0.24 | 0.00016 |
| SSFA2 | -0.564 | 0.00042 |
| SSNA1 | 0.6 | 0.00018 |
| SSPO | 0.299 | 8.40E-06 |
| SSTR1 | -0.247 | 1.50E-05 |
| ST13 | -1.15 | 4.70E-07 |
| ST3GAL5 | 0.531 | 0.00011 |
| ST6GALNAC3 | -0.381 | 1.10E-05 |
| STAC3 | 0.518 | 6.20E-06 |
| STARD3 | 1.05 | 0.00038 |
| STARD8 | -0.515 | 1.30E-06 |
| STAR | 0.425 | 4.30E-05 |
| STAT2 | 0.789 | 2.50E-08 |
| STAT5B | -0.723 | 7.20E-05 |
| STEAP3 | 0.317 | 4.90E-08 |
| STIL | 0.624 | 7.00E-07 |
| STK31 | 0.311 | 2.30E-07 |
| STK32B | -0.346 | 6.60E-09 |
| STK32C | 0.517 | 5.50E-05 |
| STK35 | 0.951 | 0.00011 |
| STMN4 | 0.819 | 3.00E-05 |
| STON1-GTF2A1L | -0.195 | 0.00048 |
| STRADA | 1.56 | 1.00E-10 |
| STRC | 0.324 | 1.20E-06 |
| STRN | -0.246 | 3.70E-05 |
| STX16 | 0.73 | 4.90E-08 |
| STX1A | 0.815 | 6.10E-12 |
| STX2 | 0.741 | 0.00058 |
| STX4 | 0.93 | 3.20E-05 |
| STYK1 | 0.364 | 1.40E-06 |
| SUCLA2 | -0.913 | 6.90E-09 |
| SUCNR1 | -0.211 | 9.80E-05 |
| SULT1A3 | 0.474 | 3.60E-05 |
| SULT1C4 | -0.247 | 6.10E-07 |
| SULT2B1 | 0.258 | 2.00E-06 |
| SUMO1P1 | 0.663 | 2.60E-06 |
| SUMO2 | 0.913 | 0.00014 |
| SUMO4 | 0.59 | 5.60E-08 |
| SUPT7L | 1.35 | 9.80E-07 |
| SUV39H1 | 0.865 | 5.50E-06 |
| SUV420H2 | 0.63 | 3.90E-08 |
| SUZ12P | 0.776 | 1.60E-10 |
| SWAP70 | -0.516 | 0.00015 |
| SYCE1L | 0.189 | 0.00072 |
| SYCE2 | 0.49 | 5.60E-08 |
| SYDE2 | -0.486 | 2.00E-07 |
| SYN3 | -0.397 | 2.10E-05 |
| SYNC | 0.228 | 4.00E-06 |
| SYNGAP1 | 0.485 | 0.00052 |
| SYNGR4 | 0.297 | 0.00046 |
| SYNJ2BP | -0.818 | 0.00021 |
| SYPL1 | -0.721 | 0.00047 |
| SYT9 | -0.142 | 0.00035 |
| SYTL1 | 0.255 | 0.00021 |
| TACC1 | -0.621 | 7.90E-06 |
| TACC2 | -0.645 | 2.90E-06 |
| TACC3 | 0.672 | 2.60E-08 |
| TAF10 | 0.58 | 1.60E-07 |
| TAF12 | 1.19 | 9.80E-06 |
| TAF1C | 0.523 | 0.00014 |
| TAF3 | -0.745 | 0.00025 |
| TAGLN2 | 0.833 | 8.20E-06 |
| TAL2 | -0.257 | 5.10E-06 |
| TAOK1 | -0.279 | 0.00059 |
| TAPT1 | -0.799 | 0.00025 |
| TARBP2 | 0.697 | 5.80E-07 |
| TAS2R14 | 0.462 | 2.00E-04 |
| TAS2R19 | 0.448 | 0.00051 |
| TAS2R20 | 0.628 | 8.30E-08 |
| TAX1BP3 | 0.584 | 0.00016 |
| TAZ | 0.851 | 4.30E-08 |
| TBC1D15 | -0.951 | 7.60E-06 |
| TBC1D19 | -0.889 | 1.70E-06 |
| TBC1D20 | 1.39 | 2.00E-05 |
| TBC1D26 | 0.373 | 0.00038 |
| TBC1D3B | 0.387 | 6.50E-07 |
| TBC1D3G | 0.132 | 0.00032 |
| TBC1D3 | 0.524 | 1.80E-06 |
| TBC1D4 | -0.573 | 2.70E-05 |
| TBC1D7 | 0.71 | 2.20E-06 |
| TBCB | 0.705 | 7.90E-05 |
| TBL1X | -0.72 | 1.10E-06 |
| TBRG1 | 0.933 | 3.70E-05 |
| TBX10 | 0.446 | 0.00014 |
| TBX15 | 0.266 | 0.00018 |
| TBX18 | 0.25 | 5.10E-05 |
| TBX19 | 0.434 | 0.00034 |
| TBX4 | 0.293 | 0.00071 |
| TCERG1 | 0.902 | 5.20E-07 |
| TCF3 | 0.866 | 2.80E-05 |
| TCIRG1 | 0.678 | 9.90E-09 |
| TCL6 | -0.176 | 5.70E-05 |
| TCOF1 | 0.633 | 1.00E-04 |
| TCTA | -0.644 | 0.00038 |
| TCTE3 | 0.533 | 9.80E-07 |
| TCTEX1D1 | 0.434 | 0.00016 |
| TDG | 1.04 | 5.50E-05 |
| TDRD7 | -0.626 | 0.00011 |
| TEAD3 | 0.717 | 7.40E-06 |
| TEAD4 | 0.683 | 1.30E-05 |
| TEF | -0.421 | 8.80E-05 |
| TEKT5 | 0.384 | 0.00088 |
| TEK | -0.466 | 3.70E-10 |
| TELO2 | 0.891 | 6.60E-06 |
| TERF2 | -1.14 | 3.00E-04 |
| TERT | 0.183 | 2.00E-04 |
| TEX12 | 0.505 | 0.00051 |
| TEX19 | 0.297 | 1.90E-05 |
| TEX2 | -0.524 | 0.00098 |
| TEX9 | -0.276 | 0.00016 |
| TFAP2A | 0.159 | 0.00024 |
| TFAP2E | 0.609 | 4.60E-08 |
| TFE3 | 0.954 | 0.00093 |
| TFR2 | 0.145 | 0.00098 |
| TF | 0.118 | 9.30E-05 |
| TGFBI | 0.163 | 0.00049 |
| TGFBR2 | -0.48 | 2.10E-06 |
| TGFBRAP1 | -0.224 | 8.00E-04 |
| TGM1 | 0.165 | 8.70E-05 |
| TGM4 | 0.529 | 4.30E-06 |
| TGOLN2 | -0.682 | 0.00032 |
| THAP3 | 0.467 | 0.00081 |
| THAP8 | 0.61 | 0.00047 |
| THBS3 | 0.78 | 1.10E-09 |
| THNSL1 | -0.679 | 8.20E-05 |
| THOC1 | 0.946 | 9.30E-07 |
| THOC6 | 0.736 | 1.30E-05 |
| THRA | -0.548 | 0.00026 |
| THRB | -0.337 | 1.80E-05 |
| THSD7A | -0.366 | 1.20E-07 |
| TIA1 | 0.77 | 9.00E-07 |
| TIAF1 | 0.633 | 2.90E-08 |
| TICAM2 | 0.538 | 0.00018 |
| TIGD1 | 0.602 | 4.90E-05 |
| TIMELESS | 0.614 | 0.00028 |
| TIMM16 | 0.38 | 0.00039 |
| TIMP1 | 0.414 | 5.10E-07 |
| TIMP3 | -0.462 | 1.50E-07 |
| TINAG | -0.141 | 6.80E-05 |
| TJP2 | -0.686 | 7.90E-08 |
| TK1 | 0.481 | 3.90E-06 |
| TLCD1 | 0.37 | 8.20E-06 |
| TLL2 | 0.239 | 8.00E-04 |
| TLN2 | -0.31 | 0.00022 |
| TLR3 | -0.208 | 0.00087 |
| TLR9 | 0.401 | 6.10E-05 |
| TM4SF18 | -0.357 | 6.80E-06 |
| TM4SF19 | 0.2 | 4.90E-05 |
| TM9SF2 | -0.521 | 3.00E-06 |
| TMBIM6 | -0.893 | 0.00012 |
| TMC2 | 0.442 | 0.00066 |
| TMCC3 | -0.585 | 1.50E-07 |
| TMCO3 | -0.497 | 0.00083 |
| TMCO6 | 0.643 | 0.00099 |
| TMCO7 | -1.05 | 1.40E-07 |
| TMED4 | -0.838 | 5.00E-06 |
| TMED5 | -0.513 | 0.00036 |
| TMEFF1 | 0.426 | 0.00011 |
| TMEM109 | -0.775 | 0.00098 |
| TMEM125 | -0.217 | 1.20E-05 |
| TMEM132A | 0.386 | 4.10E-05 |
| TMEM135 | -0.703 | 8.20E-05 |
| TMEM138 | 0.963 | 2.70E-07 |
| TMEM139 | -0.287 | 0.00034 |
| TMEM149 | 0.41 | 7.30E-05 |
| TMEM14E | 0.497 | 0.00012 |
| TMEM150C | -0.342 | 1.70E-10 |
| TMEM159 | -0.642 | 1.90E-05 |
| TMEM165 | 0.758 | 5.80E-05 |
| TMEM171 | -0.246 | 9.80E-06 |
| TMEM174 | -0.165 | 6.60E-07 |
| TMEM184C | -0.832 | 1.90E-06 |
| TMEM188 | 0.722 | 0.00096 |
| TMEM189 | 0.903 | 8.00E-10 |
| TMEM192 | -0.827 | 1.40E-08 |
| TMEM214 | 0.805 | 0.00064 |
| TMEM220 | -0.475 | 0.00011 |
| TMEM223 | 0.428 | 0.00052 |
| TMEM232 | -0.383 | 6.10E-06 |
| TMEM233 | -0.276 | 7.00E-04 |
| TMEM25 | -0.387 | 2.40E-05 |
| TMEM27 | -0.251 | 3.60E-10 |
| TMEM38A | -0.283 | 0.00034 |
| TMEM38B | -0.432 | 8.20E-08 |
| TMEM39B | 1.05 | 1.80E-05 |
| TMEM41A | 0.76 | 8.00E-05 |
| TMEM44 | 0.644 | 1.20E-08 |
| TMEM47 | -0.382 | 2.20E-05 |
| TMEM49 | 0.656 | 1.30E-06 |
| TMEM57 | -0.95 | 0.00038 |
| TMEM59 | -0.938 | 0.00044 |
| TMEM66 | -0.888 | 1.70E-06 |
| TMEM69 | 1.33 | 0.00014 |
| TMEM72 | -0.185 | 6.20E-06 |
| TMEM79 | 0.953 | 4.00E-06 |
| TMEM81 | 0.962 | 1.40E-08 |
| TMEM82 | -0.145 | 0.00026 |
| TMEM86B | 0.659 | 2.00E-08 |
| TMEM87B | 0.902 | 8.40E-06 |
| TMLHE | -0.829 | 1.10E-05 |
| TMPRSS13 | 0.238 | 0.00017 |
| TMPRSS6 | 0.171 | 5.00E-04 |
| TMSB10 | 0.456 | 8.10E-05 |
| TMTC1 | -0.382 | 0.00018 |
| TMUB1 | 0.602 | 4.70E-05 |
| TNFAIP2 | 0.438 | 0.00018 |
| TNFRSF10B | 0.678 | 0.00027 |
| TNFRSF11B | -0.243 | 3.70E-05 |
| TNFRSF18 | 0.284 | 0.00012 |
| TNFRSF19 | -0.275 | 6.20E-06 |
| TNFRSF1A | 0.86 | 7.70E-05 |
| TNFRSF21 | -0.344 | 0.00062 |
| TNFRSF25 | 0.282 | 0.00023 |
| TNFRSF6B | 0.211 | 0.00059 |
| TNFRSF8 | 0.373 | 0.00035 |
| TNFSF15 | -0.303 | 2.00E-04 |
| TNFSF4 | 0.483 | 8.80E-05 |
| TNIP2 | 0.943 | 6.70E-06 |
| TNIP3 | 0.24 | 2.50E-05 |
| TNK1 | -0.407 | 8.00E-04 |
| TNK2 | 0.444 | 0.00061 |
| TNNT1 | 0.195 | 2.60E-08 |
| TNRC6A | 0.707 | 0.00024 |
| TNS1 | -0.442 | 2.50E-05 |
| TNS3 | -0.586 | 0.00041 |
| TOB2 | -0.671 | 0.00037 |
| TOLLIP | -0.736 | 0.00038 |
| TOM1L1 | -0.356 | 1.90E-06 |
| TOMM20 | -0.941 | 3.90E-06 |
| TOMM40 | 0.779 | 2.20E-06 |
| TOMM6 | 0.613 | 0.00018 |
| TOP1P2 | 0.657 | 4.60E-06 |
| TOP2A | 0.405 | 4.40E-05 |
| TOR1AIP2 | -0.401 | 1.00E-04 |
| TOR2A | 1.05 | 3.90E-07 |
| TOR3A | 0.884 | 4.20E-06 |
| TP53AIP1 | 0.204 | 0.00033 |
| TP53I13 | 0.59 | 6.10E-05 |
| TP53INP2 | -0.863 | 8.60E-06 |
| TPD52L2 | 1.08 | 7.50E-07 |
| TPM2 | 0.286 | 0.00043 |
| TPM4 | 0.499 | 0.00022 |
| TPRA1 | 0.539 | 0.00048 |
| TPRG1L | -1.13 | 3.30E-08 |
| TPST2 | 0.37 | 4.50E-05 |
| TPTE2 | 0.501 | 8.80E-06 |
| TPX2 | 0.494 | 2.00E-08 |
| TRA2A | 0.917 | 5.00E-06 |
| TRABD | 0.7 | 3.60E-06 |
| TRAF2 | 0.798 | 2.80E-05 |
| TRAF5 | 0.531 | 4.30E-06 |
| TRAF6 | -0.788 | 1.60E-06 |
| TRAF7 | 0.782 | 8.30E-05 |
| TRAIP | 0.711 | 1.10E-08 |
| TRAPPC1 | 0.524 | 0.00056 |
| TREH | -0.173 | 6.90E-05 |
| TREM1 | 0.188 | 0.001 |
| TREML3 | 0.397 | 2.00E-07 |
| TRHDE | -0.199 | 7.80E-08 |
| TRIB3 | 0.387 | 1.30E-07 |
| TRIM11 | 0.985 | 2.60E-05 |
| TRIM27 | 1.26 | 3.40E-05 |
| TRIM2 | -0.468 | 1.80E-05 |
| TRIM36 | 0.482 | 9.10E-07 |
| TRIM44 | -0.496 | 8.00E-05 |
| TRIM45 | 0.51 | 0.00016 |
| TRIM46 | 0.311 | 9.50E-06 |
| TRIM4 | -0.781 | 2.10E-05 |
| TRIM55 | -0.157 | 0.00047 |
| TRIM65 | 1.12 | 2.20E-06 |
| TRIML2 | 0.307 | 1.00E-04 |
| TRIP11 | -0.478 | 0.00021 |
| TRIP13 | 0.535 | 1.20E-06 |
| TRIP4 | -1.24 | 0.00045 |
| TRMT112 | 0.671 | 0.00018 |
| TRMT1 | 0.706 | 4.60E-06 |
| TRMT2A | 0.661 | 9.00E-04 |
| TRMT2B | -0.567 | 7.00E-04 |
| TRMU | 0.565 | 0.00076 |
| TRNP1 | 0.203 | 3.50E-05 |
| TROAP | 0.485 | 7.70E-12 |
| TRPC1 | 0.59 | 0.00021 |
| TRPC4AP | 1.15 | 3.10E-06 |
| TRPM2 | 0.33 | 0.00051 |
| TRPM3 | -0.161 | 0.00011 |
| TRPM8 | 0.205 | 0.00026 |
| TRPV3 | 0.286 | 7.70E-05 |
| TRPV4 | -0.253 | 0.00031 |
| TSC22D1 | -0.582 | 0.00028 |
| TSEN15 | 0.733 | 0.00023 |
| TSEN54 | 0.663 | 0.00053 |
| TSKU | 0.274 | 0.00022 |
| TSNAX | -0.823 | 5.00E-04 |
| TSPAN12 | -0.24 | 0.00098 |
| TSPAN1 | -0.178 | 4.00E-04 |
| TSPAN7 | -0.365 | 4.00E-07 |
| TSPYL1 | -0.932 | 7.40E-08 |
| TSPYL2 | 0.41 | 1.00E-04 |
| TSTA3 | 0.558 | 0.00048 |
| TTC13 | 0.865 | 1.50E-06 |
| TTC19 | -0.943 | 0.00066 |
| TTC21A | 0.333 | 0.00074 |
| TTC22 | -0.243 | 3.80E-06 |
| TTC37 | -0.324 | 0.00028 |
| TTC39B | -0.446 | 1.40E-05 |
| TTC39C | 0.451 | 2.10E-05 |
| TTC9 | -0.449 | 2.60E-05 |
| TTK | 0.461 | 2.10E-06 |
| TTLL11 | -0.587 | 7.40E-05 |
| TTLL13 | 0.49 | 4.20E-05 |
| TTLL3 | 0.395 | 4.10E-06 |
| TTLL4 | 0.746 | 7.00E-05 |
| TTLL9 | 0.353 | 0.00054 |
| TTL | 0.779 | 6.80E-05 |
| TTPAL | 0.824 | 9.90E-06 |
| TTYH1 | 0.344 | 1.20E-07 |
| TUBA1B | 0.771 | 8.30E-06 |
| TUBA1C | 0.579 | 2.20E-05 |
| TUBB3 | 0.293 | 2.50E-06 |
| TUBB4Q | 0.491 | 0.00065 |
| TUBB6 | 0.849 | 1.10E-06 |
| TUBB | 0.796 | 0.00072 |
| TUBGCP4 | -0.73 | 4.40E-05 |
| TWIST1 | 0.303 | 0.00011 |
| TXLNA | 1.24 | 1.40E-06 |
| TXNDC16 | -0.424 | 0.00031 |
| TXNDC3 | 0.371 | 8.60E-05 |
| TXNL4A | 0.624 | 0.00094 |
| U2AF1L4 | 0.562 | 7.60E-06 |
| UBA6 | 0.906 | 3.60E-06 |
| UBA7 | 0.559 | 0.001 |
| UBE2A | 0.839 | 0.00083 |
| UBE2C | 0.425 | 8.40E-12 |
| UBE2F | 0.714 | 0.00021 |
| UBE2I | 1.02 | 0.00023 |
| UBE2O | 1.06 | 7.70E-05 |
| UBE2S | 0.665 | 1.50E-09 |
| UBE2T | 0.618 | 2.40E-07 |
| UBE2V1 | 0.981 | 0.00014 |
| UBE4B | -0.67 | 6.00E-04 |
| UBL4B | 0.356 | 0.00059 |
| UBQLNL | 0.495 | 1.50E-07 |
| UBR1 | -0.593 | 0.00029 |
| UBTD1 | 0.513 | 4.00E-04 |
| UBXN10 | -0.312 | 8.30E-05 |
| UBXN2B | -0.687 | 1.20E-05 |
| UCK2 | 0.787 | 6.10E-08 |
| UCN2 | 0.385 | 2.60E-09 |
| UCN | 0.395 | 1.60E-06 |
| UCP2 | 0.364 | 0.00042 |
| UCP3 | 0.48 | 1.40E-05 |
| UFD1L | 0.915 | 3.20E-05 |
| UFSP2 | -1.12 | 4.70E-07 |
| UGT2A3 | -0.109 | 0.00025 |
| UGT8 | -0.267 | 4.10E-05 |
| UHMK1 | -0.259 | 8.80E-05 |
| UHRF1 | 0.489 | 3.70E-06 |
| ULBP1 | 0.395 | 2.70E-07 |
| ULK3 | 0.468 | 8.00E-04 |
| UNC119B | -0.643 | 3.80E-05 |
| UNC119 | 0.46 | 6.70E-05 |
| UNC13B | -0.558 | 0.00043 |
| UNC13D | 0.478 | 6.60E-07 |
| UPF3B | 0.841 | 1.50E-05 |
| UPK3B | 0.344 | 4.50E-08 |
| UPP1 | 0.294 | 0.00055 |
| UROC1 | 0.372 | 2.10E-06 |
| USF1 | 0.87 | 2.30E-06 |
| USP10 | -0.994 | 0.00061 |
| USP12 | -0.306 | 1.10E-05 |
| USP2 | -0.278 | 0.00017 |
| USP31 | 0.679 | 0.00045 |
| USP35 | 0.682 | 4.50E-05 |
| USP38 | -0.845 | 2.80E-05 |
| USP39 | 0.93 | 0.00019 |
| USP51 | -0.428 | 9.40E-08 |
| USP8 | -0.849 | 4.20E-06 |
| UTP11L | 1.02 | 0.00042 |
| UTP14C | -0.877 | 1.20E-08 |
| UTP6 | 1.39 | 7.30E-05 |
| VAMP1 | 0.433 | 1.00E-06 |
| VAMP3 | -1.15 | 1.20E-05 |
| VASP | 0.751 | 0.00011 |
| VAV3 | -0.456 | 4.00E-08 |
| VCX3A | 0.339 | 0.00011 |
| VIL1 | -0.131 | 4.00E-05 |
| VKORC1 | 0.578 | 0.00059 |
| VMAC | 0.762 | 9.30E-05 |
| VMO1 | 0.36 | 5.00E-05 |
| VNN3 | 0.23 | 0.00064 |
| VOPP1 | 0.66 | 4.10E-05 |
| VPS13B | -0.472 | 0.00079 |
| VPS13D | -0.676 | 6.80E-05 |
| VPS16 | 1.18 | 4.00E-05 |
| VPS24 | -0.922 | 4.00E-05 |
| VPS35 | -1.22 | 1.50E-06 |
| VPS36 | -0.896 | 6.00E-04 |
| VPS37D | -0.361 | 0.00019 |
| VPS41 | -0.806 | 0.00013 |
| VPS45 | 1.24 | 0.00019 |
| VPS4B | -0.951 | 7.00E-05 |
| VRK3 | -0.722 | 0.00088 |
| VSIG10L | 0.495 | 1.40E-05 |
| VSX1 | 0.251 | 7.80E-05 |
| VTN | 0.131 | 0.00057 |
| VWA3A | 0.222 | 0.00036 |
| VWA3B | 0.386 | 1.00E-04 |
| VWF | -0.307 | 1.50E-05 |
| WAC | -0.822 | 0.00032 |
| WARS2 | -0.786 | 0.00043 |
| WASH7P | 0.403 | 0.00071 |
| WASL | -0.745 | 0.00068 |
| WBSCR22 | 0.655 | 0.001 |
| WDFY3 | -0.687 | 2.00E-05 |
| WDR17 | -0.244 | 0.00011 |
| WDR27 | 0.35 | 8.70E-05 |
| WDR31 | -0.326 | 1.20E-06 |
| WDR53 | 1.1 | 1.00E-04 |
| WDR62 | 0.532 | 1.40E-09 |
| WDR72 | -0.223 | 2.70E-07 |
| WDR75 | 1.04 | 0.00014 |
| WDR77 | 0.85 | 0.00056 |
| WDR7 | -0.902 | 5.10E-05 |
| WDR85 | 0.791 | 1.70E-08 |
| WDR8 | 1.1 | 5.00E-07 |
| WDSUB1 | -0.366 | 7.00E-04 |
| WFDC10B | 0.34 | 1.20E-07 |
| WFDC3 | 0.252 | 2.20E-05 |
| WHSC1 | 1.03 | 7.60E-06 |
| WIBG | 0.895 | 4.20E-05 |
| WIPF2 | -0.644 | 1.80E-05 |
| WISP2 | 0.149 | 0.00027 |
| WLS | -0.397 | 3.10E-05 |
| WNT10B | 0.318 | 1.10E-06 |
| WNT16 | 0.428 | 4.10E-07 |
| WNT7B | 0.177 | 0.00018 |
| WSB1 | 0.44 | 2.70E-05 |
| WWC2 | -0.496 | 0.00013 |
| WWP1 | -0.772 | 6.80E-06 |
| WWTR1 | -0.374 | 0.00041 |
| XAF1 | 0.389 | 2.90E-05 |
| XCL1 | 0.225 | 0.00096 |
| XPO6 | 1.17 | 4.80E-05 |
| XPOT | 0.638 | 0.00049 |
| XRCC2 | 0.37 | 0.00071 |
| XRCC3 | 0.64 | 1.10E-07 |
| YARS2 | 1.07 | 1.10E-05 |
| YARS | 1.28 | 2.90E-08 |
| YDJC | 0.498 | 5.90E-05 |
| YIF1B | 0.624 | 4.00E-04 |
| YIPF6 | -0.564 | 1.00E-05 |
| YJEFN3 | 0.258 | 4.90E-05 |
| YKT6 | 0.866 | 1.10E-05 |
| YLPM1 | -0.525 | 0.00023 |
| ZBED3 | -0.438 | 5.90E-05 |
| ZBP1 | 0.227 | 0.00066 |
| ZBTB10 | -0.456 | 6.20E-06 |
| ZBTB16 | -0.297 | 1.00E-05 |
| ZBTB17 | 0.693 | 0.001 |
| ZBTB20 | -0.37 | 0.00031 |
| ZBTB4 | -1.45 | 3.80E-07 |
| ZBTB8OS | 0.965 | 1.40E-06 |
| ZC3H12C | -0.474 | 3.40E-05 |
| ZC3H13 | -0.51 | 0.00011 |
| ZC3H3 | 0.803 | 8.80E-06 |
| ZCCHC12 | 0.346 | 4.90E-06 |
| ZCWPW2 | -0.464 | 8.00E-04 |
| ZDHHC12 | 0.584 | 5.00E-04 |
| ZDHHC15 | -0.255 | 4.50E-05 |
| ZDHHC16 | 0.91 | 0.00037 |
| ZDHHC18 | 1.26 | 2.10E-07 |
| ZDHHC20 | -0.23 | 4.00E-04 |
| ZDHHC21 | -0.33 | 0.00058 |
| ZDHHC24 | 0.549 | 0.00013 |
| ZDHHC6 | -0.989 | 0.00094 |
| ZDHHC9 | -0.621 | 0.00018 |
| ZFAT | -0.706 | 3.40E-06 |
| ZFHX4 | 0.192 | 0.00055 |
| ZFP112 | -0.609 | 4.00E-04 |
| ZFP3 | -0.51 | 0.00031 |
| ZFP82 | -0.514 | 0.00067 |
| ZFP91 | -0.594 | 0.00037 |
| ZFYVE9 | -0.747 | 6.30E-07 |
| ZGLP1 | 0.443 | 1.10E-07 |
| ZGPAT | 0.6 | 0.00027 |
| ZIC2 | 0.283 | 2.00E-09 |
| ZIK1 | -0.502 | 1.60E-07 |
| ZMYND11 | -0.813 | 1.30E-05 |
| ZMYND19 | 0.753 | 3.10E-06 |
| ZMYND8 | 0.59 | 0.00048 |
| ZNF117 | 0.668 | 1.50E-07 |
| ZNF132 | -0.558 | 3.10E-05 |
| ZNF133 | 0.679 | 0.00015 |
| ZNF134 | -0.475 | 1.00E-04 |
| ZNF136 | -0.632 | 0.00049 |
| ZNF14 | -1.03 | 2.70E-06 |
| ZNF184 | -0.563 | 0.00038 |
| ZNF189 | -0.382 | 0.001 |
| ZNF18 | 1.07 | 3.30E-09 |
| ZNF193 | 0.867 | 2.40E-05 |
| ZNF202 | 0.871 | 4.90E-05 |
| ZNF204P | -0.236 | 0.00093 |
| ZNF207 | 1.73 | 5.10E-05 |
| ZNF23 | 0.688 | 0.00073 |
| ZNF251 | 0.717 | 3.70E-05 |
| ZNF252 | -0.985 | 3.20E-06 |
| ZNF253 | -0.631 | 0.00083 |
| ZNF266 | 0.482 | 0.00016 |
| ZNF26 | 0.753 | 1.50E-08 |
| ZNF276 | 0.494 | 0.00014 |
| ZNF280C | 0.729 | 5.00E-04 |
| ZNF287 | -0.62 | 5.00E-04 |
| ZNF28 | -0.497 | 0.00054 |
| ZNF296 | 0.362 | 0.00021 |
| ZNF304 | -0.849 | 4.50E-07 |
| ZNF329 | -0.608 | 3.70E-05 |
| ZNF335 | 0.704 | 6.10E-05 |
| ZNF337 | 0.639 | 4.20E-06 |
| ZNF33B | -0.543 | 3.10E-05 |
| ZNF341 | 0.822 | 2.80E-06 |
| ZNF354C | -0.233 | 0.00031 |
| ZNF365 | 0.221 | 2.20E-05 |
| ZNF366 | -0.368 | 3.10E-09 |
| ZNF37B | 0.394 | 0.00035 |
| ZNF385A | 0.459 | 1.40E-05 |
| ZNF396 | -0.454 | 0.00018 |
| ZNF404 | 0.329 | 9.30E-05 |
| ZNF416 | -0.699 | 9.70E-05 |
| ZNF420 | -0.552 | 1.40E-06 |
| ZNF425 | -0.849 | 3.50E-05 |
| ZNF426 | -0.376 | 3.60E-06 |
| ZNF428 | 0.577 | 0.00035 |
| ZNF430 | -0.417 | 0.00091 |
| ZNF444 | 0.452 | 0.00068 |
| ZNF446 | 0.674 | 0.00012 |
| ZNF474 | 0.379 | 0.00014 |
| ZNF510 | -0.601 | 0.00093 |
| ZNF513 | 0.708 | 1.90E-05 |
| ZNF518B | -0.396 | 0.00035 |
| ZNF542 | -0.407 | 8.40E-05 |
| ZNF543 | -0.819 | 9.90E-06 |
| ZNF549 | -0.414 | 0.00086 |
| ZNF555 | -0.971 | 4.00E-05 |
| ZNF558 | 0.606 | 0.00043 |
| ZNF561 | -0.847 | 8.00E-04 |
| ZNF568 | -0.481 | 0.00029 |
| ZNF570 | -0.408 | 3.90E-05 |
| ZNF579 | 0.354 | 0.00041 |
| ZNF580 | 0.515 | 4.20E-05 |
| ZNF582 | -0.477 | 5.00E-04 |
| ZNF583 | -0.432 | 4.90E-05 |
| ZNF597 | -0.52 | 0.00022 |
| ZNF598 | 0.977 | 9.50E-05 |
| ZNF600 | 0.732 | 2.80E-05 |
| ZNF649 | -0.54 | 2.40E-05 |
| ZNF668 | 0.733 | 2.50E-05 |
| ZNF673 | 0.864 | 8.10E-05 |
| ZNF677 | -0.475 | 2.30E-05 |
| ZNF682 | 0.509 | 1.00E-04 |
| ZNF689 | -0.601 | 2.60E-07 |
| ZNF692 | 0.443 | 1.20E-06 |
| ZNF699 | -0.339 | 2.20E-05 |
| ZNF700 | 0.69 | 0.00014 |
| ZNF704 | -0.281 | 7.70E-08 |
| ZNF706 | 0.91 | 0.00014 |
| ZNF718 | -0.683 | 2.20E-05 |
| ZNF732 | 0.446 | 2.00E-04 |
| ZNF738 | 0.602 | 0.00081 |
| ZNF746 | 1.21 | 2.10E-06 |
| ZNF761 | 0.611 | 0.00092 |
| ZNF767 | 0.357 | 0.00034 |
| ZNF770 | -0.692 | 0.00036 |
| ZNF772 | -0.457 | 0.00033 |
| ZNF778 | -0.629 | 0.00055 |
| ZNF787 | 0.545 | 9.00E-04 |
| ZNF789 | 0.421 | 6.90E-05 |
| ZNF791 | -0.558 | 0.00011 |
| ZNF805 | 0.95 | 1.10E-05 |
| ZNF80 | 0.264 | 0.00021 |
| ZNF813 | -0.412 | 5.50E-07 |
| ZNF814 | 0.432 | 8.00E-04 |
| ZNF828 | -0.555 | 5.10E-05 |
| ZNF835 | -0.321 | 4.70E-05 |
| ZNF836 | -0.522 | 6.70E-05 |
| ZNF83 | 0.507 | 8.80E-06 |
| ZNF841 | 0.446 | 0.00029 |
| ZNF843 | -0.405 | 0.00066 |
| ZNF844 | -0.469 | 1.20E-05 |
| ZNHIT1 | 0.409 | 0.00041 |
| ZNRF1 | 0.828 | 5.50E-06 |
| ZNRF3 | -0.547 | 1.20E-05 |
| ZP1 | 0.165 | 0.00014 |
| ZRANB2 | 0.863 | 2.00E-05 |
| ZRSR2 | 0.729 | 6.80E-05 |
| ZSWIM5 | -0.343 | 3.90E-06 |
| ZXDA | -0.777 | 7.90E-08 |
| ZYG11B | -0.79 | 6.00E-05 |
| ZYX | 0.67 | 0.00032 |
