# Supplementary material for: A Five-Gene Signature Predicts Prognosis in Patients with Kidney Renal Clear Cell Carcinoma
Source: Comput Math Methods Med. 2015 Oct 11;2015:842784. doi: 10.1155/2015/842784 (PMC4619904; doi:10.1155/2015/842784)
Supplement: Supplementary file 1 — Table S1 The sample list and information of patients in training set and testing set. Table S2 Univariable Cox regression analysis with a significance level of 0.001 reveal significant relation between gene expression and survival time. Figure S1 Kaplan-Meier curves analysis of the different clinical stages in the testing set. The two-sided log-rank test were used to determine the survival differences. Figure S2 Receiver operating characteristic (ROC) analysis of sensitivity and specificity by five-gene model in predicting survival time of patient with different clinical stages in the testing set. [file 842784.f1.zip › Supplemental Figure.doc]

**Fig. S1**

**Fig. S1** Survival information of individual gene in training set and testing set. (A) Five genes in training set. (B) Five genes in testing set. The two-sided log-rank test were used to determine the survival differences between the two curves.

**Fig. S2**

**Fig. S2** Receiver operating characteristic (ROC) analysis of sensitivity and specificity by five-gene risk score in predicting survival time of patient with different clinical stages.
